# Mismatch repair deficiency drives malignant progression and alters the tumor immune microenvironment in glioblastoma models

**DOI:** 10.1172/JCI195189

**Published:** 2025-12-23

**Authors:** Montserrat Puigdelloses Vallcorba, Nishant Soni, Seung-Won Choi, Kavita Rawat, Tanvi Joshi, Sam Friedman, Alice Buonfiglioli, Angelo Angione, Zhihong Chen, Gonzalo Piñero, Gabrielle Price, Mehek Dedhia, Raina Roche, Emir Radkevich, Anne M. Bowcock, Deepti Bhatt, Winfried Edelmann, Robert M. Samstein, Timothy E. Richardson, Nadejda M. Tsankova, Alexander M. Tsankov, Ranjit S. Bindra, Raul Rabadan, Juan C. Vasquez, Dolores Hambardzumyan

**Affiliations:** 1 Departments of Oncological Sciences and Neurosurgery, The Tisch Cancer Institute and; 2 Department of Genetics and Genomic Sciences, Icahn School of Medicine at Mount Sinai, New York, New York, USA.; 3 Departments of Systems Biology and Biomedical Informatics, Columbia University, New York, New York, USA.; 4 Department of Neurosurgery, Perelman School of Medicine, University of Pennsylvania, Philadelphia, Pennsylvania, USA.; 5 Yale Center for Research Computing, Yale School of Medicine, New Haven, Connecticut, USA.; 6 Human Immune Monitoring Center and; 7 Lipschultz Precision Immunology Institute, Icahn School of Medicine at Mount Sinai, New York, New York, USA.; 8 Department of Pediatrics, Yale School of Medicine, New Haven, Connecticut, USA.; 9 Department of Cell Biology, Albert Einstein College of Medicine, New York, New York, USA.; 10 Tisch Cancer Institute and; 11 Department of Pathology, Molecular and Cell-Based Medicine, Icahn School of Medicine at Mount Sinai, New York, New York, USA.; 12 Department of Pathology and; 13 Department of Therapeutic Radiology, Yale School of Medicine, New Haven, Connecticut, USA.

**Keywords:** Cell biology, Immunology, Neuroscience, Oncology, Brain cancer, DNA repair, Immunotherapy

## Abstract

Mutations in DNA mismatch repair (MMR) pathway genes (*MSH2*, *MSH6*, *MLH1*, and *PMS2*) are linked to acquired resistance to temozolomide (TMZ) and high tumor mutation burden (TMB) in high-grade gliomas (HGGs), including glioblastomas (GBMs). However, the specific roles of individual MMR genes in the initiation, progression, TMB, microsatellite instability (MSI), and resistance to TMZ in gliomas remain unclear. Here, we developed de novo mouse models of germline and somatic MMR-deficient (MMRd) HGGs. Surprisingly, loss of *Msh2* or *Msh6* did not lead to high TMB, MSI, nor did it confer a response to anti–programmed cell death 1 (anti–PD-1) in GBM. Similarly, human GBM showed discordance between MMR gene mutations and the TMB and MSI. Germline MMRd promoted the progression from low-grade to HGG and reduced survival compared with MMR-proficient (MMRp) tumor–bearing mice. This effect was not tumor cell intrinsic but was associated with MMRd in the tumor immune microenvironment, driving immunosuppressive myeloid programs, reduced lymphoid infiltration, and CD8^+^ T cell exhaustion. Both MMR-reduced (MMRr) and MMRd GBM were resistant to TMZ, unlike MMRp tumors. Our study shows that *N^3^*-(2-fluoroethyl) imidazotetrazine (KL-50), an imidazotetrazine-based DNA targeting agent that induces MMR-independent cross-link–mediated cytotoxicity, was effective against germline and somatic MMRr and MMRd GBMs, offering a potential therapy for TMZ-resistant HGG with MMR alterations.

## Abstract 2

In this work, we found that mismatch repair genes play a role in the tumor growth and temozolomide resistant in glioblastoma, in opposite of KL-50 which sensitizes these tumors.

## Graphical abstract

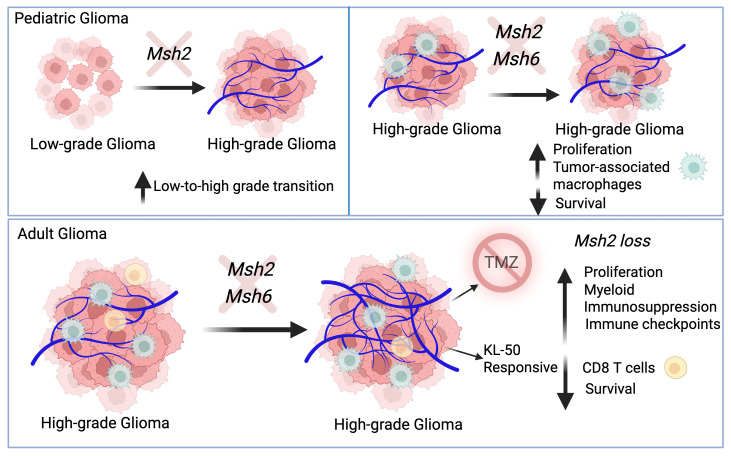

## Introduction

The current standard of care for glioblastoma (GBM) comprises maximal safe surgical resection followed by radiation therapy (RT) and concurrent and adjuvant administration of the alkylating agent temozolomide (TMZ). Despite this multimodal therapeutic regimen, outcomes remain dismal, with a median overall survival of 13–24 months (1) and a 5-year relative survival rate of approximately 6.8% (2). The clinical benefit of TMZ for primary GBM is modest and is restricted to patients whose tumors harbor promoter methylation–mediated silencing of the enzyme *O^6^*-methylguanine-DNA methyltransferase (MGMT), which serves as a predictive biomarker of an initial positive response to TMZ (3). However, patients ultimately experience disease recurrence and develop TMZ resistance (4), leaving few treatment options.

TMZ efficacy depends on an intact DNA mismatch repair (MMR) pathway that is composed of *MSH2*, *MSH6*, *MLH1*, and *PMS2*. In the absence of MGMT, *O^6^*methylguanine (*O^6^*-meG) mispairs with thymine (T), and MMR repeatedly excises thymine in futile cycles that generate single-strand gaps that evolve to double-stranded breaks, leading to cell death (5–8). In response to TMZ treatment, GBMs may acquire subclonal mutations in MMR genes, which prevents the recognition of *O^6^*-meG:T and prevents tumor cell death. Surviving tumor cells accumulate a massive number of DNA mismatches, resulting in a characteristic cytidine-to-thymidine “hypermutation” (HM) phenotype (4, 9).

Monoallelic germline variants in MMR genes result in Lynch syndrome, which predisposes individuals, particularly those with *MSH2* mutations, to various cancers, including brain cancer (10–12). Biallelic germline mutations in MMR genes cause constitutional mismatch repair deficiency (CMMRD), a pediatric cancer predisposition syndrome characterized by early-onset HM tumors, nearly 40% of which are high-grade gliomas (pediatric HGGs [pHGGs]) (13). Despite their resistance to alkylating agents, the biology and therapeutic vulnerabilities of MMR-deficient (MMRd) gliomas remain poorly understood, partially due to the lack of mouse models for Lynch syndrome and CMMRD that develop gliomas rather than extracranial tumors (14, 15).

In sporadic post-TMZ–treated HGGs, MMR mutations are subclonal (4), whereas in CMMRD pHGGs, they are clonal. Both tumor types display HM and resistance to TMZ; however, only the latter responds durably to immune checkpoint inhibition (ICI) (16). Since mutations in MMR pathway genes are rare in primary GBM, their role in causing HM in tumor cells of glial origin remains largely understudied. Moreover, since MMR mutations and HM co-occur after TMZ treatment, it is difficult to know whether a single MMR gene mutation can cause HM, or if the mutation is necessary but not sufficient for creating HM (17).

Here, we elucidated the role of the MMR genes *MSH2* and *MSH6* in the initiation and progression of GBM, HM formation, and responses to TMZ and ICI using genetically engineered mouse models (GEMMs) of de novo gliomas. We show that germline, but not somatic, loss of *Msh2* accelerated GBM progression, remodeled the tumor immune microenvironment toward more immunosuppressive myeloid cells, reduced lymphoid infiltration, increased CD8^+^ T cell exhaustion, and conferred resistance to TMZ. Like most of their human counterparts, *Msh2-*deficient GBMs do not show increased tumor mutational burden (TMB) or microsatellite instability (MSI), and like human sporadic post-TMZ–treated GBMs, these tumors are resistant to anti–programmed cell death 1 (anti–PD-1) treatment, suggesting that subclonal HM and MMR mutations are probably not the cause of ICI failure in GBMs. *Msh2*-deficient GBMs remain sensitive to *N^3^*-(2-fluoroethyl) imidazotetrazine (KL-50), a imidazotetrazine that induces DNA interstrand crosslinks independently of MMR (18–20), revealing therapeutic avenues for patients with Lynch syndrome, CMMRD, or recurrent GBM.

## Results

### Discordance between MMR gene mutations and the HM phenotype in human GBM.

**Figure 1:**
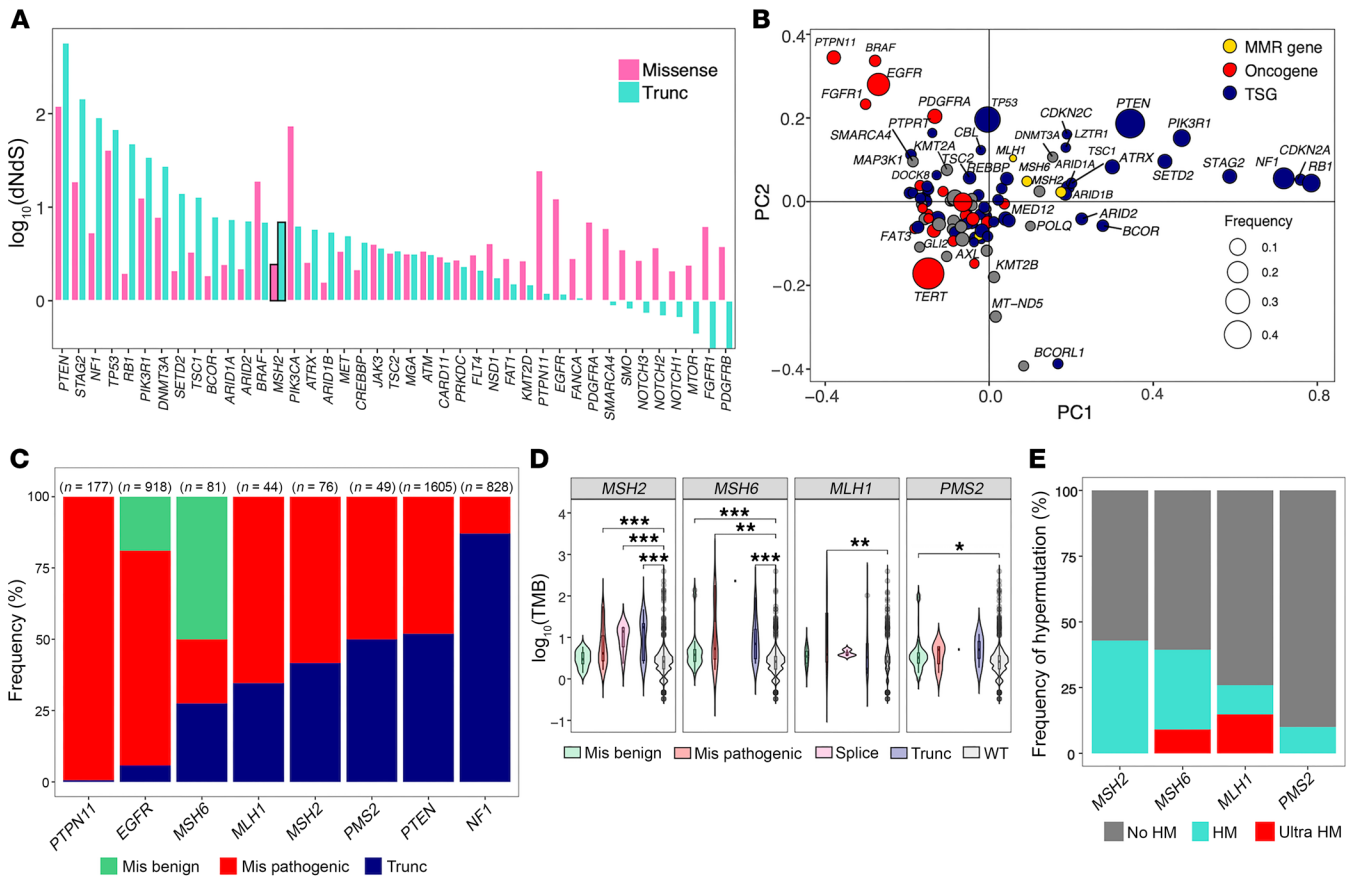
Distinct distributions of mutation types and correlations with TMB highlight the functional divergence of MMR genes in GBM. (**A**) Positive selection of frequently altered genes in GBM tumors. (**B**) Principal component (PC) analysis of frequently altered genes in the GBM cohort based on mutation type representation. (**C**) Proportions of mutation types for each gene. (**D**) Comparison of TMBs according to mutation type for each MMR gene. (**E**) Proportion of the HM phenotype in GBMs with MMR gene mutations. The Kruskal-Wallis test was used, followed by pairwise Wilcoxon rank-sum tests for post hoc analysis. *P* values were adjusted for multiple comparisons using the Bonferroni method (**D**). Data are presented as the mean ± SD. *P < 0.05, **P < 0.01, and ***P < 0.001. Mis, missense; Trunc, truncation.

To characterize the behavior of MMR gene alterations in GBM (*MSH2, MSH6, MLH1,* and *PMS2*) and to evaluate their association with the HM phenotype and MSI, we analyzed genomic data from the AACR project the GENIE version 17.0 dataset (21). The dataset was refined to include only adult isocitrate dehydrogenase (IDH) WT primary GBMs using the provided metadata. This process yielded a final study cohort of 3,742 patients, hereafter referred to as the GENIE-GBM cohort. To investigate the putative driver genes in GBM, we used an analysis based on the nonsynonymous rate/synonymous rate (dN/dS) to identify genes evolving under positive selection. Among the top 100 frequently mutated genes in the GENIE-GBM cohort, 42 genes exhibited a marked signal of positive selection. Notably, *MSH2* was included, suggesting its putative driver function in GBM (Figure 1A). In contrast, *MSH6*, a known contributor to TMZ resistance and HM phenotypes in GBM (4), did not show evidence of positive selection in our analysis (Figure 1A).

Although we did not integrate the functional mutation type in the dN/dS analysis, we observed a clear trend across the positively selected genes. These genes segregated into 2 subsets based on the predominant mutation type observed; some were preferentially altered by missense mutations, while others were enriched for truncating mutations (Figure 1A). This pattern reflects the known functional dichotomy between oncogenes and tumor suppressor genes. Oncogenes typically promote tumorigenesis when activated, often through gain-of-function missense mutations. In contrast, tumor suppressor genes inhibit cancer progression and are typically inactivated by truncating mutations.

We hypothesized that the distribution of mutation types could be used to infer the functional class of each gene. To test this, we examined the mutation spectra (nonsynonymous, truncating, and predicted pathogenic mutations) of the top 100 frequently altered genes (Figure 1B). Genes clustered according to their putative function: tumor suppressors such as *NF1*, *RB1*, and *PTEN* were clearly distinct from oncogenes like *EGFR* and *PDGFRA*. MMR genes, including *MSH2*, had mutation profiles more similar to those of tumor suppressors. Specifically, *MSH2* showed a higher proportion of truncating mutations than did *MSH6*, suggesting that its loss of function may contribute to oncogenesis (Figure 1C and Supplemental Figure 1A; supplemental material available online with this article; https://doi.org/10.1172/JCI195189DS1).

Given the established correlation between MMR gene defects and HM phenotype, particularly for *MSH6* in the context of GBM, we examined the relationship between MMR gene mutations and TMB (Figure 1D and Supplemental Figure 1B). All MMR genes showed significant association with TMB when mutated (adjusted *P* < 0.001 for *MSH2*, *MSH6*, and *PMS2* and 0.002 for *MLH1*); however, the extent of the TMB increase differed by mutation type. Interestingly, for *MSH2*, the most frequently altered MMR gene in the GENIE-GBM cohort, missense mutations predicted to be benign (based on the AlphaMissense score) (22) did not correlate with an increased TMB, whereas the corresponding benign predicted missense mutations in *MSH6* and *PMS2* did. These results suggest that the pathogenic mutations, and not merely the presence of an *MSH2* mutation, are critical for the HM phenotype.

On the basis of these findings, we restricted our downstream analysis to pathogenic mutations (including pathogenic missense, truncating, and splice site mutations). Not all GBMs harboring pathogenic MMR mutations exhibited the HM phenotype, and the incidence of HM varied across different MMR genes (Figure 1E). We next assessed the association between pathogenic MMR mutations and the HM phenotype (Supplemental Figure 1C). All genes except *PMS2* showed association. To identify the most influential MMR gene contributing to HM, we performed multivariate logistic regression analysis (Supplemental Figure 1D) and found that *MSH2* had the strongest association with the HM phenotype, followed by *MSH6*.

We next consulted the dataset of 284 patients with IDH WT GBM from The Cancer Genome Atlas (TCGA) to investigate the association between tumor mutation TMB and mutations in MMR genes (Supplemental Figure 1E). Six patients harbored nonsynonymous mutations in MMR genes or in *POLE/POLD1*. Among these, *MSH6* mutations were the most frequent (5 of 284); however, none of the tumors with *MSH6* mutations exhibited the HM phenotype. Only 2 patients had the HM phenotype (0.7%, 2 of 284). One was an ultra-HM GBM harboring pathogenic missense mutations in both *MLH1* and *POLE*, while the other patient with HM carried no mutations in any of the MMR genes. We observed no correlation between TMB and MSI status. All tumors were microsatellite stable except for 1 case classified as MSI-low (MSI score = 6.96). Overall, these data suggest that *MSH2* may have a potential oncogenic role in GBM. Our analysis further showed that only a subset of GBM samples with MMR gene mutations displayed increased TMB and MSI. Notably, the magnitude of this effect varied depending on the specific MMR gene involved, with the highest levels of TMB observed in tumors harboring *MSH2* mutations.

### Germline loss of Msh2 drives glioma progression from low to high, and the absence of either Msh2 or Msh6 significantly shortens the survival time of mice with HGG.

**Figure 2:**
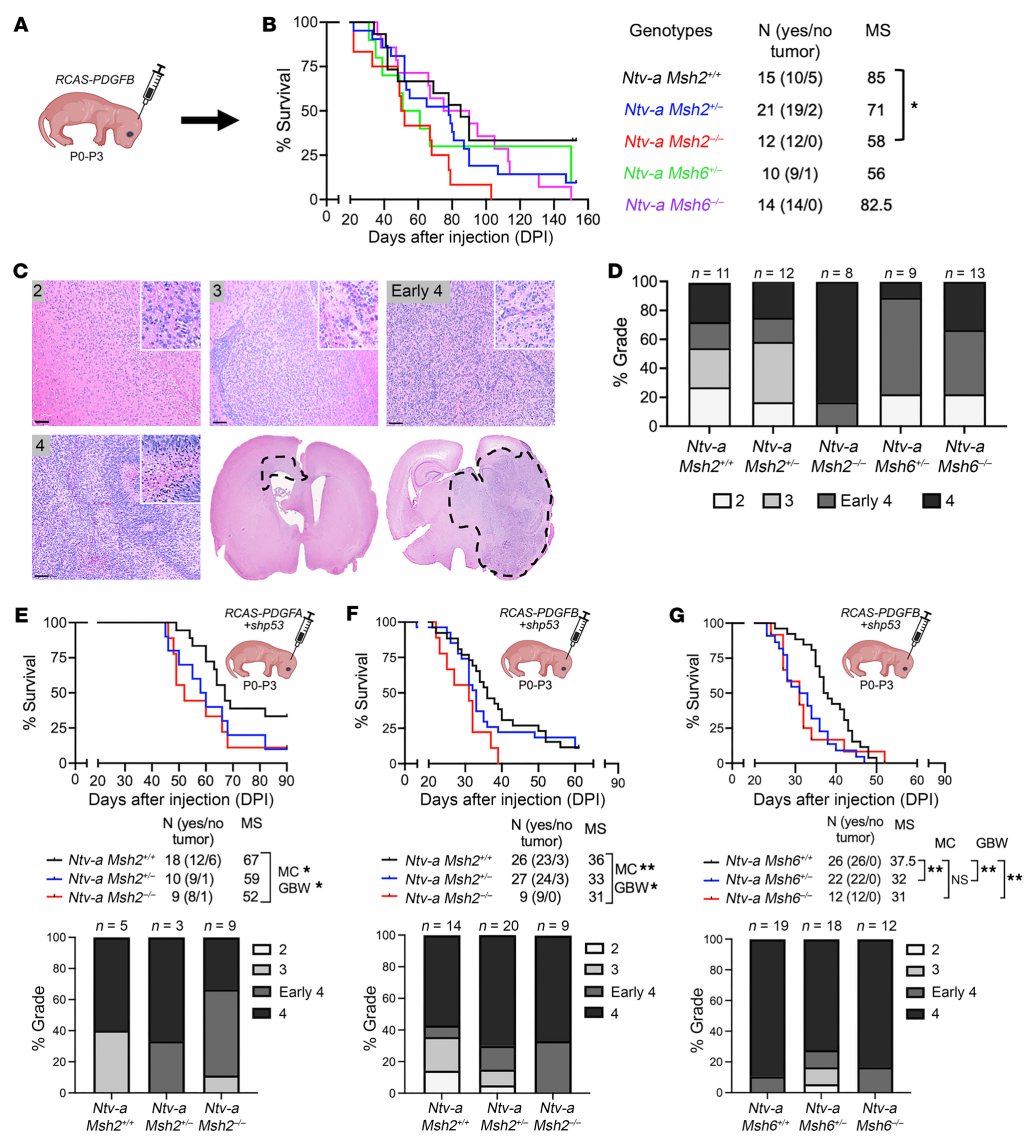
*Msh2* germline loss drives low-grade to high-grade glioma progression. (**A**) Schematic illustration of the generation of gliomas using overexpression of PDGFB in newborn mice. (**B**) Kaplan-Meier survival curves and corresponding median survival (MS) for various genotypes. (**C**) Corresponding H&E-stained tumors of various grades and (**D**) distribution of varying tumor grades in different genotypes. Survival curves and corresponding quantification graphs of tumor grades for *PDGFA* + *shp53* tumors (**E**) and *PDGFB* + *shp53* tumors (**F**) in *Ntv-a* WT, *Ntv-a* *Msh2^+/–^*, and *Ntv-a* *Msh2^–/–^* mice, and (**G**) *PDGFB* + *shp53* tumors in *Ntv-a* WT, *Ntv-a* *Msh6^+/–^*, and *Ntv-a* *Msh6^–/–^* mice. Data represent the mean ± SD. *P < 0.05 and **P < 0.01, by log-rank MC (**E**–**G**) and GBW tests (**B** and **E**–**G**). Panels **A** and **E**–**G** include illustrations created with BioRender.

To investigate the biological relevance of correlative human data implicating *MSH2* as a potential oncogenic factor in GBM, we generated a series of glioma GEMMs, in which the mice had either germline or somatic MMR gene loss. To develop mouse models of gliomas with germline MMRd or reduced MMR (MMRr), we combined the replication-competent avian sarcoma-leukosis virus long terminal repeat with a splice acceptor/tumor virus A (RCAS/Tv-a) system for de novo brain tumor generation (23) with germline *Msh2*- (14) and *Msh6*-deficient (15) mice (mice with null mutations are referred to hereafter as *Msh2^–/–^* and *Msh6^–/–^*). We used *Nestin-tva* (*Ntv-a*) transgenic animals to obtain the following crosses: *Ntv-a* *Msh2^+/+^*, *Ntv-a* *Msh2*^+/–^, *Ntv-a* *Msh2*^–/–^, *Ntv-a* *Msh6^+/+^*, *Ntv-a* *Msh6*^+/–^, and *Ntv-a* *Msh6*^–/–^ mice. Tumors were generated by inducing overexpression of PDGFB using RCAS-PDGFB in *nestin*^+^ stem and progenitor cells in mice at P0–P3 (23) (Figure 2A). Kaplan-Meier survival curves show that homozygous loss of *Msh2*, but not *Msh6*, resulted in increased tumor incidence and shortened survival of tumor-bearing mice (Figure 2B). WT mice developed gliomas with various histological grades, which were equivalent to grade 2 (lacking brisk mitotic activity; <3 mitotic figures in the entire section examined and lacking nuclear atypia, such as pleomorphism, nuclear hyperchromasia, and prominent nucleoli); grade 3 (≥3 mitotic figures detected within the examined tumor sections, but lacking evidence of microvascular proliferation and palisading tumor necrosis); early grade 4 (microvascular proliferation without pseudopalisading necrosis); and grade 4 (microvascular proliferation with pseudopalisading necrosis) IDH WT GBM in humans (24) (Figure 2, C and D). Homozygous loss of *Msh2* (MMRd) resulted in the development of early grade 4 and grade 4 GBMs in mice. These results suggest that loss of *Msh2* resulted in a low- to high-grade transition of gliomas. Next, we used the overexpression of the less potent oncogenic driver PDGFA. Since overexpression of PDGFA alone is insufficient to generate tumors, it was combined with *Tp53* silencing using RCAS-shRNA-p53. Loss of *Msh2* results in shortened survival of tumor-bearing mice (Figure 2E) and increased incidence of early grade 4 and grade 4 HGG (Figure 2E), suggesting that the effect of *MSH2* on low-to-high transition in gliomas was oncogene independent. To determine whether dose-dependent loss of *Msh2* or *Msh6* results in increased growth in HGG-bearing mice, we next generated tumors with RCAS-PDGFB and RCAS-shRNA-p53 in WT, *Msh2*-reduced (heterozygous loss), *Msh6*-reduced, or *Msh6*-deficient mice. Our results indicate that homozygous loss of *Msh2* led to shortened survival of tumor-bearing mice (Figure 2F), whereas both a decrease or loss of *Msh6* resulted in shortened survival of tumor-bearing mice (Figure 2G). In human cells, mismatch recognition is attributed to 2 heterodimeric complexes: MSH2-MSH3 and MSH2-MSH6. Both of these complexes interact with MLH1-PMS2 and are critical for maintaining genomic stability (25, 26). Therefore, *MSH2* is a central MMR gene, because the loss of *MSH2* inactivates the activity of both mismatch recognition heterodimers, so it is expected that *MSH2* deficiency will cause a strong predisposition to cancer in both mice and humans (26). Together with our results showing a greater effect of *Msh2* loss compared with *Msh6* loss during the low-to-high transition and despite the significant effect of losing both on HGG growth, we focused on tumors that were deficient in *Msh2*. IHC staining for MSH2 and MSH6 in tumors generated in *Ntv-a* *Msh2^+/+^*, *Ntv-a* *Msh2*^+/–^, and *Ntv-a* *Msh2*^–/–^ mice showed an absence of MSH2 staining in KO mice and reduced MSH6 staining compared with WT control tumors (Supplemental Figure 2A).

To investigate the degree of changes in tumors due to *Msh2* loss, we performed a series of IHC stainings, which showed no changes in oligodendrocyte transcription factor 2 (OLIG2) or glial fibrillary acidic protein (GFAP) expression levels (Supplemental Figure 2B). However, there was an increase in tumor proliferation in *Msh2*-deficient mice, as assessed by pH3 staining (Supplemental Figure 2B), in accordance with the Kaplan-Meier survival results (Figure 2C). Additionally, we observed no changes in vessel area or size (CD31^+^ cells); however, there was an increase in IBA1^+^ tumor area (IBA1 is a pan-macrophage marker) (Supplemental Figure 2C). These results indicate that loss of *Msh2* promoted increased cell proliferation and enhanced the numbers of tumor-associated macrophages (TAMs) in tumors. This was accompanied by shorter survival times of tumor-bearing mice. These results establish *Msh2* as a key driver of low- to high-grade glioma progression. Additionally, *Msh2* loss promoted increased progression of HGGs through enhanced tumor proliferation and TAM recruitment.

### Germline loss of Msh2, but not Msh6, reduces the survival of GBM-bearing adult mice.

**Figure 3:**
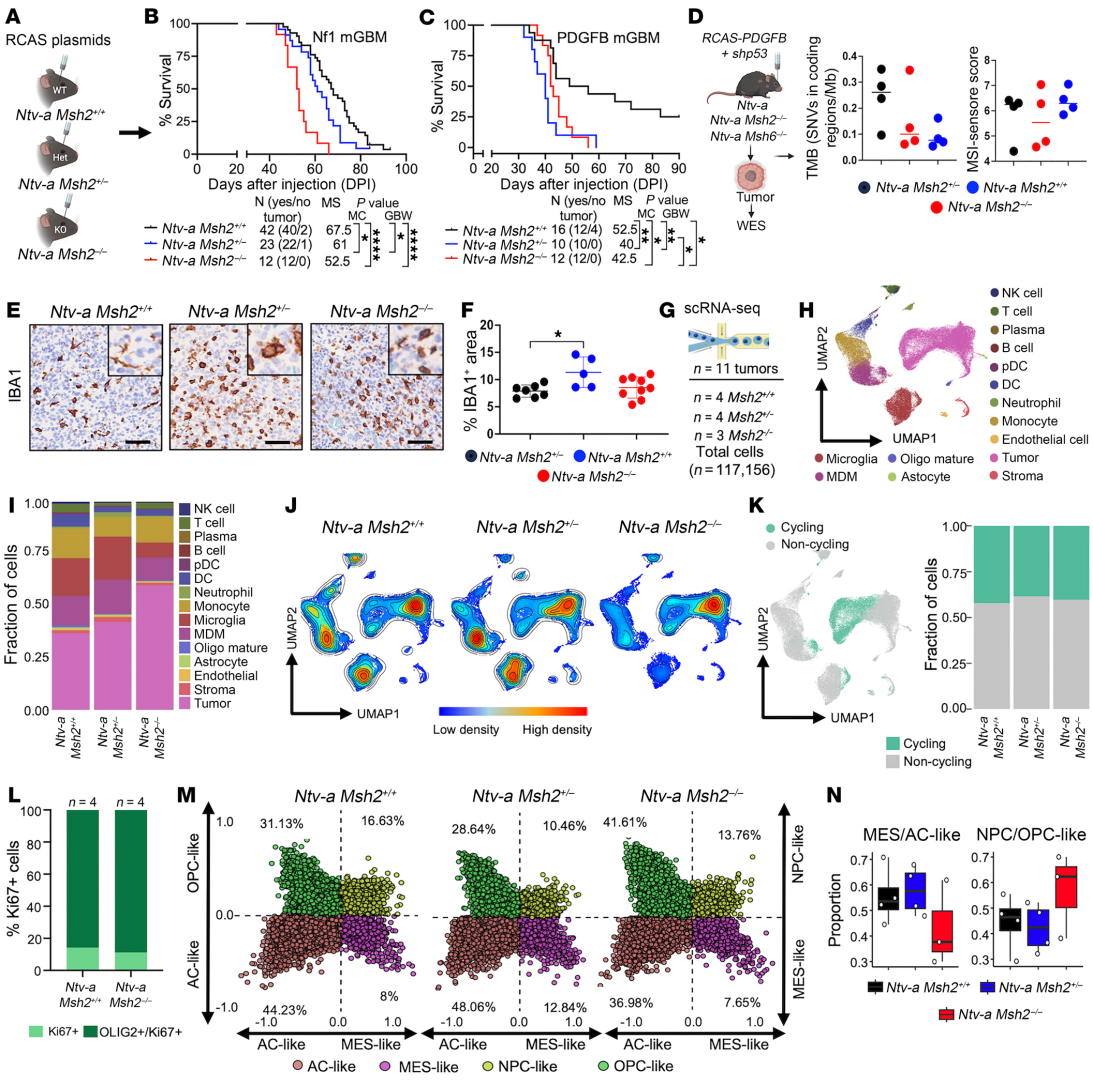
Germline loss of *Msh2* drives GBM progression in adult mice without increasing TMB or MSI. (**A**) Schematic illustration for the generation of adult mGBM models and (**B**) Kaplan-Meier survival curves for Nf1 mGBM and (**C**) PDGFB mGBM. (**D**) Schematic illustration of the experiment for WES. Data show SNVs in coding regions per Mb of sequenced DNA and MSI scores for WT, Msh2, and Msh6-deficient tumors. (**E**) Representative images and (**F**) quantification of IHC for IBA1^+^ TAMs in tumors from **C**. Scale bars: 100 μm and 50 μm (inset). (**G**) Overview of sample processing for scRNA-seq. (**H**) Uniform manifold approximation and projection (UMAP) plot for all nonimmune cell and immune cell scRNA-seq data in this study, colored by cell-type annotation. (**I**) Stacked bar plots depicting the proportion of annotated cells within various genotypes. (**J**) UMAP plots of all cells colored by cell density. Red indicates high cell density, and blue indicates low density. (**K**) UMAP plots of cells colored by cell-cycle status and stacked bar plots depicting the proportion of annotated cells within various genotypes. (**L**) Quantification graph of multiplex IF for Ki67^+^OLIG2^+^ double-positive cells in the Ki67^+^ cell population from tumors. (**M**) 2D quadrant scatter plot representing the GBM cellular state across tumor cells split by *Msh2* status. Orange color indicates AC-like cells, purple color indicates MES-like cells, yellow indicates NPC-like cells, and green indicates OPC-like cells (32). (**N**) Distribution of the proportion of MES- plus AC-like malignant cells (left) and proportion of NPC- plus OPC-like malignant cells (right) across samples split by Msh2 genotypes. Black = WT, blue = *Msh2* heterozygous, and red = *Msh2*-KO. Box plots display the median (central line), interquartile range (box), and whiskers extending to the smallest and largest values within 1.5 times the interquartile range. Individual data points are shown as dots to provide a detailed view of the sample distribution. *P < 0.05, **P < 0.01, and ****P < 0.0001, by MC and GBW tests (**B** and **C**) and 1-way ANOVA followed by Tukey’s post hoc analysis (**F**). Panels **A**, **D** and **G** include illustrations created with BioRender.

To compare the role of germline loss of MMR genes when targeting early *nestin*^+^ cells (P0–P2) versus that in adult mice (6–10 weeks of age), we induced tumors with plasmid RCAS-shRNA-Nf1, RCAS-PDGFA, RCAS-shRNA-p53 (to generate Nf1 murine GBM [mGBM]) or RCAS-PDGFB and RCAS-shRNA-p53 (to generate PDGFB mGBM) combinations in adult *Ntv-a* *Msh2^+/+^*, *Ntv-a* *Msh2^+/–^*, and *Ntv-a* *Msh2^–/–^* mice (Figure 3, A–C) and *Ntv-a* *Msh6^+/+^*, *Ntv-a* *Msh6*^+/–^, and *Ntv-a* *Msh6*^–/–^ mice (Supplemental Figure 3A). Kaplan-Meier survival analysis revealed that both homozygous loss (MMRd) and heterozygous loss (MMRr) of *Msh2* shortened the survival of Nf1 and PDGFB mGBM–bearing mice compared with WT mice. In contrast, loss of *Msh6* produced no significant survival effect (Supplemental Figure 3B). These results aligned with the human correlative analyses in Figure 1A, suggesting a potential oncogenic role of *MSH2*, but not *MSH6*, in human GBM.

We next evaluated MMR-proficient (MMRp), MMRr, and MMRd (*Msh2*-driven) tumors for MSH2, MSH6, and MGMT expression levels using immunoblotting (Supplemental Figure 4A) and immunofluorescence (IF) staining (Supplemental Figure 4B). The results revealed a *Msh2* gene dose-dependent reduction of MSH2 and MSH6 protein levels and reduced expression of MGMT (Supplemental Figure 4A). To gain insight into the relevance of the *MSH2* dose-dependent effect on *MSH6* levels in humans, we performed Pearson correlation analysis of *MSH2* versus *MSH6* RNA expression in GBM patient samples from TCGA. Our analysis revealed a strong positive correlation between *MSH2* and *MSH6* RNA expression levels in GBM patient samples (Supplemental Figure 4C). To determine if *Msh6* gene dosage affects MSH2 levels, we generated MMRp, MMRr, and MMRd tumors through *Msh6* gene manipulation (Supplemental Figure 3B). We then assessed the expression levels of MSH2, MSH6, and MGMT using immunoblotting (Supplemental Figure 4D) and IF (Supplemental Figure 4E). The results were similar to those observed with *Msh2*-induced MMRr and MMRd tumors, except no reductions in MGMT levels were detected (Supplemental Figure 4, A and B).

We then assessed whether *Msh2*- *and Msh6*-driven MMRd tumors show increased TMB and MSI. Contrary to what has been documented in other murine tumor types (27), MMRd tumors generated by loss of either *Msh2* or *Msh6* had mutation numbers, as determined by whole-exome sequencing (WES) (single-nucleotide variant [SNV] counts in coding regions per Mb DNA) and MSI scores (28), that were comparable to the mutation numbers in MMRp tumors (Figure 3D). This raised the question of whether the increased proliferation and shortened survival of mice with MMRd tumors (compared with MMRp tumors) and/or the rapid onset of these tumors may lead to a more unified clonality of MMRd tumors, as they do not have enough time to accumulate additional mutations. To address this question, we next cultured 3 primary *Msh2*-driven MMRd and MMRp tumors in vitro for up to 9 months and then performed WES (Supplemental Figure 5A). Our results indicated that culturing MMRd and MMRp tumors for 9 months did not cause an increase in TMB or MSI (Supplemental Figure 5, B–E). This contrasts with what was shown with *Msh2* loss in lung cancer cultures from genetic models (27) and the murine breast and colon cancer cell lines 4T1 and CT26, both of which were generated by the deletion of *Msh2* using CRISPR/Cas9 technology and after serial passages of cells (29, 30). Although in human GBM the highest levels of TMB and MSI are observed in tumors harboring *MSH2* mutations, approximately 50% of *MSH2*-mutant tumors did not exhibit increased TMB (Figure 1E). It is important to note that some adult sporadic GBMs do not show apparent MSI, likely because bulk whole-genome sequencing (WGS) cannot detect it due to high clonal intratumoral heterogeneity in the setting of MMRd. However, it can be detected by single-cell WGS (4). These results indicate that loss of either *Msh2* or *Msh6* alone is probably insufficient to induce high TMB in adult glioma cells, and our models may mimic the fraction of human GBM with MMR mutations that do not exhibit increased TMB or MSI. Further studies are needed to assess whether mutation or loss of the *Pole* gene can demonstrate genetic cooperation with *Msh2* or *Msh6* in generating high TMB and MSI in GBM, similar to what was shown in endometrial cancer mouse models (31) or to examine whether the cell of origin and/or timing of tumor initiation also plays a role.

To understand what drives the shortened survival in *Msh2* loss–driven MMRd tumor–bearing mice, we performed IHC to investigate the degree of changes in MMRd and MMRr tumors and observed no alterations in the expression of OLIG2 or GFAP (Supplemental Figure 6A). However, there were increased changes in vessel area and size (CD3^+^ cells), suggesting increased angiogenesis (Supplemental Figure 6B) and increases in IBA1^+^ TAM–occupied areas (Figure 3, E and F).

### Single-cell RNA-seq indicates that loss of Msh2 increases the tumor cell fraction and contributes to an increased immune-suppressive tumor microenvironment.

Given the increased angiogenesis and IBA1 positivity observed in MMRd tumors compared with MMRp tumors, the absence of increased TMB and MSI, and the differences in survival rates of tumor-bearing mice, we next examined whether changes in the expression profiles of various cell types in MMRr and MMRd tumors contribute to the shortened survival of tumor-bearing mice. We performed single-cell RNA-seq (scRNA-seq) on 117,156 cells from 11 murine tumors (whole tumor), with 4, 4, and 3 biological replicates from *Msh2^+/+^*, *Msh2^+/–^*, and *Msh2^–/–^* mice, respectively (Figure 3G and Supplemental Table 4). We performed unsupervised clustering and systematically annotated cell clusters based on the consistent expression of known cell type markers (Figure 3H and Supplemental Figure 7A). Tumor cells were annotated according to their expression of *RCAS*, red fluorescent protein (*Rfp*), *Olig1*, *Olig2*, *Pdgfra*, and *Sox11* (Supplemental Figure 7A). We next confirmed a dose-dependent reduction of *Msh2* expression levels in tumors (Supplemental Figure 7B). *Msh2^–/–^* tumors showed increased proportions of neoplastic cells and a reduced presence of cells that constitute the tumor microenvironment (TME) (Figure 3, I and J), a finding that correlated with an increased proportion of cycling cells across all tumor cells (Figure 3K), the majority of which were double-positive for Ki67 and OLIG2 by multiplex IF staining (Figure 3L). To evaluate heterogeneity among neoplastic cells, we next scored our scRNA-seq data for the 4 human GBM cell states defined in Neftel et al. (32): oligodendrocyte precursor cell–like (OPC-like), neuron progenitor cell–like (NPC-like), astrocyte-like (AC-like), and mesenchymal-like (MES-like). Our results demonstrate that the 4 cellular states were also expressed in our murine tumors (Figure 3M) and that a dose-dependent reduction in *Msh2* had no effect on the prevalence of certain states (OPC/NPC and AC/MES) (Figure 3N).

**Figure 4:**
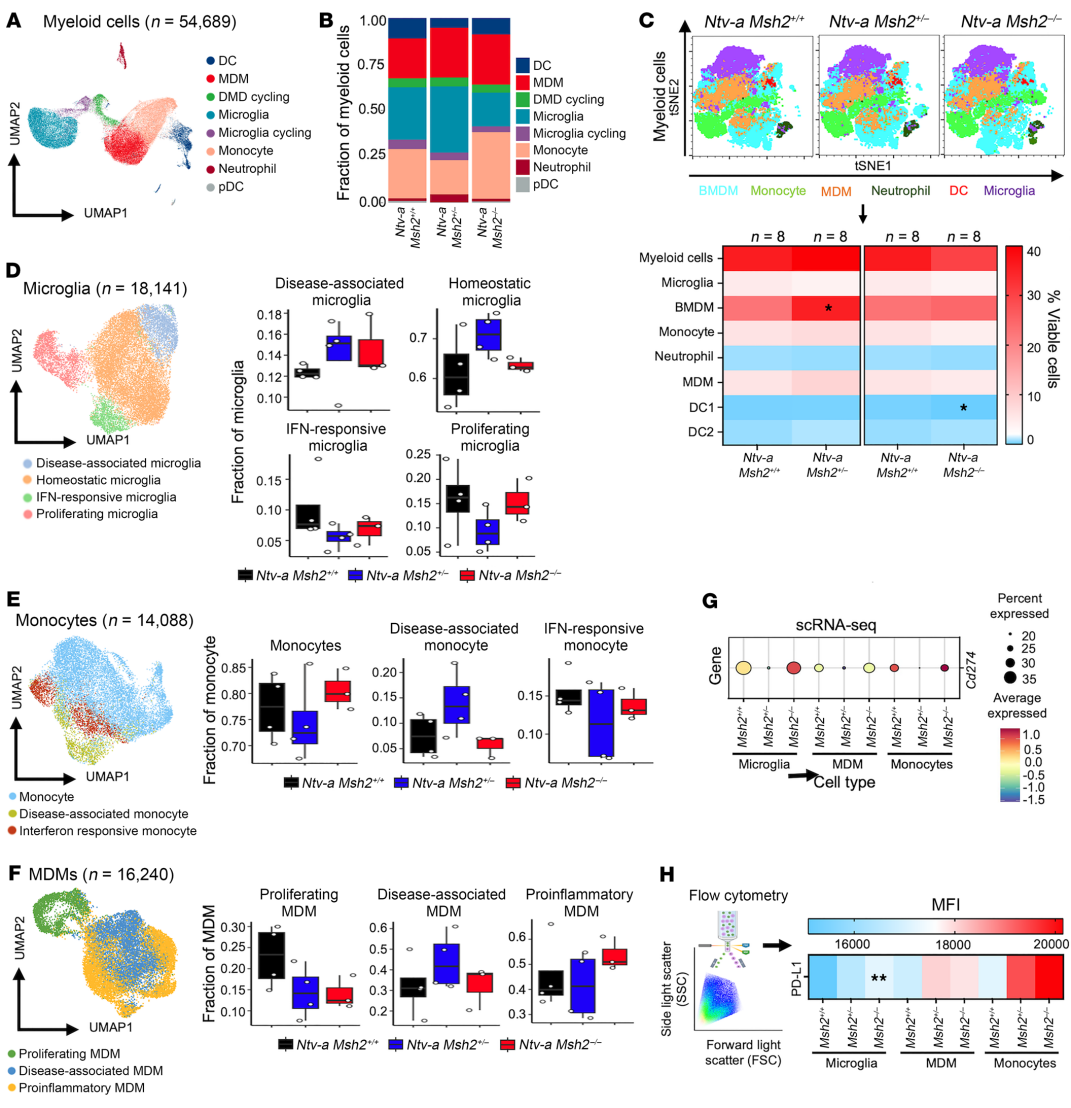
Disease-associated myeloid cell subsets are increased in *Msh2*-dependent MMRr and MMRd tumors. (**A**) UMAP plot of all sequenced mGBM myeloid cells, colored by annotated myeloid cell subset. (**B**) Stacked bar plots depicting the proportion of annotated myeloid cell subsets. (**C**) Plots of *t*-distributed stochastic neighbor embedding (*t*-SNE) showing the results of spectral flow cytometry of the myeloid cell panel in tumors (top) and heatmaps representing quantification (bottom). (**D**) UMAP plot of all mGBM microglia cells from the scRNA-seq dataset colored by annotated microglia subsets, and distribution of microglia subset proportions, split by *Msh2* status. Black = WT, blue = *Msh2* heterozygous, and red = *Msh2*-KO. (**E**) Left: UMAP plot of all mGBM monocytes colored by annotated monocytes subsets. Right: Distribution of monocyte subset proportions, split by *Msh2* status. (**F**) Left: UMAP plot of all mGBM MDMs colored by annotated MDM subsets. Right: Distribution of MDM subset proportions, split by *Msh2* status. (**G**) Dot plot showing expression levels and the percentage of cells expressing the *Cd274* gene for each annotated myeloid cell subset according to *Msh2* status. (**H**) Schematic illustration of flow cytometry and heatmap quantification of PD-L1 expression in different myeloid cell populations according to *Msh2* status. *P < 0.05 and **P < 0.01, unpaired *t* test (**C**) and 1-way ANOVA followed by Tukey’s post hoc analysis (**H**). (**D**–**F**) Box plots display the median (central line), interquartile range (box), and whiskers extending to the smallest and largest values within 1.5 times the interquartile range. Individual data points are shown as dots to provide a detailed view of the sample distribution). Panel H includes an illustration created with BioRender.

We next proceeded with the evaluation of TME constituents in our scRNA-seq dataset. Infiltrating myeloid cells and brain-resident microglia represented the largest components of the TME and accounted for approximately 46% of all sequenced cells (Figure 4A). Most of the myeloid cells were composed of microglia, monocytes, and monocyte-derived macrophages (MDMs) (Figure 4B). Next, we evaluated these results at the protein level by performing multiplex flow cytometry. We analyzed tumors at the endpoint of survival experiments using spectral flow cytometry (Figure 4C and Supplemental Figure 8). We used marker combinations and gating strategies to distinguish between various myeloid subsets based on our published protocols (33, 34). There was an increase in infiltrating bone marrow–derived myeloid cells (BMDMs) in MMRr tumors (Figure 4C), further supporting increased IBA1 staining by IHC (Figure 3E). We observed reductions in type 1 dendritic cells (DC1) in MMRd tumors compared with MMRp tumors (Figure 4C and Supplemental Figure 8).

Since we did not observe major changes in myeloid composition in MMRr or MMRd tumors, we next evaluated if the phenotypes of the major myeloid cell types were affected. To investigate microglial diversity, we categorized microglia into 4 major clusters (Figure 4D) according to the expression of key cluster genes (34) (Supplemental Figure 7C). Interestingly, we observed an increased fraction of disease-associated microglia (DAM) in both MMRr and MMRd tumors (Figure 4D). DAM are known for increased expression of immunosuppressive molecules, including *Lgals3* and *Hmox1*,and decreased expression of antigen presentation machinery and proinflammatory genes (34). Similarly, when we organized monocytes and MDMs into phenotypic clusters, we observed increased expression of disease-associated monocyte and MDM clusters in MMRr tumors (Figure 4, E and F, and Supplemental Figure 7, D and E). We previously documented that decreasing DAM clusters was associated with better survival in H3K27M-mutant diffuse midline gliomas (DMGs) (34). In human GBM, increased expression of the immune checkpoint programmed death ligand 1/PD-1 (PD-L1/PD-1) axis is associated with inferior survival rates (35). We have previously documented that PD-L1 is mainly expressed by myeloid cells in murine GBM (36). Next, we evaluated the expression levels of *Cd274* (the gene that encodes PD-L1 protein) in our scRNA-seq data (Figure 4G) and MFI of PD-L1 protein expression by flow cytometry (Figure 4H) in myeloid cell subsets in MMRp, MMRr, and MMRd tumors. Our results demonstrated increased PD-L1 expression in various myeloid fractions, especially in microglia in MMRd tumors (Figure 4H). Overall, we observed higher relative expression of immunosuppressive pathways in various myeloid cell subsets in MMRr and MMRd tumors compared with MMRp tumors. scRNA-seq data showed that all tumors, irrespective of *Msh2* gene status, had reduced *Cd80* expression, but MMRp tumors had higher expression of *Cd86* in all myeloid cell subsets — most notably in microglia. In contrast, *Cd80* expression was reduced in MMRr and MMRd tumors (Supplemental Figure 9A). These results suggest that *Msh2*-altered tumors had broader suppression of costimulatory signaling in the tumor myeloid compartment. Expression of the MHCII molecules *H2-Aa*, *H2-Ab1*, and *H2-Eb1* was high in WT monocytes and MDMs, lower in microglia, and decreased in *Msh2*-reduced tumors, suggesting reduced antigen presentation. The FACS results confirmed the scRNA-seq data showing higher expression of MHCII in monocytes and MDMs compared with microglia in WT tumors (Supplemental Figure 9B), which did not change according to the *Msh2* gene mutation status, although a trend toward reduced expression was apparent in *Msh2*-reduced tumors (Supplemental Figure 9C). IHC analysis demonstrated that areas of activated CD68^+^ TAMs, CD206^+^ immunosuppressive TAMs, and microglia within both the tumor core and invasive edge were not altered by *Msh2* gene status (Supplemental Figure 10).

**Figure 5:**
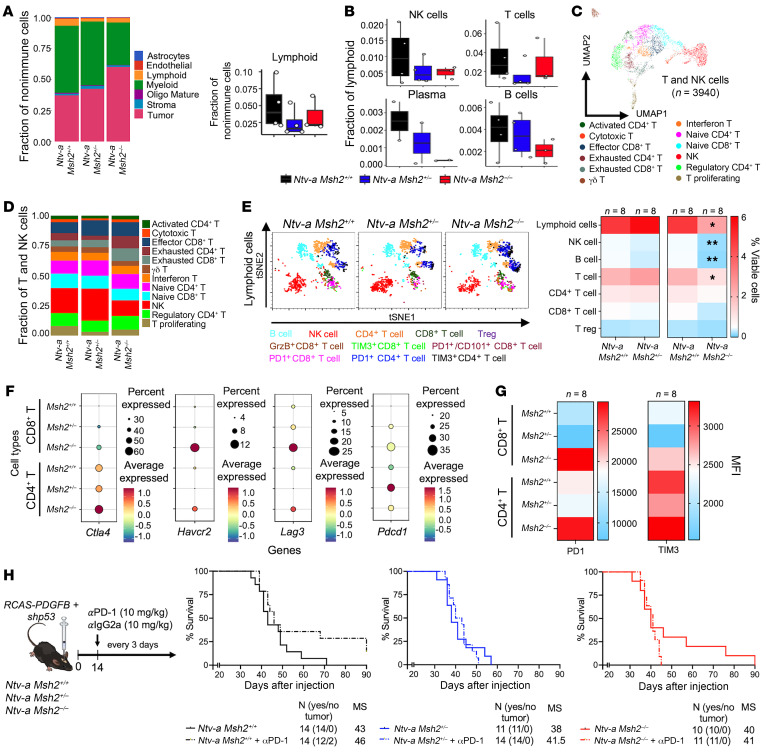
Germline *Msh2* loss–driven MMRd tumors exhibit reduced lymphoid infiltration and increased exhaustion of CD8^+^ T cells but show no response to ICI. (**A**) Left: Stacked bar plots depicting the proportion of nonimmune and immune cell subsets according to *Msh2* status. Right: Box plot showing the distribution of the proportion of the lymphoid fraction based on *Msh2* status. (**B**) Distribution of NK, T, plasma, and B cell proportions relative to all cells (see also Figure 2G). (**C**) UMAP plot of all sequenced mGBM T and NK cells, colored by cell subset annotation. (**D**) Stacked bar plots depicting the proportions of various T and NK cell subsets based on the *Msh2* status of tumors. (**E**) Schematic illustration of flow cytometry and heatmap of quantifications of lymphoid cell populations. (**F**) Dot plot showing expression levels and the percentage of cells expressing genes encoding immune checkpoint proteins across CD8^+^ and CD4^+^ T cells, categorized by *Msh2* genotype. (**G**) Heatmap quantification of PD-1 and TIM3 MFI in CD8^+^ and CD4^+^ T cells from various tumor genotypes. (**H**) Schematic illustration of the experimental steps for anti–PD-1 treatment and corresponding Kaplan-Meier survival curves for WT, *Msh2^+/–^*, and *Msh2^–/–^* mice injected with *PDGFB+shp53* treated with anti–PD-1 (αPD-1) or anti–IgG2a (αIgG2a) isotype control. *P < 0.05 and **P < 0.01, by paired or unpaired *t*-tests (**E**) and MC and GBW tests (**H**). (**A** and **B**) Box plots display the median (central line), interquartile range (box), and whiskers extending to the smallest and largest values within 1.5 times the interquartile range. Individual data points are shown as dots to provide a detailed view of the sample distribution.

To functionally characterize spatial relationships between TAMs with CD8^+^ and CD4^+^ T cells in MMRp versus MMRd tumors, we used the Orion multiplex IF platform, which is a panel of 14 antibodies specific for various cell types on 4 MMRp and 4 MMRd tumors (Supplemental Figure 11, A–G). We quantified the distance of TAMs (IBA1^+^F480^+^ double-positive) from PD-1^+^CD4^+^ T cells and PD-1^+^CD8^+^ T cells and found close colocalization to TAMs, irrespective of MMR status (Supplemental Figure 11H). To further characterize TAMs, we double-stained them with CD163 and evaluated their abundance in the tumor area and perivascular areas (marked as a 30 μm endothelial cell area) (Supplemental Figure 11I). The limited number of antibodies that can be used in spatial multiplex IF along with the low abundance of T cells — especially those coexpressing PD-1 — in 5 μm tumor sections constrained our ability to conduct detailed lymphoid phenotyping. To address this, we performed scRNA-seq and flow cytometry on whole tumors to determine how myeloid-driven immunosuppression influences the lymphoid compartment in MMRp, MMRr, and MMRd tumors. In MMRr and MMRd tumors, we observed a lower relative abundance of the total lymphoid fraction (Figure 5A), including NK cells, T cells, plasma, and B cells compared with MMRp tumors (Figure 5B and Supplemental Figure 12A). We then separated B cells and plasma cells and further delineated T cell subsets according to lineage and functional markers (Supplemental Figure 12A and Figure 5, C and D). We observed a relatively lower abundance of naive CD8^+^ T cells and proliferating T cells in MMRr and MMRd tumors (Supplemental Figure 12A).Next, we used multicolor flow cytometry with a larger number of samples to determine whether our scRNA-seq observations were similarly valid at the protein level (Figure 5E and Supplemental Figure 12B). We used marker combinations and gating strategies to distinguish between various lymphoid subsets based on our previously published protocols (33, 34). Flow cytometric data, in conjunction with scRNA-seq analysis, revealed decreases in total lymphoid cells, B cells, NK cells, and T cells in MMRd tumors compared with MMRp tumors. These effects were not observed in MMRr tumors (Figure 5E and Supplemental Figure 12B). Since we observed a higher relative abundance of disease-associated clusters in various myeloid cell subsets and elevated expression of PD-L1 is associated with increased exhaustion of T cells, we evaluated the expression of various genes encoding immune checkpoint molecules on CD4^+^ and CD8^+^ T cells, including cytotoxic T lymphocyte antigen 4 (CTLA4; *Ctla4*), T cell immunoglobulin and mucin protein 3 (TIM3; *Havcr2*), lymphocyte activation gene 3 (LAG-3; *Lag3*), and PD-1 (*Pdcd1*) in MMRp, MMRr and MMRd tumors. We observed higher relative expression of all immune checkpoint molecules in CD8^+^ T cells in MMRd tumors relative to MMRr and MMRp tumors (Figure 5F), which we further confirmed by flow cytometry for PD-1 and TIM3 (Figure 5G). PD-L1 expression was low in myeloid cells of MMRp tumors (Figure 4G), similar to what was shown in human GBM (35) and in contrast to brain metastasis (37), which has been suggested to be a contributor to the lack of association seen between PD-L1 expression in tumors and survival in clinical trials evaluating ICI for GBM (38). To functionally characterize CD8^+^ T cells, we conducted T cell–tumor cell coculture experiments to first evaluate the effect of *Msh2* heterozygosity on stimulated CD8^+^ T cell proliferation and effector cytokine production, and then to assess the effects of coculturing MMRp and MMRd primary GBM stem-like cells (GSCs) on these functions (Supplemental Figure 13A). Our results indicated that, while proliferation was not affected, effector cytokine production was reduced in stimulated CD8^+^ T cells from *Msh2*-heterozygous mice, suggesting reduced activation or increased exhaustion. Furthermore, coculturing of stimulated WT and *Msh2* heterozygous CD8^+^ T cells with *MMRp* and *MMRd* GSCs markedly reduced the production of effector cytokines, including granzyme B and IFN-γ, and only MMRd GSCs were able to diminish TNF-α levels. These findings indicate that CD8^+^ T cell proliferation was unaffected by *Msh2* reduction, whereas effector cytokine production was reduced and was even further reduced by coculturing with tumor cells, particularly MMRd GSCs (Supplemental Figure 13B). These results show that heterozygous loss of *Msh2* dampened CD8^+^ T cell effector function without affecting proliferation and was exacerbated by coculturing with MMRd GSCs.

In MMRd tumors, we observed increased expression of both PD-L1 and PD-1; we therefore asked whether these tumors would respond to anti–PD-1 therapy. We performed an experiment to evaluate the effects of anti–PD-1 antibodies in MMRp, MMRr, and MMRd tumor–bearing mice. We started treatment at an earlier stage of tumor development and continued until the mice reached the humane endpoint, as illustrated in Figure 5H. Kaplan-Meier survival curves showed no differences in survival of MMRp, MMRr, and MMRd tumor–bearing mice treated with isotype control or anti–PD-1 antibodies (Figure 5H). Our results indicate that MMRd tumors contained a lower percentage of lymphoid cells and had an increased number of tumor cells with higher proliferation rates, raising questions about how much each of these factors contributes to shortened survival. To determine whether lymphoid fraction reduction contributes to the shortened survival of mice bearing *Msh2* loss–induced MMRd tumors, we evaluated lymphoid recruitment in both MMRr and MMRd tumors induced by *Msh6* loss, which did not result in survival differences compared with *Msh6* WT GBM–bearing mice. Our findings show that the survival of tumor-bearing mice was not affected by either MMRr or MMRd tumors induced by *Msh6* (Supplemental Figure 3). Flow cytometry of lymphoid subsets showed no changes in lymphoid, NK cells, B cells, or T cells in *Msh6*-induced MMRd tumors, in contrast to what we observed in *Msh2*-induced MMRd tumors (Supplemental Figure 12C). Overall, these results suggest that reduced lymphoid recruitment in *Msh2*-deficient tumors may contribute to tumor aggressiveness.

### Somatic MMRd tumors do not shorten survival of mice with GBM.

**Figure 6:**
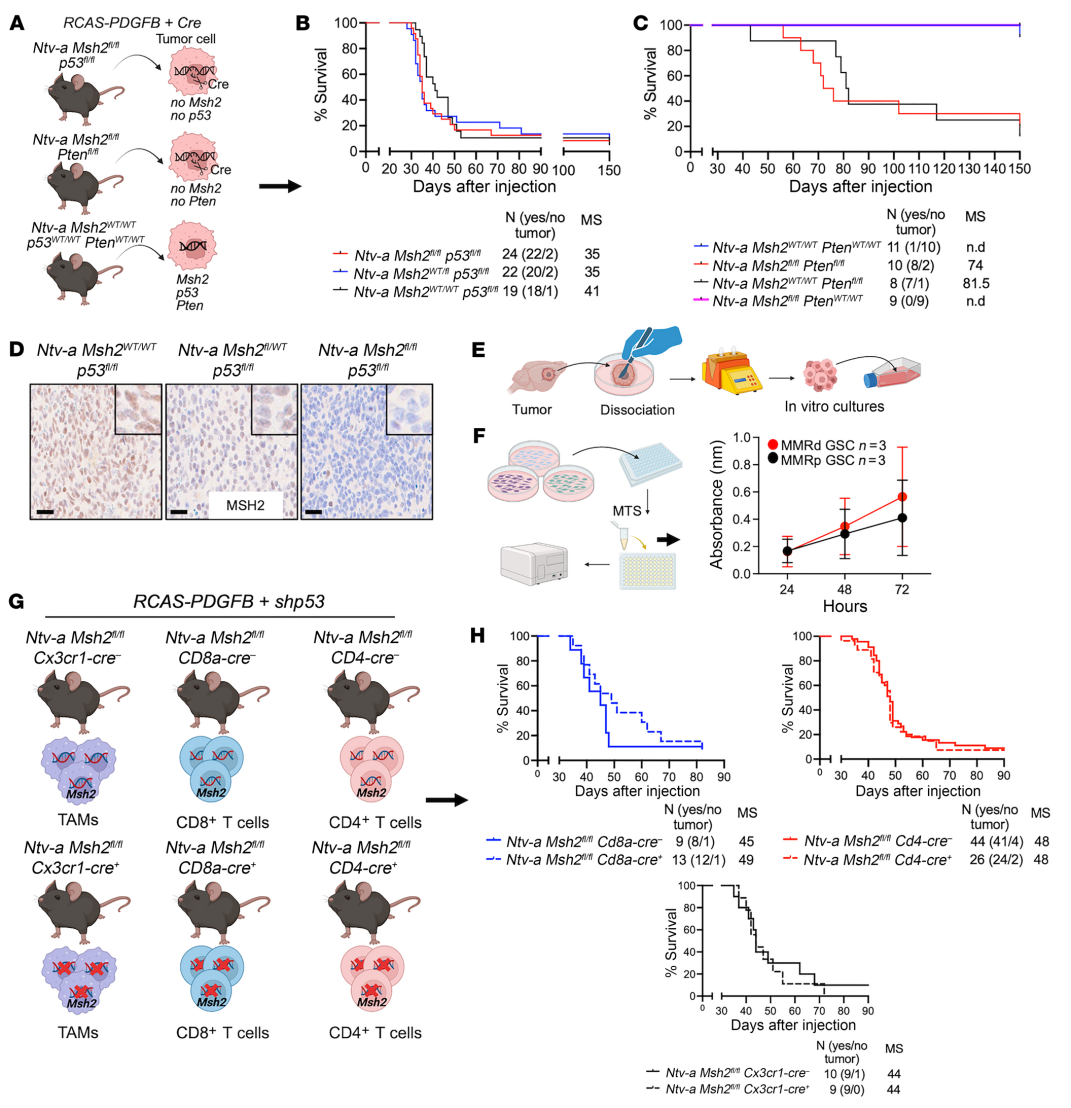
Somatic MMRd does not lead to shortened survival in tumor-bearing mice. (**A**) Schematic illustration for the generation of adult somatic *Msh2* loss–induced MMRd GBM models utilizing PDGFB overexpression along with either *shp53 or shPten* across various genotypes. (**B** and **C**) Kaplan-Meier survival curves of tumors from mice of various genotypes. (**D**) Representative images and IHC quantification for MSH2 in somatic homozygous or heterozygous MMRd tumors. Scale bars: 100 μm and 50 μm (insets). (**E**) Illustration of the isolation and culturing of freshly dissociated primary tumors generated in germline *Msh2*, WT, and *Msh2*-deficient mice. (**F**) Illustration of the experimental setup and results for the MTS assay for MMRp and MMRd primary tumor cell cultures maintained in GSC conditions. The experiments included 3 replicates for each genotype, with primary cell lines derived from 3 different tumor-bearing mice. Experiments were repeated at least 3 times for each line at 24 hours, 48 hours, and 72 hours. MTS, 3-(4,5-dimethylthiazol-2-yl)-5-(3-carboxymethoxyphenyl)-2-(4-sulfophenyl)-2H-tetrazolium. Data are presented as the mean ± SD.(**G**) Schematic illustration showing the generation of adult *Msh2* cell-specific depletion in GBM models utilizing PDGFB overexpression combined with *shp53* across different genotypes. (**H**) Kaplan-Meier survival curves for tumor-bearing mice of various genotypes. The experiments included 3 replicates for each genotype, with primary cell lines derived from 3 different tumor-bearing mice. Experiments were repeated at least 3 times for each line at 24 hours, 48 hours, and 72 hours. MC and GBW test (**B**, **C**, and **H**). Panels **A** and **E**–**G** include illustrations created with BioRender.

To investigate the role of tumor cells in the aggressiveness of MMRd tumors in the context of germline mutation (in all cells), we developed somatic MMR-deficient models (MMRd only in tumor cells). We adopted our autochthonous *Ntv-a* mouse model of GBM by breeding into conditional *Msh2*-KO (39) and *p53*-KO (40) alleles (*Ntv-a* *Msh2^fl/fl^* *p53^fl/fl^* and *Ntv-a* *Msh2^fl/WT^* *p53^fl/fl^* mice). Intracranial delivery of RCAS-PDGFB and RCAS-Cre successfully created *Msh2* (reduction or loss) and *Tp53* loss only in tumor cells (Figure 6A). Kaplan-Meier survival analysis showed that the presence of somatic MMRr or MMRd did not affect the survival of tumor-bearing mice compared with WT control mice (Figure 6B), in contrast to germline MMRr or MMRd tumor–bearing mice, which had shortened survival (Figure 3, B and C). Furthermore, there were no differences in tumor grade distribution between MMRp and somatic MMRr or MMRd tumors (Supplemental Figure 14A). Combined loss of *Msh2* and *p53* without PDGFB was not sufficient to induce tumors (Supplemental Figure 12D). To complement our findings, we created a somatic MMRd model in which *Pten* was codeleted, thus generating a *Ntv-a* *Msh2^fl/fl^* *Pten^fl/fl^* cross. Intracranial delivery of RCAS-PDGFB and RCAS-Cre successfully created tumors with *Msh2* (reduction or loss) and *Pten* deficiency only in tumor cells. Kaplan-Meier survival analysis showed that the presence of somatic MMRr or MMRd did not affect the survival of tumor-bearing mice compared with WT controls (Figure 6C). IHC staining from somatic MMRr and MMRd tumors showed a dose-dependent decrease in *MSH2* levels in tumor cells (Figure 6D). However, no changes were detected in the proportion of different cell types (TAMs, microglia, and neutrophils) or vessel area and size (CD31^+^ cells) within the TME (Supplemental Figure 14B) among these genotypes. In addition, no differences in proliferation were observed between MMRp and MMRd tumors, as assessed by pH3 staining (Supplemental Figure 14B), consistent with the Kaplan-Meier survival results (Figure 6B). We created primary GSC cultures from MMRp and MMRd tumors and assessed their growth using the 3-(4,5-dimethylthiazol-2-yl)-5-(3-carboxymethoxyphenyl)-2-(4-sulfophenyl)-2H-tetrazolium (MTS) colorimetric cell growth assay (Figure 6E). Similar to the in vivo survival curves (Figure 6B), the results showed no differences in the growth of MMRp and MMRd primary tumor cultures (Figure 6F). We next generated a series of crosses to allow specific deletion of *Msh2* in CX3CR1^+^ myeloid cells (monocytes, MDMs, and microglia), CD4^+^ and CD8^+^ T cells (Figure 6G). Kaplan-Meier survival curves indicated no differences in survival, although a trend was observed only in mice with *Msh2*-deficient CD8^+^ T cells (Figure 6H). These results suggest that the reduced survival of mice with germline MMR deficiency was due to the combined effects of an altered TME and tumors cells generated by *Msh2* loss.

### MMRr and MMRd confer resistance to TMZ in GBM–bearing mice.

Silencing MGMT has been shown to be a predictive biomarker of response to TMZ in GBM (3). When the MGMT promoter is methylated, it results in RNA expression silencing, correlating with reduced or absent protein levels (41) and leading to enhancement of TMZ’s antitumor toxicity. The tumors in our study exhibited varying levels of MGMT expression (Supplemental Figure 4, A and D). To assess *Mgmt* expression levels, we performed quantitative real-time reverse transcription PCR (qRT-PCR) in tumors and naive brains, tumor samples (tumor plus TME), and freshly sorted RFP^+^ tumor cells. Our results showed no differences in *Mgmt* expression levels in naive or tumor brains and reduced expression of *Mgmt* in freshly sorted tumor cells (Supplemental Figure 15A). We next performed immunoblotting to assess MGMT protein levels in presorted (single-cell suspensions from fresh tumors), RFP^+^ (sorted tumor cells), and RFP^–^ cells (TME cells), which revealed variable expression levels of MGMT, but higher expression in presorted and RFP^–^ cells compared with RFP^+^ tumor cells. We then sorted tumor cells into RFP-high and RFP-low groups and immunoblotted for MGMT. We found that MGMT expression did not correlate with RFP intensity and was lower than in the presorted cells (Supplemental Figure 15B). This indicates that the GBMs contained varying proportions of tumor cells expressing varying levels of MGMT. This was also evident from immunoblots of fresh tumors showing various levels of MGMT expression (Supplemental Figure 4, A and D). Cultured primary cells from these tumors showed no MGMT expression (Supplemental Figure 15C), which might be explained by culture conditions that were selecting for survival of MGMT-low or -nonexpressing cells or because MGMT expression was being silenced in the cultures. Meanwhile, MSH2 and MSH6 expression levels were not affected by the culture conditions (Supplemental Figure 15C). To check if the loss of MGMT expression in our primary tumor cell cultures was unique, we performed immunoblotting for MGMT in various established murine cancer cell lines, all of which showed no MGMT expression (Supplemental Figure 15D). On the basis of our results, we hypothesized that tumors in vivo contain a mix of tumor cells with high and low expression of MGMT and that treatment with TMZ will have antitumor efficacy by targeting MGMT-low/-silenced cells, establishing our models as valuable tools for studying TMZ resistance driven by MMRd.

**Figure 7:**
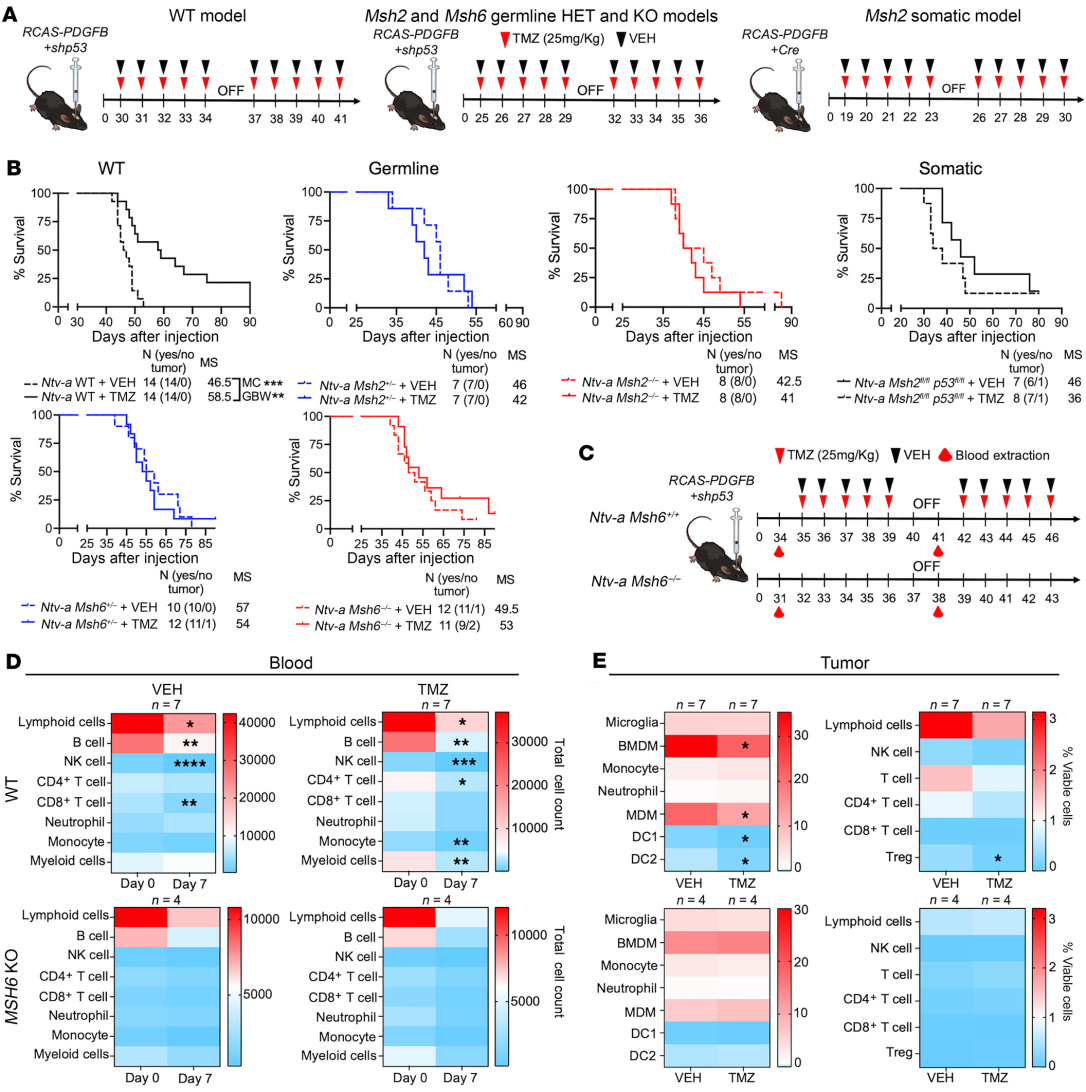
Germline MMRr, MMRd, and somatic MMRd confer resistance to TMZ in vivo. (**A**) Schematic illustration of the experimental steps for TMZ treatment. (**B**) Survival curves of tumor-bearing mice treated with VEH or TMZ. (**C**) Schematic illustration of the experimental steps for TMZ treatment and the time points for blood and tumor collection. (**D** and **E**) Heatmap quantifications of spectral flow cytometry myeloid and lymphoid panels in the blood at days 0 and 7 (**D**) and tumors at the survival endpoint (**E**). *P < 0.05, **P < 0.01, ***P < 0.001, and ****P < 0.0001, by MC and GBW test (**B**), paired *t* test (**D**), and unpaired *t* test (**E**).

To determine whether MMRr tumors behave similarly to MMRd tumors, we created germline MMRp, MMRr, and MMRd PDGFB mGBMs. On the basis of the median survival time for each group, we then treated tumor-bearing mice with 2 cycles of clinically relevant doses of TMZ 25 days (for MMRr and MMRd tumors) and 30 days (for MMRp tumors) after injection (Figure 7A). Our results indicated an increase in the survival time for MMRp tumor–bearing mice compared with the vehicle-treated (VEH-treated) group, but not for MMRr or MMRd mice (Figure 7B). In the VEH-treated group, we observed a reduction in total lymphoid cells, CD8^+^ T cells, B cells, and NK cells at day 7 after treatment (Figure 7, C and D). This reduction was likely due to an increased tumor burden in mice and their reaching the survival endpoint, as we have previously demonstrated (42). We analyzed immune cell populations from the blood of MMRp tumor–bearing mice at day 7 after treatment and found that TMZ treatment resulted in a reduction of CD4^+^ T cells, total myeloid cells, and monocytes compared with pretreatment cell numbers (Figure 7, C and D, upper panel). However, these differences were not observed in the blood before or after TMZ treatment in either *Msh2*- or *Msh6*-induced MMRr and MMRd tumor–bearing mice (Figure 7D, lower panel). The reductions in these cell populations that have been shown to exhibit protumorigenic function could have been contributing to the survival benefit seen in MMRp tumor–bearing mice treated with TMZ. When we compared the TME of MMRp tumors at the survival endpoint, we observed a decrease in total BMDMs, MDMs, Tregs, DC1, and type 2 dendritic cells in the TMZ-treated group compared with the VEH-treated group. No differences were observed in MMRr or MMRd tumors treated with TMZ (Figure 7E). Overall, these results demonstrate that TMZ treatment had antitumor efficacy in MMRp tumor–bearing mice, but not in *Msh2*- or *Msh6*-driven MMRr or MMRd tumor–bearing mice.

### KL-50 treatment improves survival in somatic and germline MMRd tumor–bearing mice.

**Figure 8:**
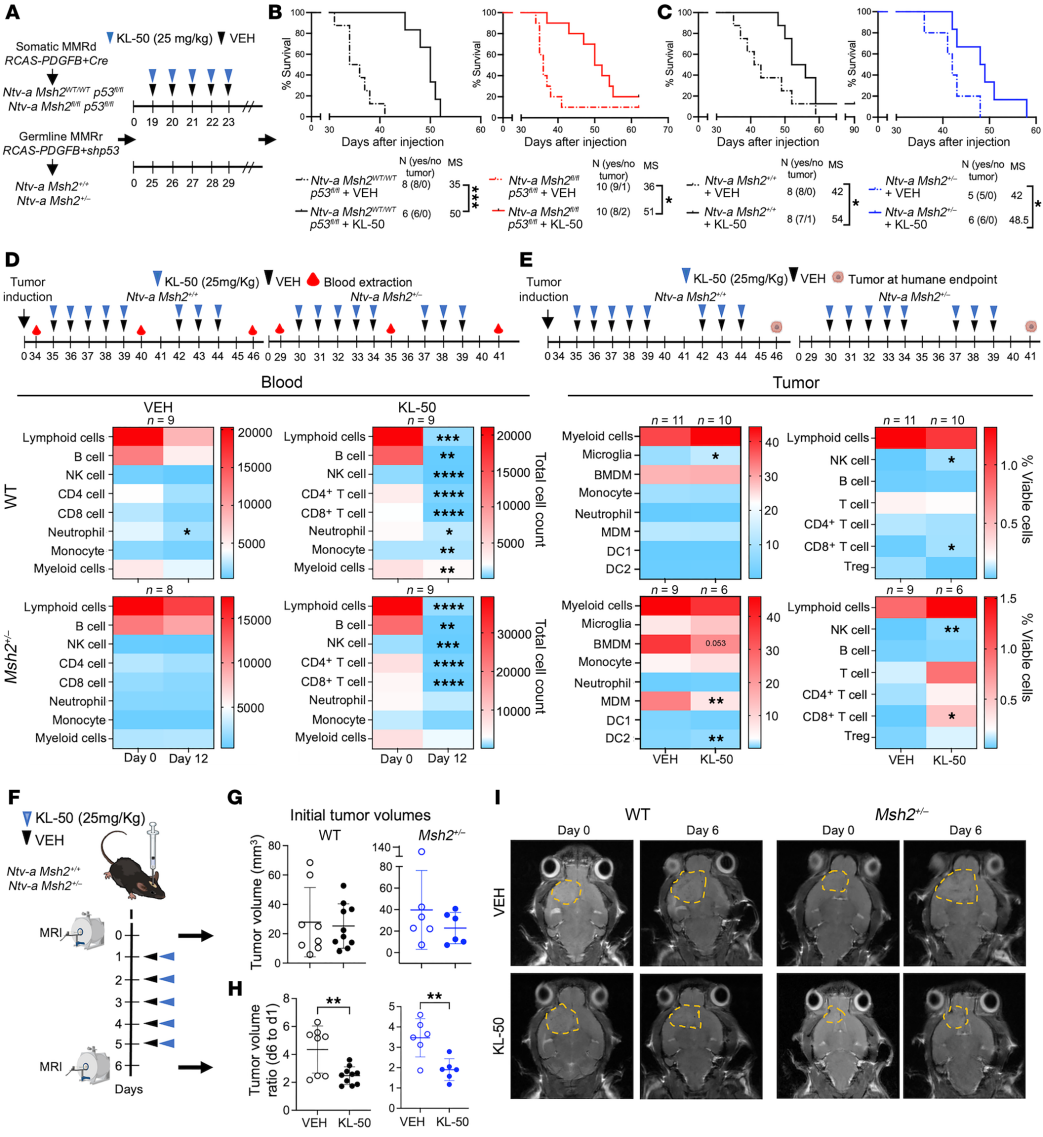
KL-50 treatment is potent against MMRp tumors as well as germline and somatic MMRd tumors. (**A**) Schematic illustration of experimental steps for KL-50 treatment in WT and somatic MMRd tumor–bearing mice and in WT and germline MMRd tumor–bearing mice. (**B**) Survival curves for WT and somatic MMRd tumor–bearing mice treated with VEH or KL-50. (C) Survival curves for WT and *Msh2* heterozygous tumor–bearing mice treated with VEH or KL-50. (**D**) Schematic illustration of experimental steps for KL-50 treatment and time points of blood and heatmap quantifications of spectral flow cytometry myeloid and lymphoid panels in the blood at treatment days 0 and 12 and tumors at day 12 (48 hours after the last dose). (**E**) Schematic illustration of the experimental steps for KL-50 treatment and the time point of tumor collection, along with heatmap quantifications of spectral flow cytometry myeloid and lymphoid panels. (**F**) Schematic illustration of the experimental steps for MRI-based assessment of KL-50 efficacy in germline MMRd tumor–bearing mice. Illustration created with BioRender. (**G**) Initial MRI volumes were equally distributed in the VEH and KL-50 treatment groups in WT and germline MMRd tumors. (**H**) Ratio of tumor volume on day 6 after treatment over initial pretreatment volumes. (**I**) Representative MRI images of WT and germline MMRd tumor–bearing mice at day 0 and day 6 after VEH or KL-50 treatment. *P < 0.05, **P < 0.01, ***P < 0.001, and ****P < 0.0001, by MC and GBW test (**B**) and paired t-test (**D)** and unpaired t-test (**E** and **H**). Data are presented as the mean ± standard deviation.

Our results indicate that MMRp tumors contained cells with varying levels of MGMT expression. Treatment with TMZ extended the survival of MMRp tumor–bearing mice in contrast to mice bearing MMRr or MMRd tumors. Next, we aimed to assess the effectiveness of KL-50 treatment and fully characterize the response of MMRp, MMRr, and MMRd tumors, as well as its effect on the immune TME and blood profiles of tumor-bearing mice. KL-50 is a imidazotetrazine-based agent that induces DNA interstrand crosslinks (ICLs) and subsequent double-stranded breaks in an MMR pathway–independent manner. KL-50 is designed to transfer a 2-fluoroethyl substituent to *O*^6^-G, thereby generating *O*^6^-(2-fluoroethyl) guanine (*O*^6^-FEtG) that can be directly repaired by MGMT. In the absence of MGMT, *O*^6^-FEtG undergoes hydrolytic rearrangement, resulting in the formation of G–C interstrand crosslinks and cytotoxicity that does not rely on “futile cycling” through the MMR pathway (18, 19, 43). While this mechanism is similar to that of lomustine, which is commonly used to treat TMZ-resistant GBM, the kinetics of ICL formation are slower with KL-50, allowing MGMT-proficient healthy cells to reverse DNA alkylation (43). We created somatic MMRp and MMRd tumors by injecting PDGFB and Cre into *Ntv-a Msh2^WT/WT^* *p53^fl/fl^* and *Ntv-a* *Msh2^fl/fl^* *p53^fl/fl^* mice. Nineteen days after the injections, we began KL-50 or VEH treatment, following the schematic illustration in Figure 8A. KL-50 treatment prolonged the survival of both MMRp and MMRd tumor–bearing mice compared with the VEH-treated groups (Figure 8B). We then proceeded to test the effectiveness of KL-50 in tumor-bearing mice with MMRp and germline MMRr tumor–bearing mice. Twenty-five days after injection, we initiated a cycle of KL-50, following the schematic illustration in Figure 8A. KL-50 treatment extended the survival of MMRp and germline MMRr tumor–bearing mice compared with the VEH-treated mice (Figure 8C).

To better understand and compare the effects of KL-50 with TMZ (Figure 7), we treated mice bearing germline MMRp or MMRr tumors with KL-50 or VEH, as shown in Figure 8D. We observed a transient reduction of various immune cell subsets in the blood of VEH-treated mice 12 days after treatment. There were no changes observed in the blood of MMRr tumor–bearing mice treated with VEH (Figure 8D). In contrast to the VEH-treated animals, the blood of KL-50–treated MMRp tumor–bearing mice at day 12 after treatment showed severe reductions in nearly all immune cell types assessed. We noted reductions in myeloid cells, B cells, NK, and total myeloid cells, with no changes in CD4^+^ or CD8^+^ T cells (Figure 8F). At post-treatment day 12, analysis of MMRp tumors revealed increases in microglia, CD8^+^ T cells, and NK cells. On the other hand, MMRr tumors showed decreases in BMDMs and MDMs, along with increases in CD8^+^ T and NK cells (Figure 8E). To assess the immediate effect of KL-50 on tumors, we conducted an MRI-guided experiment, following the steps illustrated in Figure 8F. We performed T2-weighted MRI on mice bearing MMRp or MMRr tumors to ensure that both the treatment and VEH groups enrolled sizable tumors with similar volumes and sexes equally distributed in both groups to ensure that samples from both groups included larger-sized tumors of comparable volume and equal numbers of male and female mice (Figure 8G). Mice were treated with KL-50 at 25 mg/kg or with VEH for 5 consecutive days and subjected to post-treatment MRI. Our data indicate that KL-50 treatment led to a reduction in tumor growth in both MMRp tumor–bearing mice and MMRr tumor–bearing mice (Figure 8, H and I). An increase in cell death was observed by TUNEL staining only in MMRr tumors (Supplemental Figure 16). The combined effects of reducing immunosuppressive myeloid cell subsets and increasing CD8^+^ T and NK cell populations in tumors could also have contributed to the efficacy of KL-50. These results demonstrate the antitumor efficacy of KL-50 in MMRp and somatic and germline MMRr and MMRd tumor–bearing mice, in contrast to TMZ, which only showed antitumor efficacy in MMRp tumors.

## Discussion

MMR gene mutations are rare in primary GBMs. However, their frequency increases with TMZ treatment in recurrent GBMs and co-occurs with increased TMB (4). This raises the question of whether MMR mutations drive TMB formation de novo or result from TMZ exposure. Prior GBM studies have modeled MMR loss by inducing MSH6 loss through prolonged TMZ exposure (which co-occurs with HM) (44), CRISPR-mediated deletions of *MSH6* (45), or shRNA silencing of *MSH2* (19). These studies focused on evaluating the mechanisms of resistance to TMZ and explored alternative compounds, but they did not establish the causal role of MMR loss in HM.

To address this knowledge gap, we developed GEMMs of MMRd and MMRr HGGs using germline or conditional deletion of *Msh2* or *Msh6* in RCAS/tv-a–based glioma models, which faithfully mimic HGG initiation and progression in both neonatal and adult mice (23, 36). In pediatric models, loss of *Msh2*, but not *Msh6*, drives the transition from a low- to high-grade glioma, whereas loss of either gene further accelerates de novo HGG progression. In adult GBM models, accelerated tumor growth and reduced survival are observed only when MMRr and MMRd is driven by *Msh2* loss, indicating that *Msh2* is a more potent oncogenic driver, whereas the effect of *Msh6* is context dependent.

Germline MMRr and MMRd tumors exhibit MSI and TMB levels similar to those of MMRp GBMs, suggesting that MMRd alone does not drive HM in this context. This likely results from the timing of tumor initiation, the cell of origin, and the rapid tumor onset driven by oncogenic PDGFB signaling coupled with loss of *Tp53* or *Pten*. In contrast, *Msh2* loss in autochthonous *KRAS^G12D^* and *Tp53*-deficient murine lung tumors resulted in an increased TMB, an effect further enhanced by in vitro passaging of tumor cells (27). GBMs diverged from this pattern, as primary MMRd GBM cultures maintained stable TMB and MSI, indicating that *Msh2* loss alone was insufficient to induce HM in glioma cells. Analysis of a limited number of GBM patient samples from TCGA indicated that single MMR gene mutations only increase the TMB and MSI when concurrent with *POLE* mutations. These observations suggest that *POLE* mutation may cooperate with MMR gene mutations to promote HM phenotype and underscore the need for further investigation.

We show that the shortened survival times of mice bearing germline *Msh2*-driven MMRr or MMRd tumors were associated with increased angiogenesis and TAM coverage, increased BMDM infiltration, and reduced lymphoid cell recruitment. scRNA-seq results revealed increased disease-associated myeloid cell subsets, increased expression of PD-L1, which correlated with a reduced lymphoid cell population, and increased expression of immune checkpoint molecules, including PD-1, in CD8^+^ T cells, suggesting increased exhaustion. Yet, treatment with anti–PD-1 therapy did not result in improved survival of mice with MMRd or MMRr tumors, which showed treatment resistance similar to that seen in mice bearing MMRp tumors. It is plausible that the loss of MMR genes led to an increase in immune exhaustion and expression of immune checkpoint molecules; however, without increases in the TMB and MSI, this loss failed to elicit an effective response to ICI. Clinical data show that ultra-HM MMRD and DNA polymerase pHGGs are characterized by increased PD-L1 expression, which is associated with improved overall survival (OS) in response to ICI (16). Survival rates of mice with somatic MMRd, MMRr, or MMRp tumors were comparable in vivo, consistent with similar growth rates of MMRd and MMRp tumor cells in vitro. These results indicate that the reduced survival of mice bearing germline MMRd or MMRr tumors was probably caused by a combination of *Msh2* loss in tumor cells and in the TME, especially CD8^+^ T cells. Supporting this notion, *Msh2* heterozygous loss dampened CD8^+^ T cell effector function without affecting proliferation, which was further enhanced by coculturing with MMRd GSCs.

MMRd induced by TMZ in a subset of TMZ-sensitive cells has been documented in gliomas (4), and even a slight reduction in *MSH2* levels can confer TMZ resistance in GBMs (46). These findings show that changes in the expression levels of MMR genes, and not just mutations, can affect some of their biological functions. Here, we show that MMRr was sufficient to confer resistance to TMZ in TMZ-sensitive tumors, as with MMRd. TMZ treatment reduced the numbers of circulating and intratumoral myeloid cells, including BMDMs and Tregs, cell populations known to promote GBM growth and immune suppression (47). Severe myelosuppression has been documented in a subset of patients with GBM treated with TMZ (48), and lower baseline neutrophil counts correlate with improved outcomes in patients with newly diagnosed GBM, independent of steroid use (49). We observed reduced lymphoid populations in MMRp tumor–bearing mice, consistent with TMZ-induced lymphopenia reported as a poor prognostic factor in GBMs (50). Collectively, these findings suggest that TMZ may exert dual effects in the TME in MMRp tumors, simultaneously reducing immunosuppressive cells in tumors while impairing the peripheral immune response, similar to what has been shown in human GBMs (51).

KL-50 was developed to retain TMZ efficacy in MGMT-silenced cells while circumventing the reliance on the MMR pathway for its antitumor efficacy (19). In contrast to TMZ, KL-50 treatment had comparable antitumor efficacy in both somatic MMRp and MMRd tumor–bearing mice, including germline MMRr tumor–bearing mice. Unlike TMZ, KL-50 induced a reduction in microglia, in addition to the reduction in NK and CD8^+^ T cells in tumors. In MMRr tumors, KL-50 treatment reduced intertumoral BMDMs, MDMs, NK cells, and CD8^+^ T cells. In both MMRp and MMRr tumor–bearing mice, treatment with KL-50 resulted in increased cell death, as shown by IHC, and reduced tumor growth, as determined by MRI. These results suggest that KL-50 can be an alternative therapy for TMZ, not only for TMZ-induced MMRd tumors, but also for patients with de novo pathways with inherited defects in *POLE* and MMR genes. These models can be used to investigate further mechanisms of resistance to TMZ in both primary and recurrent tumors, as well as to explore the biological functions of other MMR genes. Importantly, these models can also be used to test whether mutations in multiple MMR genes, alone or combined with *Pole/d* mutations, can create models with MSI and a high TMB and determine whether this is cell-of-origin and age dependent. And, finally, these models can be used to determine if there is a causal relationship between hereditary MMRd and POLE loss and the response to immune checkpoint blockade, along with the underlying mechanisms driving this relationship.

## Methods

Detailed methods can be found in Supplemental Methods.

### Sex as a biological variable.

Our study examined male and female animals, and similar findings are reported for both sexes.

### Mice, virus generation, and tumor induction.

Adult mice of both sexes (equal distribution in various groups) in the 8- to 16-week age range and newborn P0–P3 mice were used for experiments, and tumors were generated according to previously published protocols (23, 42). Specific details regarding various mice used can be found in the Supplemental Methods.

### Spectral flow cytometry and flow sorting.

Single-cell suspensions from tumors were generated as previously described (42). Cells were stained with primary antibodies (Supplemental Table 1). All data were collected on a Cytek Aurora spectral flow cytometer. Data were analyzed using FlowJo 10 software (BD Bioscience) according to our previously published protocols (42).

### In vivo survival experiments with anti–PD-1, TMZ, and KL-50 treatments.

Anti–PD-1 (10 mg/kg body weight, Bio X Cell, BE0146) or isotype control aIgG2a antibody (10 mg/kg body weight, Bio X Cell, BE0089) was administered i.p. every 3 days until the humane endpoint. TMZ (25 mg/kg body weight, MilliporeSigma, T2577) or vehicle control (10 μL/g body weight of 0.9% NaCl containing 10% DMSO; McKesson, 2718344) was administered orally on a schedule of 5 days on and 2 days off, followed by another 5 days on (total of 10 doses). KL50 (25 mg/kg body weight) or vehicle control (10% [2hydroxypropyl]-βcyclodextrin; MilliporeSigma, 332607) was administered once daily for 5 consecutive days.

### MRI image acquisition.

T2-weighted MRI scans of tumor-bearing mice were performed as described previously (52).

### scRNA-seq and data analysis.

scRNA-seq using the Chromium 3′ V3 platform (10x Genomics) was performed as previously described (34), and data analysis was done following the pipeline of Ross et al. (2024) (34). The sample manifest for scRNA-seq is provided in Supplemental Table 4.

### IHC and IF.

All IHC staining was performed on a Bond Rx (Leica Biosystems), and the list of antibodies for IHC and IF are included in Supplemental Table 1 and for multiplex IF in Supplemental Table 2.

### RNA extraction and qRT-PCR.

RNA extraction and qRT-PCRs were performed according to published protocols (34 and 42), and primers are listed in Supplemental Table 3.

### GENIE data analysis.

We downloaded the AACR Project GENIE version 17.0 dataset from Synapse (21). Details on the analysis are provided in the Supplemental Methods.

### Statistics.

Graphs were created using GraphPad Prism 8 (GraphPad Software) or R. Variables from 2 experimental groups were analyzed using unpaired or paired parametric 2-tailed *t* tests as appropriate, assuming equal SDs. One-way ANOVA was used to compare multiple variables for more than 2 groups; details for each test are provided in the figure legends. Kaplan-Meier survival analysis was performed using the log-rank Mantel-Cox (MC) test and the Gehan-Breslow-Wilcoxon (GBW) test. Data are presented as the mean ± SD. Numbers of samples in each group are indicated in the individual figures.

### Study approval.

All experimental procedures were approved by the IACUCs of Mount Sinai School of Medicine (protocol nos. 2019-00619 and 2014-0229) and the Perelman School of Medicine, University of Pennsylvania (protocol no. 807737).

### Data availability.

All scRNA-seq raw data are deposited in the Gene Expression Omnibus (GEO) database (GSE292092). Values for all data points shown in the graphs are provided in the Supporting Data Values file. Further information and requests for resources and reagents should be directed to and will be fulfilled by the corresponding author.

## Author contributions

All authors contributed to the design of the studies and to the writing and editing of the manuscript. MPV, TJ, KR, AB, G Piñero, G Price, MD, DB, RR, and DH conducted experiments. MPV, TJ, KR, AB, G Piñero, G Price, NS SF, AA, SWC, R Roche, ER, JCV, NMT, and AMT analyzed data. R Rabadan, AMB, RMS, JCV, AMT, ZC, SWC, R Rabadan, WE, TER, SF, RSB, and DH provided intellectual input and reagents. MPV was responsible for creating all the figures with input from NS, SWC, and AMT. DH wrote the manuscript with input from MPV. DH conceptualized and supervised the study and obtained funding.

## Funding support

This work is the result of NIH funding, in whole or in part, and is subject to the NIH Public Access Policy. Through acceptance of this federal funding, the NIH has been given a right to make the work publicly available in PubMed Central.

- National Institute of Neurological Disorders and Stroke (NINDS), NIH R01 grants (NS100864 and R21 NS1256000, to DH).
- Ian’s Friends Foundation grant (to DH).
- Icahn School of Medicine at Mount Sinai and Perelman School of Medicine at the University of Pennsylvania seed funding (to DH).
- National Cancer Institute (NCI), NIH K08 Career Development Award (1-K08 CA258796-01, to JCV).
- Robert Wood Johnson Harold Amos Medical Faculty Development Program grant (to JCV).
- Hyundai Hope on Wheels Scholar Hope Grant (to JCV).
- Fund to Retain Clinical Scientists at Yale, sponsored by the Doris Duke Charitable Foundation (award 2015216, to JCV).
- NIH grant R01CA248536 (to EW).
- This work was supported in part by The Tisch Cancer Institute Cancer Center Support Grant (NCI P30 CA196521), which provided shared resources and research infrastructure.

## Supplementary Material

Supplemental data

Unedited blot and gel images

Supporting data values

## Version 1 12/23/2025

In-Press Preview

## Version 2 03/16/2026

Electronic publication
