# Supplementary material for: Mismatch repair deficiency drives malignant progression and alters the tumor immune microenvironment in glioblastoma models
Source: J Clin Invest. 2025 Dec 23;136(6):e195189. doi: 10.1172/JCI195189 (PMC12987617; doi:10.1172/JCI195189)
Supplement: Supplemental data [file jci-136-195189-s087.pdf]

A

Hugo_Symbol	Mis_beningn	Mis_pathogenic	Trunc
EGFR	0.189	0.75305	0.0576
MLH1	0	0.65385	0.3462
MSH2	0	0.58333	0.4167
MSH6	0.5	0.225	0.275
NF1	0	0.12927	0.8707
PMS2	0	0.5	0.5
PTEN	0	0.48061	0.5194
PTPN11	0	0.99405	0.006

B

Gene	HM	No-HM	Ultra-HM	Sum	Freq. No-HM	Freq. HM	Freq. Ultra-HM
MSH2	18	24	0	42	0.571	0.429	0
MSH6	10	20	3	33	0.606	0.303	0.091
MLH1	3	20	4	27	0.741	0.111	0.148
PMS2	1	9	0	10	0.9	0.1	0

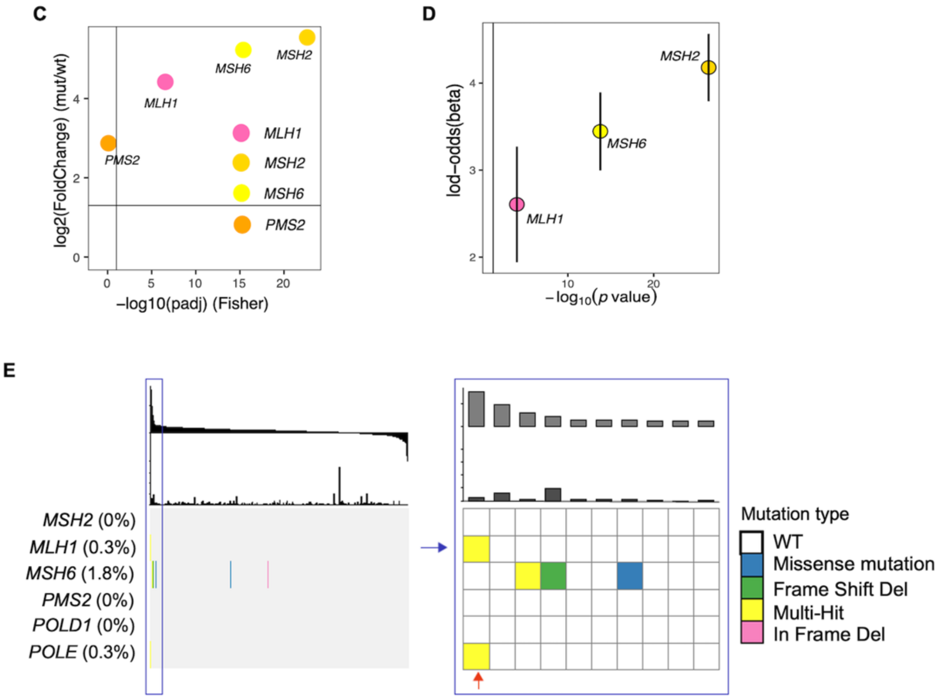

Figure S1. Correlation between TMB and pathogenic mutations in MMR genes. (A) Proportions of mutation types for each gene (for **Figure 1C**). **(B)** Number and frequency of the HM phenotype in GBMs with MMR gene mutations (for **Figure 1D**). **(C)** Correlation between HM/MSI burden and MMR gene mutations (univariate analysis). **(D)** Multivariate analysis assessing the contributions of individual MMR gene mutations to HM phenotype development. **(E)** Co-occurrence of MMR genes with *POLE* mutations creates high TMB and MSI in primary human GBM. Co-occurrence of MMR gene mutations with *POLE* contributes to high TMB in primary human GBM. Oncoprint of nonsynonymous mutations in MMR genes and *POLE/POLD1* in TCGA-GBM patients (n = 284, with 6 altered cases). Red arrows indicate an ultra-hypermutant case showing co-occurrence of *POLE* and *MLH1* mutations. Fisher's exact test for **C**. MSI burden was compared using the Mann–Whitney U test for **D**.

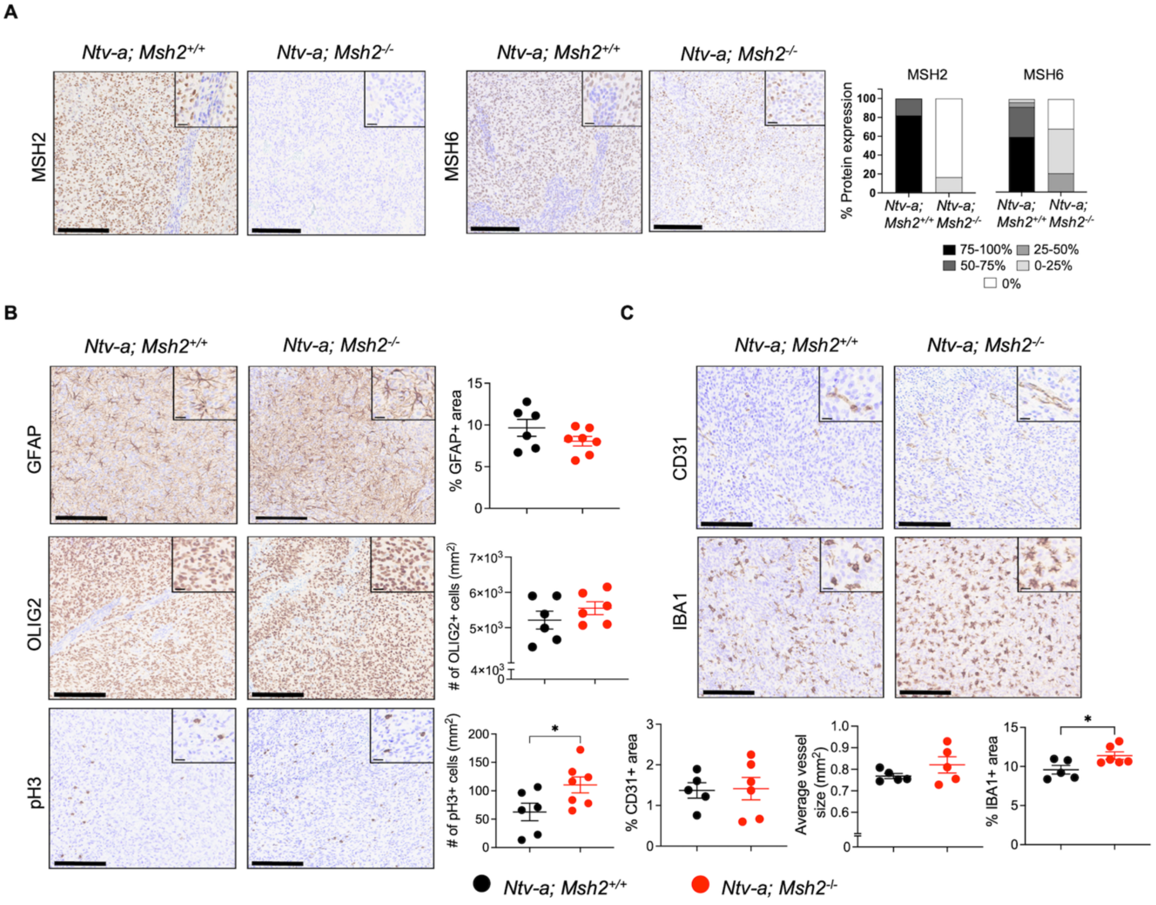

Figure S2. Pediatric MMRd HGG characterized by germline loss of *Msh2* exhibit increased cell proliferation and TAM-positive areas. (**A**) Representative images of MSH2 and MSH6 IHC staining and quantification for tumors in *WT* and *Msh2*-deficient mice. (**B**) Representative images and quantification of IHC for GFAP, OLIG2, and pH3. (**C**) Representative images and quantification of IHC for CD31 (endothelial cells) and IBA1 (TAMs). Student's *t*-test. *$p<0.05$, Scale bar = 100 µm, scale bar in inset = 50 µm for **A**, **B** and **C**.

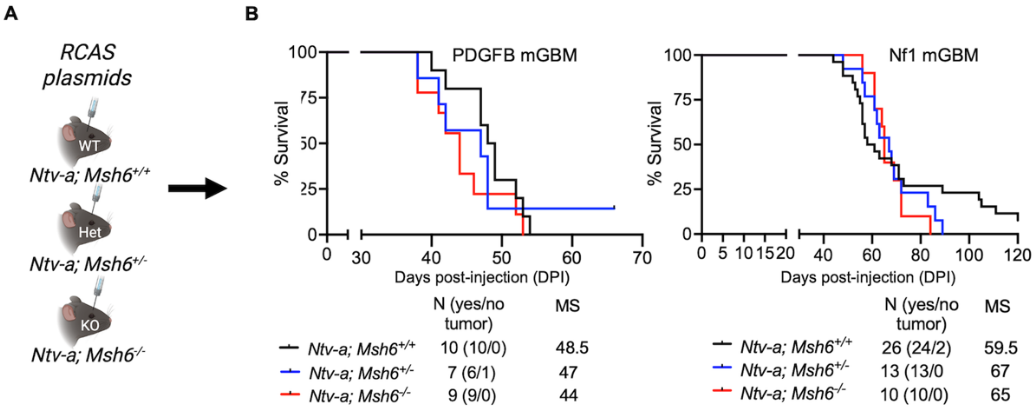

Figure S3: *Msh6*-driven germline MMRd does not confer shorter survival in tumor-bearing mice. (A) Schematic illustration of adult GBM using overexpression of PDGFB and shp53 (PDGFB mGBM) and RCAS-shRNA-Nf1, RCAS-PDGFA, RCAS-shRNA-p53 and RCAS-shRNA-Pten (Nf1 mGBM) in the adult *Ntv-a; Msh62*$^{+/+}$*, Ntv-a; Msh6*$^{+/-}$, and *Ntv-a; Msh6*$^{-/-}$ mice and (**B)** Kaplan-Meier survival curves for various genotypes. MC and GBW test for **B**.

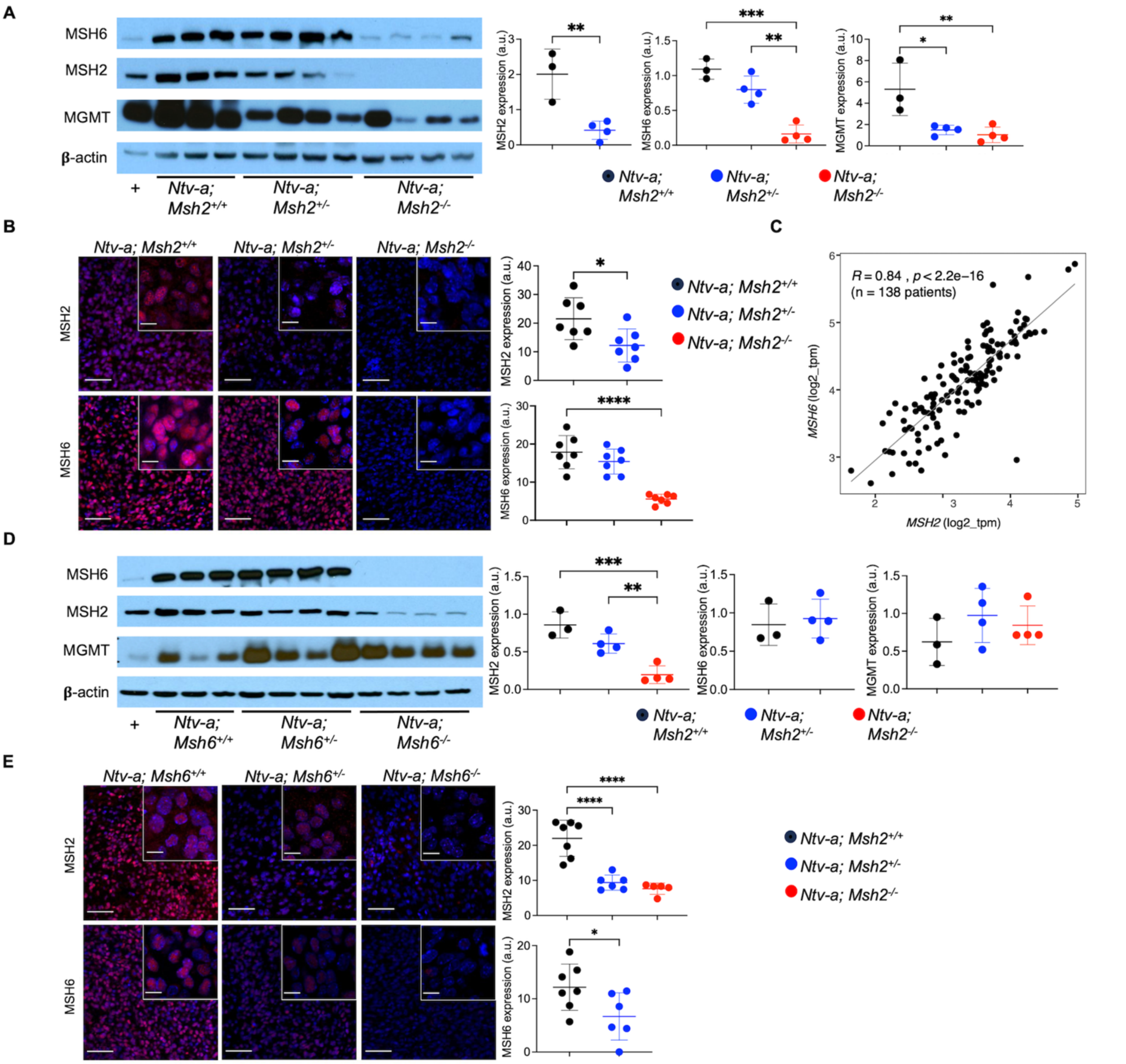

Figure S4. Characterization of adult germline MMRd tumors. (**A**) Western blot of MSH2, MSH6, and MGMT from tumors generated in *Ntv-a; Msh2*$^{+/+}$, *Ntv-a; Msh2*$^{+/-}$, and *Ntv-a; Msh2*$^{-/-}$ mice and corresponding quantifications normalized to b-actin. +: unrelated positive control. (**B**) Representative images and quantification of immunofluorescence for MSH2 and MSH6 in tumors generated in the adult *Ntv-a; Msh2*$^{+/+}$, *Ntv-a; Msh2*$^{+/-}$, and *Ntv-a; Msh2*$^{-/-}$ mice with corresponding quantification graphs. **(C)** Pearson correlation analysis of *MSH2* versus *MSH6* RNA expression in GBM patient samples from TCGA (transcripts per million -TPM). (**D**) Western blot of MSH2, MSH6, and MGMT from tumors generated in *Ntv-a; Msh6*$^{+/+}$, *Ntv-a; Msh6*$^{+/-}$, and *Ntv-a; Msh6*$^{-/-}$ mice and corresponding quantifications normalized to b-actin. +: unrelated positive control. (**E**) Representative images and quantification of immunofluorescence for MSH6 and MSH2 in tumors generated in the adult *Ntv-a; Msh6*$^{+/+}$, *Ntv-a; Msh6*$^{+/-}$, and *Ntv-a; Msh6*$^{-/-}$ mice with corresponding values normalized to b-actin. One-way ANOVA was followed by Tukey's *post hoc* analysis for **A**,

B, **D** and **E**, Student's *t*-test for **A-E**. $^{*}p<0.05$, $^{**}p<0.01$, $^{***}p<0.001$, $^{****}p<0.0001$. Scale bar = 50 μm, scale bar in inset = 20 μm **B** and **E**.

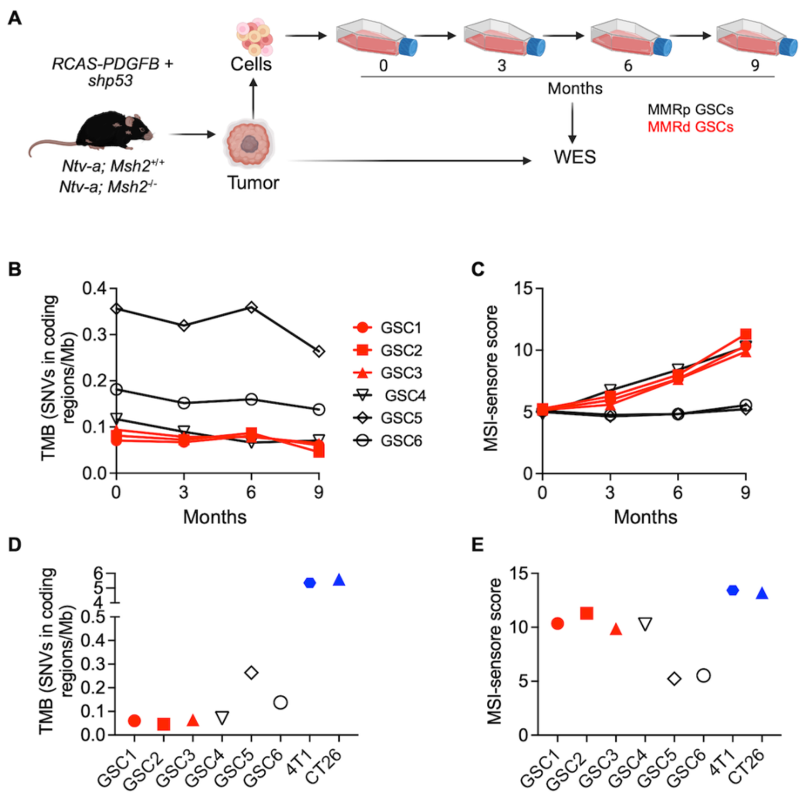

Figure S5. Prolonged culture conditions do not induce increased TMB and MSI in *Msh2*-deficient primary GSC cells. (A) Schematic illustration of WES experiments in cells cultured at different timepoints. **(B)** SNV counts in coding regions per Mb sequenced DNA in *WT* and *Msh2 KO* cell lines cultured for 0, 3, 6 and 9 months. **(C)** MSI analysis in *WT* and *Msh2 KO* cell lines cultured for 0, 6, and 9 months. **(D)** SNV counts in coding regions per Mb sequenced DNA in *WT* and *Msh2 KO* cell lines cultured for 9 months. **(E)** MSI analysis *WT* and *Msh2 KO* cell lines cultured for 9 months. *Msh2* knockdown 4T1 and CT26 cell lines are used as controls, as both show increased TMB and MSI.

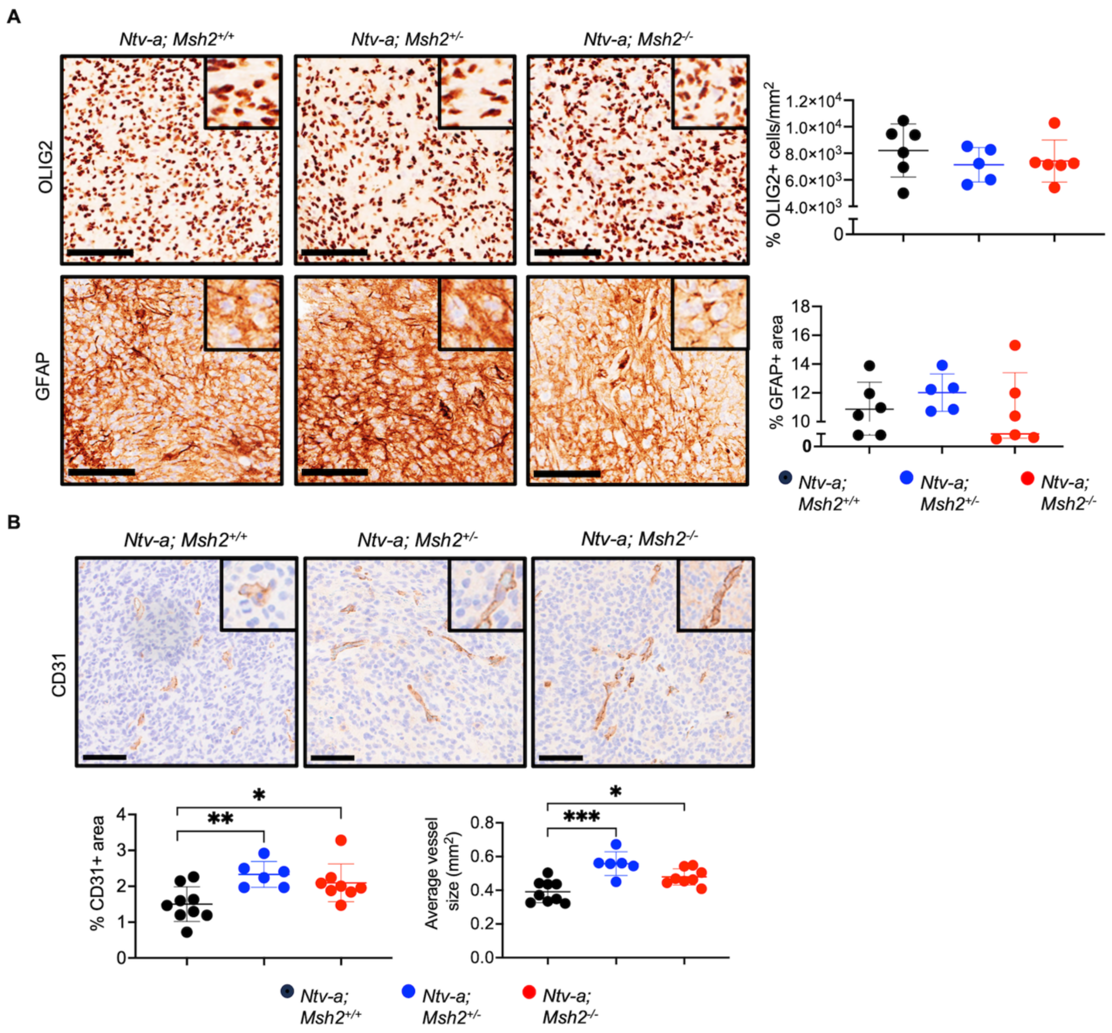

Figure S6. Germline MMRr and MMRd tumors show increased angiogenesis. **(A)** Representative images and quantification of immunohistochemistry for GFAP and OLIG2 in tumors from the adult *Ntv-a; Msh2*$^{+/+}$, *Ntv-a; Msh2*$^{+/-}$, and *Ntv-a; Msh2*$^{-/-}$ mice. **(B)** Representative images and quantification of IHC for CD31 (endothelial cells) in tumors from the adult *Ntv-a; Msh2*$^{+/+}$, *Ntv-a; Msh2*$^{+/-}$, and *Ntv-a; Msh2*$^{-/-}$ mice. One-way ANOVA followed by Tukey's *post hoc* analysis for **A** and **B**. *$p<0.05$, **$p<0.01$. Scale bar = 50 µm, scale bar in inset = 20 µm for **A**; scale bar = 100 µm, scale bar in inset = 50 µm for **B**.

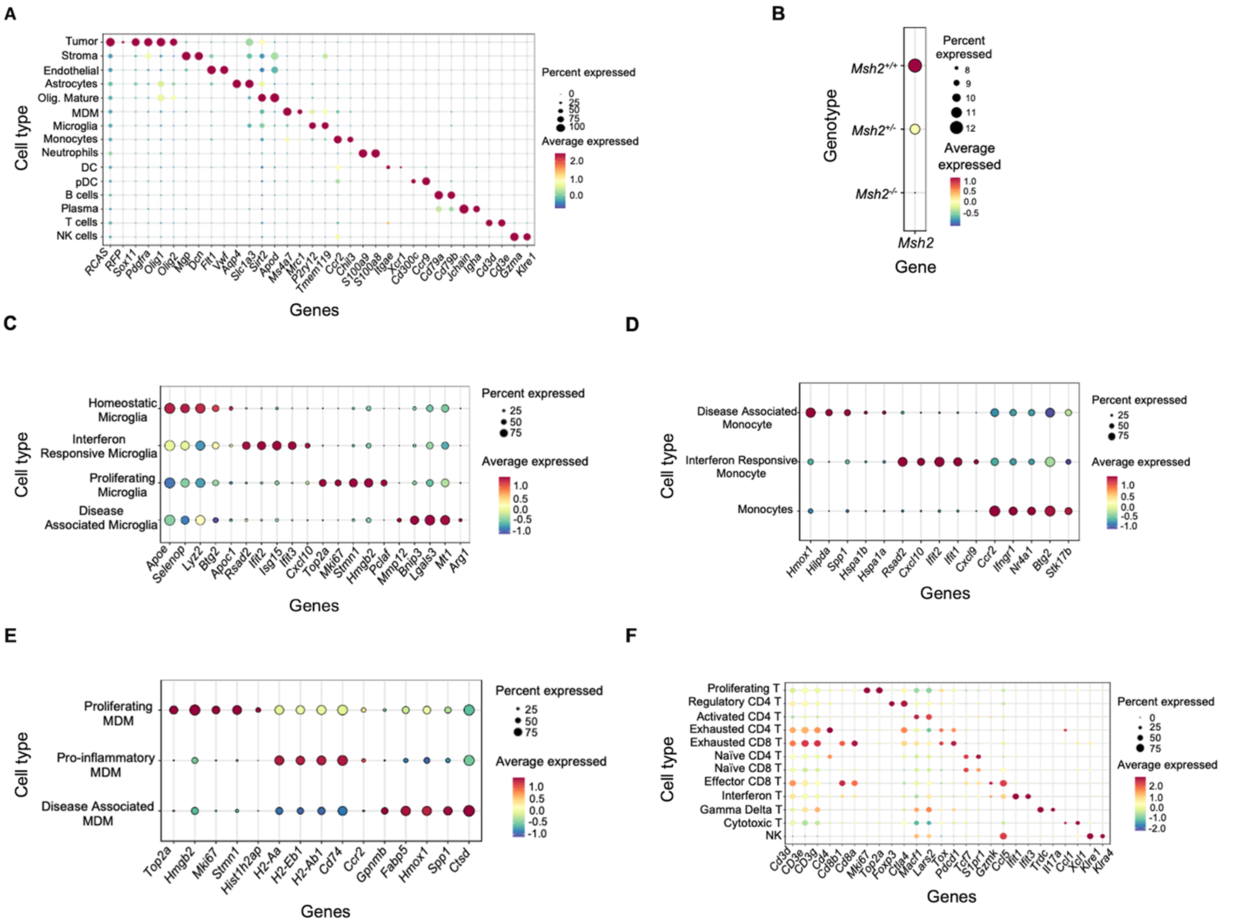

Figure S7. Cell type annotations are based upon expression of selected markers and cataloging myeloid subsets into clusters based upon differentially expressed gene sets. (A) Dot plot showing expression levels and the percentage of cells expressing selected marker genes for each annotated cell type. **(B)** Dot plot showing expression levels and the percentage of neoplastic cells expressing the *Msh2* gene across different *Msh2* genotypes. **(C)** Dot plot showing expression levels and the percentage of cells expressing selected the top 5 marker genes for each annotated microglia, **(D)** monocyte, and **(E)** MDM cell subsets. **(F)** Dot plot showing expression levels and the percentage of cells expressing selected marker genes for each annotated T and NK cell subset.

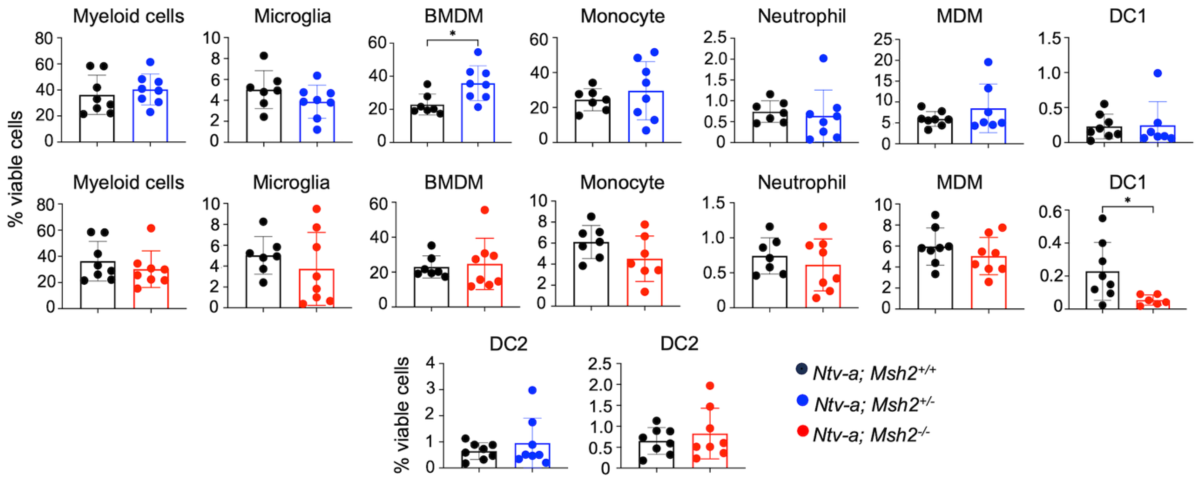

Figure S8. Myeloid profiles of MMRp, MMRr, and MMRd tumors. Dot plots and quantification of myeloid subsets analyzed by spectral flow cytometry (data are included as heatmaps in Figure 4C). Student's *t*-test *p<0.05

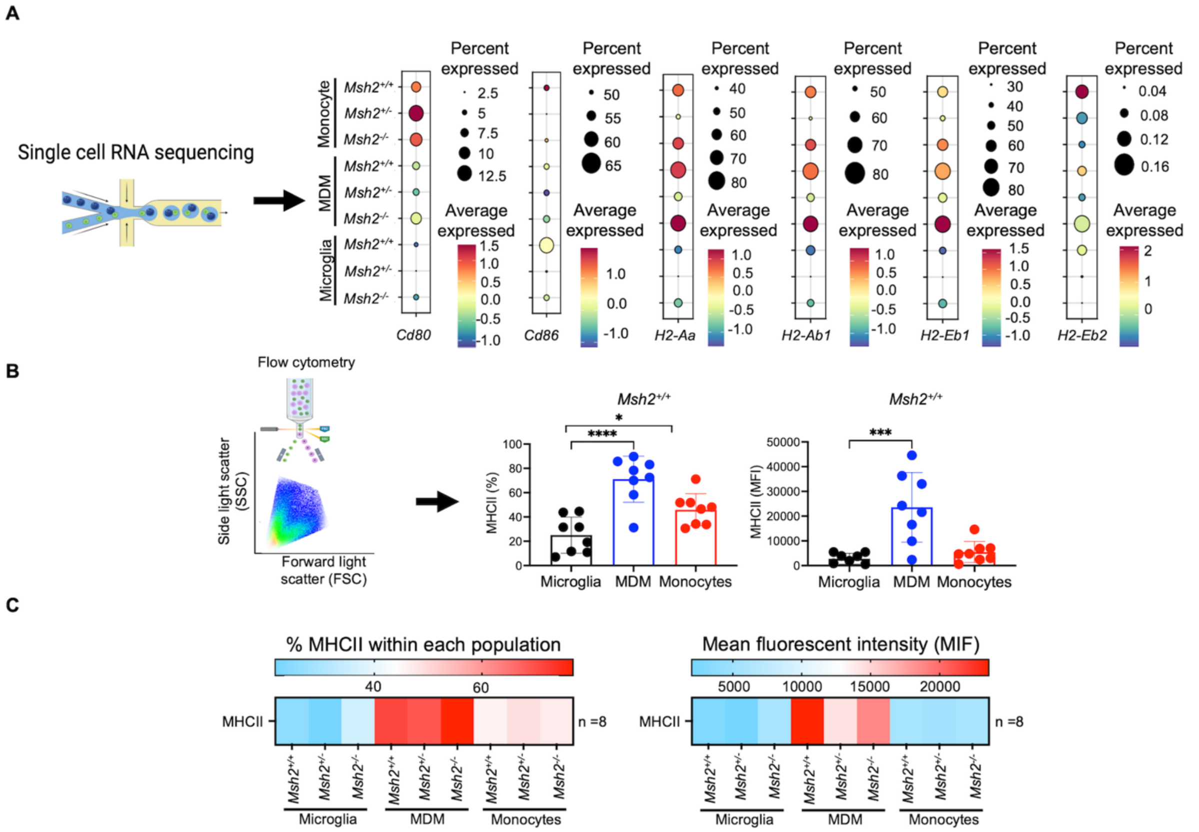

Figure S9. Microglia and macrophages are characterized by low co-stimulatory molecule expression and antigen presentation independent of *Msh2* gene status. (A) Dot plot showing expression levels and the percentage of cells expressing genes encoding co-stimulatory molecules CD80/CD86 and MHCII across microglia, MDM, and monocytes split by *Msh2* genotypes. **(B)** Dot plots and quantification of myeloid subsets analyzed by spectral flow cytometry for the percent expression of MHCII-positive cells and MFI of MHCII in each myeloid subset from WT tumors and **(C)** heatmaps of quantifications split by *Msh2* genotypes. One-way ANOVA followed by Tukey's *post hoc* analysis for **B**. *p<0.05, ***p<0.001, ****p<0.0001.

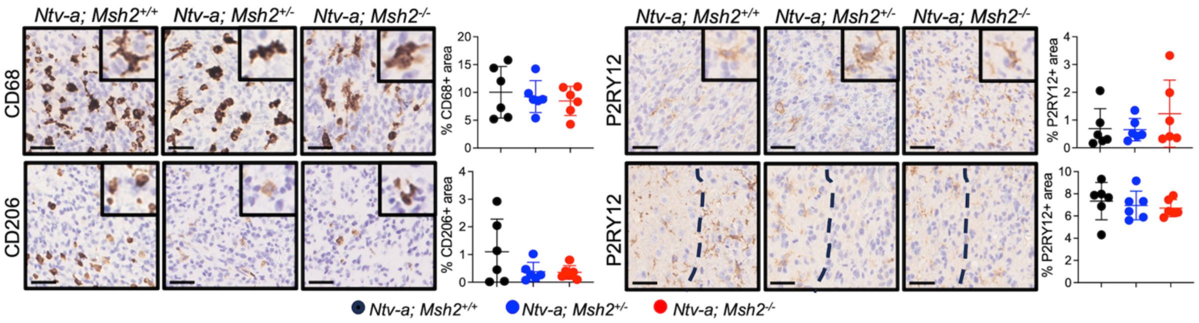

Figure S10. Germline MMR status does not alter macrophage or microglia numbers or CD206 expression. Representative images and quantification of IHC for CD68 (activated macrophages), CD206 (immunosuppressive macrophages) and P2RY12 (microglia) in tumors from the adult *Ntv-a; Msh2*$^{+/+}$*, Ntv-a; Msh2*$^{+/-}$, and *Ntv-a; Msh2*$^{-/-}$ mice. Scale bar = 100 µm, scale bar in inset = 50 µm.

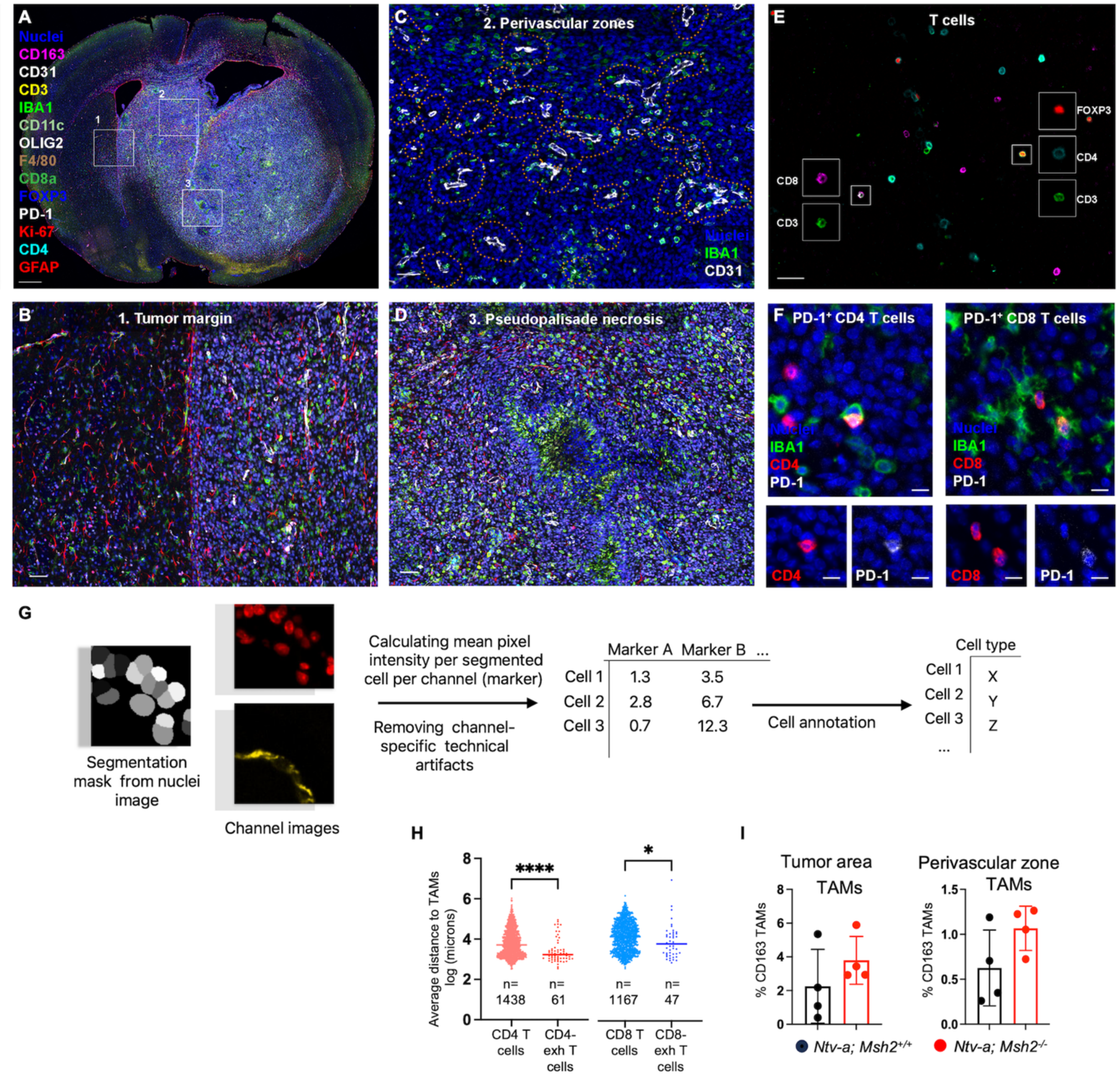

Figure S11. PD-1+ CD8 and CD4 T cells are located observed in close proximity to TMAs. **(A)** Representative images of Orion multiplex IF platform. Overview of a mouse GBM coronal section. Enlarged views showing: **(B)** tumor margin; **(C)** Approximation of 30 µm perivascular extension; and **(D)** Pseudopalisading necrosis. **(E)** T cells are sparsely observed within the tumor bed. **(F)** PD-1^{+} CD4 or CD8 T cells are found in close proximity to IBA1^{+} TAMs. **(G)** Schematic illustration of workflow for image analysis. **(H)** Quantification of average distance of various subsets of T cells to TAMs to T cells. **(I)** Quantification of %CD163+ TAMs in Tumor area and in perivascular zone in MMRp and MMRd tumors. Welch's *t*-test for **H**. Scale bar = 500 µm for **B–D**, 50 µm for **E** and 10 µm for **F**. *p<0.05, ****p<0.0001.

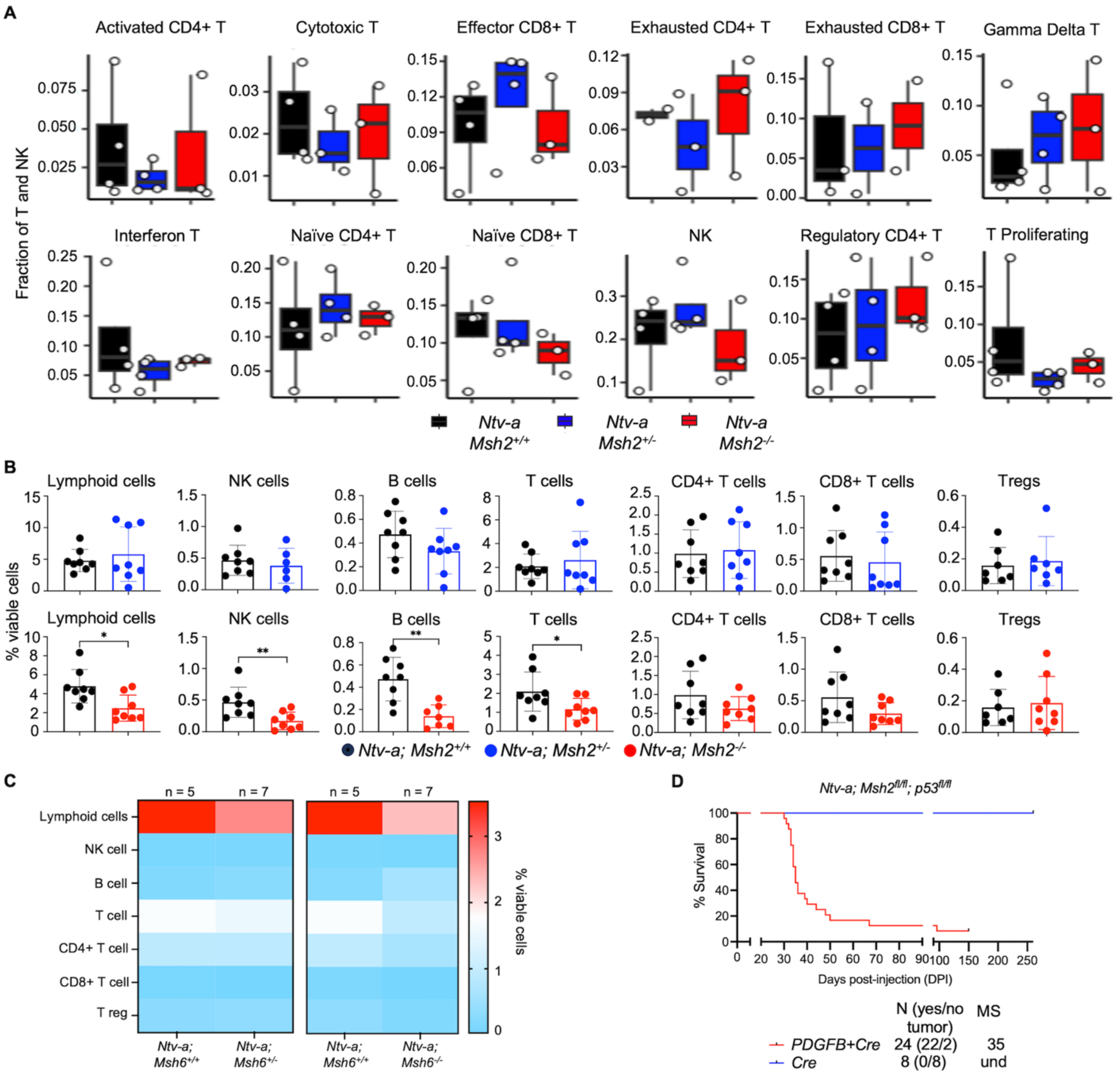

Figure S12. MMRd tumors show lower relative proportions of naïve CD8+ T cells and proliferating T cells. (A) Distribution of proportions of T-cell subsets and NK cells, split by *Msh2* status, black= *WT*, blue = *Msh2 HET* and red = *Msh2 KO* (see also Figure 5B). **(B)** Dot plots and quantification of lymphoid subsets analyzed by spectral flow cytometry (data are included as heatmaps in Figure 5E). **(C)** Heatmap quantification of spectral flow cytometry lymphoid panel in tumors from *Ntv-a; Msh6*$^{+/+}$ (WT), *Ntv-a; Msh6*$^{+/-}$ and *Ntv-a; Msh6*$^{-/-}$ mice showing that MMRr and MMRd tumors driven by germline *Msh6* monoallelic or biallelic loss do not display changes in lymphoid infiltration. **(D)** Kaplan-Meier survival curve and corresponding MS times of *Ntv-a; Msh2*$^{fl/fl}$*; p53*$^{fl/fl}$ mice injected either with RCAS-Cre alone or with the combination of RCAS-PDGFB (curve is also used in Figure 5B) showing that the combined loss of *Msh2* and *p53* in *Nestin*-positive cells in brains of adult mice does not result in tumor formation. Student's *t*-test for **B**, MC and GBW test for **D**. *p<0.05, **p<0.01.

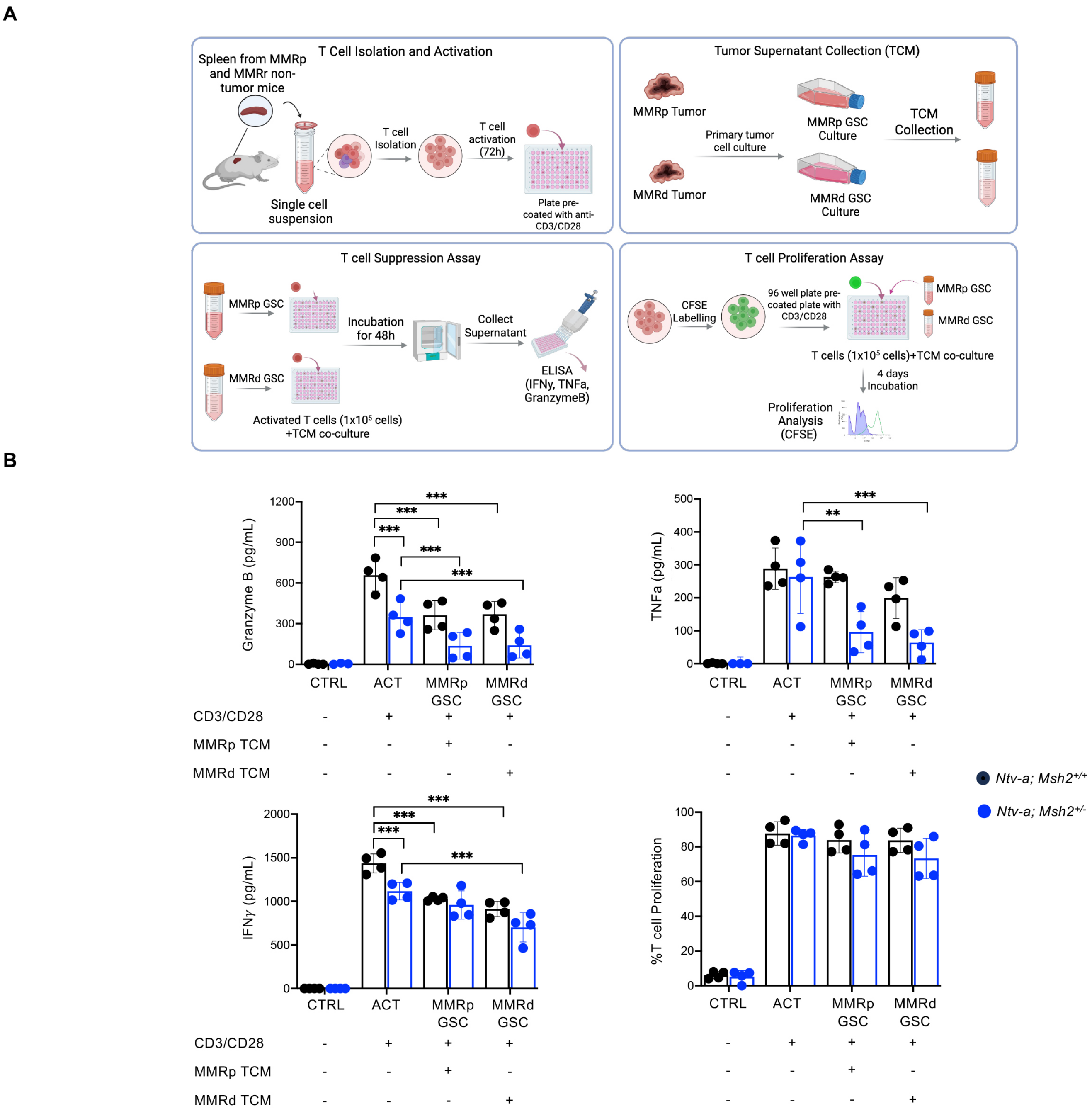

Figure S13. ***Msh2*** **heterozygous loss dampens CD8 T-cell effector function without affecting proliferation and is exacerbated by co-culture with Msh2-deficient GSCs. (A)** Schematic illustration of T cell-tumor cell co-culture experiments. **(B)** Dot plots and quantification of different cytokines release by T cells using ELISA and CFSE dilution by flow cytometry. Two-way ANOVA followed by Šídák's multiple comparison test for **B**. The experiments included three replicates for each genotype, with splenocytes derived from three different mice. **p<0.01, ***p<0.001.

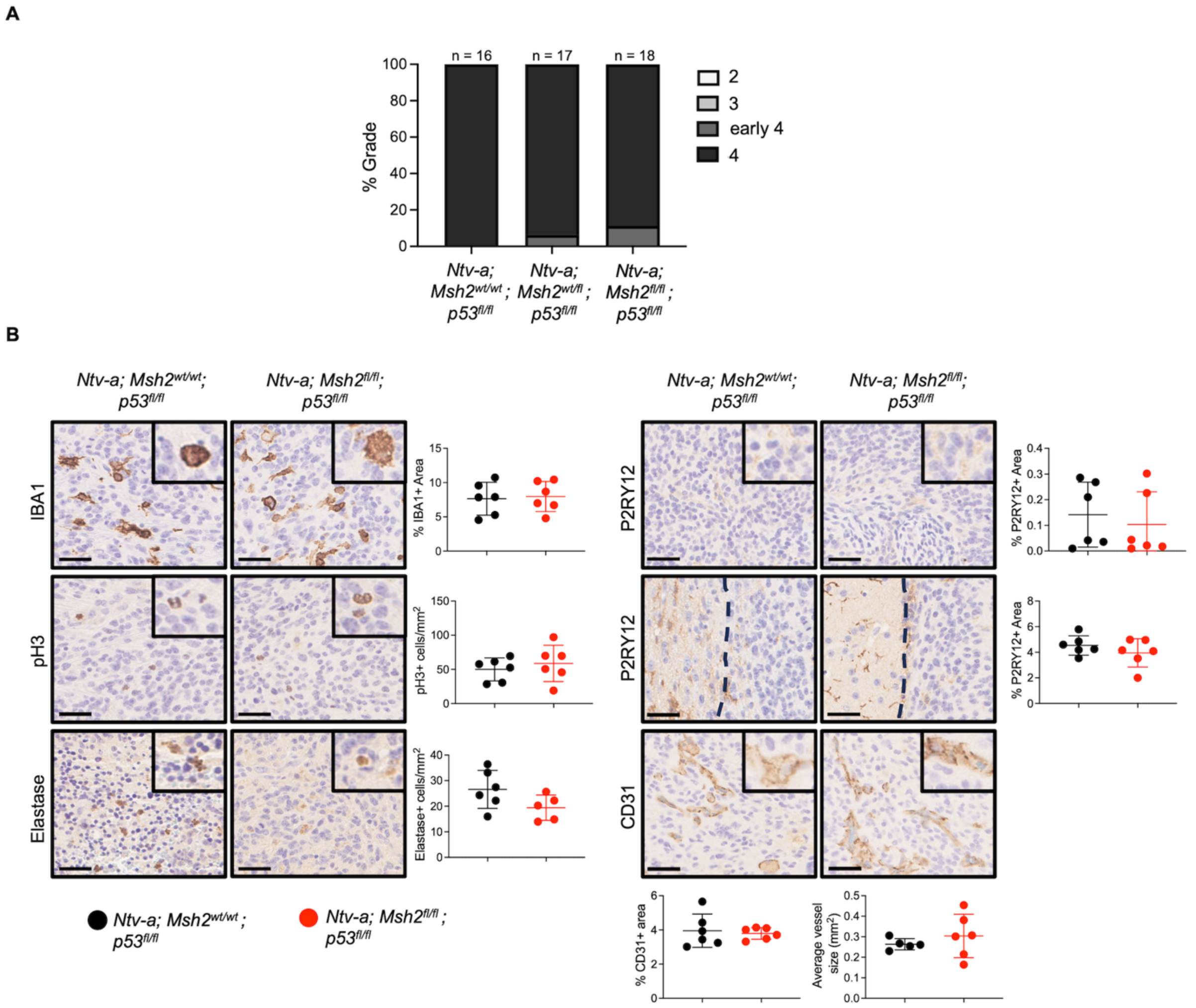

Figure S14. Somatic *Msh2* loss does not promote changes in the tumor microenvironment. (A) Quantification graph of tumor grade for *PDGFB+ Cre* tumors in *Ntv-a; Msh2*$^{wt/wt}$; *p53*$^{fl/fl}$ and *Ntv-a; Msh2*$^{fl/fl}$; *p53*$^{fl/fl}$ genotypes. **(B)** Representative images and quantification of IHC for CD31 (endothelial cells), elastase (neutrophils), IBA1 (TAMs), P2RY12 (microglia) and pH3 (proliferating cells) in PDGFB mGBM from the adult *Ntv-a; Msh2*$^{wt/wt}$; *p53*$^{fl/fl}$ and *Ntv-a; Msh2*$^{fl/fl}$; *p53*$^{fl/fl}$ mce. Scale bar = 100 µm, scale bar in inset = 50 µm for **B**.

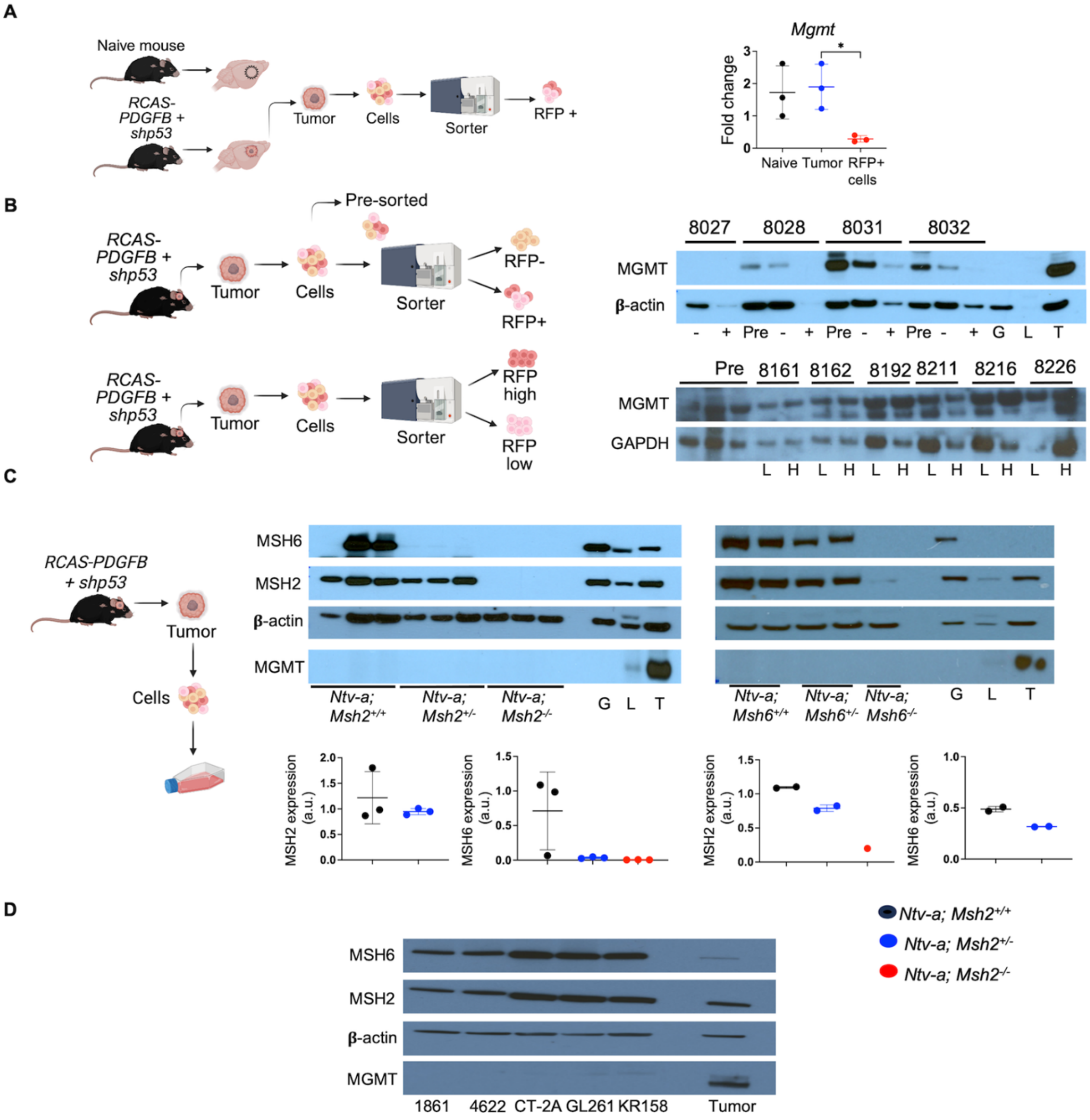

Figure S15: The expression of MGMT differs among tumors and is absent in primary GSCs and established murine GBM cell lines in culture. (A) Diagram showing the isolation of RFP-positive tumor cells and quantitative PCR results for *Mgmt* expression in normal brain tissue, tumors, and sorted RFP+ tumor cells. **(B)** Diagram illustrating the sorting of tumor and non-tumor cells, as well as RFP-high and RFP-low tumor cells, along with the corresponding western blots for MGMT. (**C**) Schematic demonstrating the isolation and culturing of freshly-dissociated tumor cells from different genotypes and the western blot results for MSH2, MSH6, and MGMT expression, along with corresponding quantification. **(D)** Western blot results for MSH2, MSH6, and MGMT expression in established murine cell lines. G = GL261, L = liver, T = tumor. One-way ANOVA followed by Tukey's *post hoc* analysis for **A** and **C**, Student's t-test for **C**. *$p<0.05$.

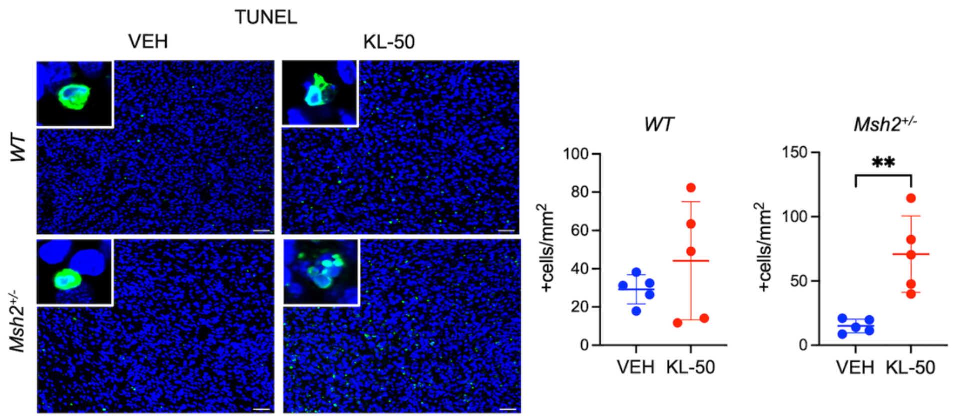

Figure S16. KL-50 induces apoptosis in treated MMRr tumors. Representative images of TUNEL staining and quantification of TUNEL-positive cells in MMRp and MMRr/MMRd tumors treated with VEH or KL-50 for 5 days and sacrificed 24 hours after the last dose. Student's *t*-test for *p<0.05. Scale bar = 50 µm, scale bar in inset = 20 µm.

Supplemental Methods

Mice

Msh2$^{-/-}$ mice were purchased from the Jackson Laboratory (#042057) (1) and *Msh6*$^{-/-}$ mice were provided by co-author Dr. Winfried Edelmann (2). Afterward, they were crossed with *Ntv-a* transgenic mice to generate: *Ntv-a; Msh2*$^{+/-}$*, Ntv-a; Msh2*$^{-/-}$, *Ntv-a;Msh6*$^{+/-}$*,* and *Ntv-a;Msh6*$^{-/-}$ crosses. *Tp53*$^{fl/fl}$ (#008462) (3), *Msh2*$^{fl/fl}$ (#016231) (4)*, CD4-Cre* (#017336) (5, 6)*, CD8a-Cre* (#008766) (7)*, Cx3cr1-Cre* (#025524) (8) mice were obtained from the Jackson Laboratory, and crossed to *Ntv-a* transgenic mice to generate the following crosses: *Ntv-a;Msh2*$^{fl/fl}$*;p53*$^{fl/fl}$*, Ntv-a;Msh2*$^{fl/fl}$*;Pten*$^{fl/fl}$*, Ntv-a;Pten*$^{fl/fl}$*, Ntv-a; p53*$^{fl/fl}$ *Cx3cr1-Cre;Ntv-a;Msh2*$^{fl/fl}$*, CD8a-Cre; ;Ntv-a;Msh2*$^{fl/fl}$ *and CD4-Cre Ntv-a;Msh2*$^{fl/fl}$ mice. All control mice for each cell type–specific Cre line were selected based upon Cre negativity from the same breeding cage. All animals were housed in a climate-controlled (18°C–23°C and 40%–60% humidity), pathogen-free facility, with access to food and water *ad libitum*, under a 12 h–12 h light–dark cycle.

Virus generation and tumor induction

DF-1 cells (ATCC, CRL-12203) were purchased and grown at 39°C according to the manufacturer's instructions. Cells were transfected with RCAS-*hPDGFB-HA*, RCAS-*hPDGFA*-myc, RCAS-shRNA-*Nf1*, RCAS-shRNA-*Pten*-Rfp, RCAS-shRNA-*p53*-Rfp, and RCAS-*Cre* using a Fugene 6 transfection kit (Roche, 11814443001) according to the supplier's instructions. DF-1 cells ($4x10^4$) in 1 µl neurobasal medium were stereotactically delivered with a Hamilton syringe equipped with a 30-gauge needle for tumor generation (9). For adult injections, the specific coordinates for PDGFB-overexpressing tumors were in the right-frontal striatum at AP-1.7mm and right -0.5mm from bregma; depth -1.5 mm from the dural surface. Nf1-silenced tumors were generated via injection into the subventricular zone at coordinates AP -0.0 mm and right -0.5 mm from bregma; depth -1.5 mm from the dural surface (10, 11). For pediatric injections, pups at age P0-P3 were injected based upon our published protocols (12, 13). Mice were continually monitored for signs of tumor burden and were sacrificed upon observation of endpoint signs, including head tilt, lethargy, seizures, and >15% weight loss.

GBM cell lines

The murine MES glioma cell lines 1861 and 4622 (14, 15) and the murine glioma cell lines GL261, CT-2A, and KR158 (14) were provided by Dr. Roland Friedel (Icahn School of Medicine, New York) and were grown in DMEM (Gibco, 11995065) supplemented with 10% Fetal Bovine Serum (FBS; ATCC, 30-2020), 1% Penicillin-streptomycin (Fisher, 15140122), and 1% Glutamax (Gibco, 35-050-061). The hypermutant murine cell lines CT26 *Msh2*$^{-/-}$ (colon carcinoma cell) and 4T1 *Msh2*$^{-/-}$ (breast cancer cell) were provided by co-author Dr. Robert Samstein (Icahn School of Medicine, New York) (16) and were grown in RPMI-1640 (Corning, 22400089) with 10% FBS (ATCC, 30-2020) and 1% Penicillin-streptomycin (Fisher, 15140122). All of the above-mentioned cell lines were grown at 37°C in a humidified atmosphere containing 5% CO_2.

Tumor dissociation, cell sorting, and primary tumor cell cultures

Briefly, tumors were dissected from the brain, minced into pieces < 1 mm^3, and digested with an enzymatic mixture that includes papain (0.94 mg/ml; Worthington, LS003120), EDTA (0.18 mg/ml; Sigma, E6758), cystine (0.18 mg/ml; Sigma, A8199), and DNase (60 µg/ml; Roche, 11284932001) in 5 ml EBSS (Gibco, 14155063). Tumor tissues were kept at 37°C for 15 minutes

with occasional agitation. The digestion was terminated with the addition of 2 ml Ovomucoid (0.7 mg/ml; Worthington, LS003086). Following digestion, single cells were pelleted, resuspended in HBSS, passed through a 70 µm cell strainer, and centrifuged at low speed (400 RCF) for 5 min.
Cell sorting- Cells were blocked with 100 µl of blocking solution (2% FBS, 5% normal rat serum, 5% normal mouse serum, 5% normal rabbit serum, 10 µg/ml anti-FcR (BioLegend, 101319) and 0.2% NaN_3 in DPBS) on ice for 15 minutes. Cells were then stained with CD45-FITC and CD11b-PerCP-Cy5.5 (Supplemental Table 1) on ice for 30 minutes and washed with PBS. The cells were subsequently gated based upon CD45 and CD11b expression and sorted based upon RFP signal using the AuroraCS. Afterwards, protein was extracted from pellets of RFP-positive and RFP-negative (CD45+/CD11b+) cells to perform immunoblot analysis.
Cell culture- Cells were seeded at 5 x 10^5 cells/ml in flasks previously coated with Geltrex LDEV (ThermoFisher, A1413201) for 2h and grown as neurostem cells (NCS) using Neurocult mouse neurobasal medium (Stem Cell Technologies, 5700) supplemented with 10 ng/ml hEGF (Lonza, cc-4017FF), 20 ng/ml basic-hFGF (ThermoFisher, PHG0261), 1 mg/ml Heparin (Stem Cell Technologies, 7980), and NSC Proliferation Supplements (Stem Cell Technologies, 5701). Cell lines were grown at 37°C in a humidified atmosphere containing 5% CO_2. Fresh medium was added to the cultures every 48 hours.

***In vivo* αPD-1, temozolomide, and KL-50 treatments**
Survival experiments- αPD-1 (10mg/kg mouse weight; BioXcell, BE0146) or αIgG2a (10 mg/kg mouse weight; BioXcell, BE0089) were intraperitoneally administered to mice every 3 days until humane endpoint. Treatment was initiated 14 days post-RCAS injections.
Survival experiments- Temozolomide (25 mg/kg mouse weight; Sigma, T2577) or a corresponding volume (10 ul/g mouse weight) of 0.9%; 10% DMSO solution (McKesson, 2718344) was orally administered to mice for a schedule of five days on, two days off, five days on (total =10 doses). Treatment was initiated 25 and 30 days post-RCAS injection for the germline model and 19 days post-RCAS injection for the somatic MMR model.
Assessing immediate effects of TMZ- Temozolomide (25 mg/kg mouse weight; Sigma, T2577) or a corresponding volume (10 ul/g mouse weight) of 0.9%; 10% DMSO solution (McKesson, 2718344) was orally administered to mice for a schedule of five days on, two days off, five days on (total = 10 doses). Treatment was initiated 35 days post-RCAS injection in *Ntv-a; Msh6*$^{+/+}$ mice and 32 days post-RCAS injection in *Ntv-a; Msh6*$^{-/-}$ mice. Blood was collected one day before starting treatment and on treatment day 7. Immune populations within tumors were assessed at the experimental endpoint.
Survival experiments– KL-50 (25 mg/kg mouse weight) and vehicle (10% (2-Hydroxypropyl)-beta-cyclo-dextrin (Sigma, 332607)) were administrated for five consecutive days starting from 25 days post-RCAS injections in *Ntv-a; Msh2*$^{+/+}$ and *Ntv-a; Msh2*$^{+/-}$ mice and 19 days post-injection for *Ntv-a;Msh2*$^{fl/fl}$*,p53*$^{fl/fl}$ and *Ntv-a;Msh2*$^{wt/wt}$*,p53*$^{fl/fl}$ mice. Mice were kept for survival and sacrificed at the humane experimental endpoint when they displayed neurological signs of tumor burden, including head tilt, lethargy, seizures, and >15% weight loss.
Assessing immediate effects of KL-50– KL-50 (25 mg/kg mouse weight) and vehicle (10% (2-Hydroxypropyl)-beta-cyclo-dextrin (Sigma, 332607)) were administrated for five consecutive days, two days off, three days on starting from 30 days post-RCAS injections in *Ntv-a; Msh2*$^{+/-}$ mice and 35 days post-injection for *Ntv-a; Msh2*$^{+/+}$ mice. Blood was collected one day before starting treatment and on treatment day 12. Mice were sacrificed 24 hours after the last dose. To reinforce the results obtained, mice were scanned via MRI when they showed early neurological

symptoms. When tumors reached >5 mm^3, KL-50 or vehicle was administered orally for 5 days. Mice were scanned one day later and sacrificed to perform experiments.

MRI image acquisition

We used a 9.4 T, 20 cm horizontal bore Bruker magnet connected to an Avance console (Bruker) and equipped with an actively shielded gradient set. This setup has an inner diameter of 11 cm, a maximum gradient strength of 100 mT/m, and a rise time of 110 ms. We employed a two-coil actively decoupled imaging setup with a 3 cm surface coil receiving the signal generated by a 7.2 cm diameter volume transmission coil. The receiving coil was positioned over the cortical and subcortical areas of interest to maximize the signal-to-noise ratio. Mice were imaged while anesthetized with 1.5-2% isoflurane (Baxter) and held in a custom-made cradle. Body temperature and respiration rate were continually monitored throughout the experiment with an animal physiological monitoring system (SA Instruments). The mouse head was imaged in the axial orientation using a T2-weighted fast spin-echo RARE sequence with a TR of 3.5 seconds, a TE of 50 ms, a RARE factor of 8, a NEX value of 24, a field of view of 3 x 2 cm, and a slice thickness of 0.7 mm generating an in-plane resolution of 117x156 mm. A total of 21 slices were obtained during the procedure.

Tumor grading

At the experimental endpoint, mice were sacrificed with an overdose of ketamine (300 mg/kg) and xylazine (30 mg/kg) and perfused with Ringer's solution. The brain was carefully extracted and fixed in formalin for 72h. Afterwards, brains were embedded in paraffin and 5 µm FFPE sections were stained for automated hematoxylin and eosin (H&E) staining. H&E staining was assessed under a light microscope with high resolution optics (Nikon) and graded by a neuropathologist in four categories based on the latest clinical criteria used for grading diffuse glioma in patients (17). *Grade 2:* lack of brisk mitotic activity (< 3 mitotic figures in the entire section examined) and nuclear atypia, such as pleomorphism, nuclear hyperchromasia, and prominent nucleoli. *Grade 3*: nuclear atypia and increased mitotic activity (observation of 3 or more mitotic figures within the examined tumor section, lack of evidence of microvascular (endothelial) proliferation or pseudopalisading necrosis). *Early 4*: notably, some tumors were noted in a transition state from grade 3 to *bona fide* grade 4, with a presence of early microvascular proliferation/endothelial hyperplasia but lack of pseudopalisading necrosis. These tumors were designated as "early grade 4" to capture the subtle dynamics of HGG transformation. *Grade 4*: microvascular proliferation in combination with pseudopalisading necrosis (17). All tumors were analyzed in a blinded fashion.

Immunoblot analysis

Mouse primary GBM primary cultures from various genotypes were washed 2x with ice-cold PBS and scraped into RIPA buffer (50 mM Tris, pH 8.0, 150 mM NaCl, 5 mM EDTA, 1% NP40 and 0.5% deoxycholate, 10 mM NaF, 1 mM sodium orthovanadate with complete Halt™ protease and phosphatase inhibitor cocktail) (ThermoFisher, 78443), incubated on ice for 30 min, centrifuged, and supernatants were collected. Tumor samples were lysed into RIPA buffer (50 mM Tris, pH 8.0, 150 mM NaCl, 5 mM EDTA, 1% NP40 and 0.5% deoxycholate, 10 mM NaF, 1 mM sodium orthovanadate with complete Halt™ protease and phosphatase inhibitor cocktail) (ThermoFisher, 78443) with a sonicator, centrifuged, and supernatants were collected. Protein concentrations from cells and tumor tissue were determined using a Bradford protein assay (BioRad, 5000001). Cell lysates (20-30 µg) were subjected to SDS-polyacrylamide gel electrophoresis on 4-12% precast

gradient gels (BioRad, 5678094) and proteins were transferred to nitrocellulose membranes (Bio-Rad, 162-0112). After transfer, membranes were incubated in 5% Nonfat Dry Milk (Bio-Rad, 9999) in 1x TBS-T (Cell Signaling 9997) for 1 hour. Membranes were incubated overnight at 4°C with primary antibodies: MGMT (E-1) (1:4000; Santa Cruz Biotechnology, sc-166528), MSH2 (1:1000; Cell Signaling, 2017S), MSH6 (1:5000; Abcam, ab92471), and β-actin was used as a loading control (1:50000; Abgent, ABIN1842939) in TBS-T (Cell Signaling, 9997). Immunodetection was performed with anti-rabbit horseradish-peroxidase-conjugated secondary antibody (1:15000; Cell Signaling, 7074P2) and anti-mouse horseradish–conjugated secondary antibody (1:20000; Cell Signaling, 7076P2). Immunodetection was performed with Chemiluminescent HRP Antibody Detection Reagent (Denville Scientific, INC E2400). Detailed information for the antibodies used is provided in Supplemental Table 1.

Tissue processing and immunohistochemistry

Tumor-bearing mice were anesthetized with an overdose of ketamine/xylazine (300/30 mg/kg) mix and transcardially perfused with ice-cold Ringer's solution (TermoFisher, BR0052G). Brains were removed and processed according to the different applications. For H&E staining to evaluate tumor presence and grade, and for immunohistochemistry staining, brains were fixed in 10% neutral buffered formalin for 72 hours at room temperature (RT), processed in a tissue processor (Leica TP1050), embedded in paraffin, sectioned (5 µm), and slide-mounted.

All immunohistochemistry staining was performed on a Bond Rx (Leica Biosystems). Primary antibodies used in this study include anti-CD31 (1:250; Dianova, DIA-310), anti-CD68 (1:1000; Abcam, AB303565), anti-CD206 (1:200; Cell Signaling, 24595), anti-elastase (1:400; Bioss, bs-6982R), anti-IBA-1 (1:500; Wako, 019-19741), anti-OLIG2 (1:500; Millipore, AB9610), anti-phosphorylated Histone 3 (1:400; Millipore, 06-570), anti-P2RY12 (1:500; AnaSec, AS35043A), anti-GFAP (1:10000; Cell Signaling, 3670), anti-MSH2 (1:1000; Cell signaling, 2017S) and anti-MSH6 (1:600; Abcam, ab92471). Digital images of the slides were acquired by using a Nanozoomer 2.0HT whole-slide scanner (Hamamatsu Photonic K.K) and observed offline with NDP view2 software (Hamamatsu). Image analysis was performed using ImageJ Fiji. For each staining and sample, multiple representative images were acquired. For IBA1, OLIG2, GFAP, CD31, CD68, CD206 and P2RY12 the percentage of positively-stained tumor area was calculated using the threshold function in ImageJ Fiji. For MSH2 and MSH6, two independent reviewers (MP and AB) assigned a relative score of 0 (0-25%) to 4 (75-100%) to each image based upon staining intensity and the average scores were calculated. For phospho-histone3 and Elastase, the number of positively-stained nuclei per square millimeter was quantified. Detailed information for the antibodies used is provided in Supplemental Table 1.

Immunofluorescence

5 µm murine tumor FFPE sections were stained with anti-MSH2 (1:250; Cell signaling, 2017S) and anti-MSH6 (1:250; Abcam, ab92471). Secondary antibodies conjugated to Alexa-Fluor dyes wavelengths of 594 nm (Jackson ImmuneResearch, 711-587-003) and 555 nm (Life Technologies, A31572) at a dilution of 1:500 in PBS/2% BSA were applied. DAPI was used for nuclear counterstaining (Sigma, D9542). Fluorescence images were taken on a Leica SP8 Inverted confocal microscope and analyzed using FIJI software. For each staining and sample, multiple representative images were acquired. MSH2 and MSH6 were quantified based on positive nuclear staining intensity using the threshold function in ImageJ Fiji. Detailed information for the antibodies used is provided in Supplemental Table 1.

Multiplex Immunofluorescence Imaging by Orion

Formalin-fixed, paraffin-embedded (FFPE) tissue sections were deparaffinized, rehydrated, and subjected to antigen retrieval by submersion in retrieval buffer (BioGenex, HK547-XAK) followed by microwaving (BioGenex, EZ-RT-MW016-IR) for 10 minutes. Non-specific protein binding was blocked using a mixture of normal sera derived from mouse (Millipore Sigma, M5905), rabbit (Millipore Sigma, R4505), and goat (Thermo Fisher Scientific, 16210064). A cocktail containing 13 fluorophore-conjugated antibodies (Supplemental Table 2) was then applied to the slides and incubated for 2 hours at room temperature. Unbound antibodies were removed by washing. Hoechst 33342 (Thermo Fisher Scientific, H3570) was added to the sections at 1:500 dilution for 30 min at RT. Slides were coverslipped with ArgoFluor™ Mounting Medium (RareCyte, 42-1214-000) and loaded onto the Orion (RareCyte) platform for whole-slide image acquisition. The Orion system (18) is equipped with seven high-power excitation lasers and sensitive CMOS detectors, coupled with optical filters providing 90–95% transmission efficiency. These filters enabled the collection of 10–15 nm bandpass channels with 1-nm center wavelength (CWL) tuning selectivity. Following image acquisition, tiles were stitched, distortion-corrected, and processed using a pre-established spectral unmixing matrix. Matrix coefficients were derived by scanning single-stained spectral controls to capture both target fluorophore emission and off-target fluorescence across all Orion channels. Using RareCyte's Artemis software, proprietary algorithms calculated correction factors for spectral crosstalk between donor and recipient fluorophores, as well as for tissue and background autofluorescence. The finalized spectral unmixing matrix was then applied to multiplexed image data to retain true fluorophore-specific signal intensity while removing non-specific crosstalk and autofluorescence. Image files were exported as pyramidal, multi-layered OME-TIFF files.

Orion Data Analysis

Fluorescence image segmentation was performed using the StarDist Python API (v0.9.1) (19); in Python v3.10.18, applied to the Hoechst nuclear channel. Segmented nuclei were subsequently expanded by up to 5 μm from the nuclear boundary to approximate cytoplasmic regions, resulting in whole-cell masks. Following segmentation, cell masks were overlaid with raw fluorescence images, and the mean pixel intensity per cell was computed for each channel. Morphometric features -- including nuclear area, eccentricity, solidity, and centroid coordinates (X, Y) -- were extracted from the nuclei channel using the scikit-image Python package (v0.25.2) (20); Channel-specific technical artifacts were visually inspected and removed using napari (21). Segmented cell masks were overlaid with log-normalized fluorescence signals to determine channel-specific thresholds distinguishing positive and negative cells. These thresholds were subsequently used to rescale fluorescence intensities to a 0-1 range using the scimap Python package (v2.3.5) (22); For the Ki67 channel, cells with scaled channel values ≥ 0.5 were classified as positive for that marker. All samples were also annotated using Boolean logic operations implemented in Python package scimap (v2.3.5) (22). A predefined reference table specifying marker "positivity" and "negativity" for each cell type was used to assign identities. For example, CD163+ TAMs were defined as negative for OLIG2, GFAP, Foxp3, CD8a, CD31, and positive for F4/80, CD163, IBA1. The 30-μm endothelial zone was defined as the set of cells whose nuclear centroids were located within ≤30 μm of any endothelial cell nucleus. Cells outside this distance were categorized as "Other". Distance computations were performed using the scipy package (v1.12.0) (23). Spatial distances between CD4/CD8 exhausted T cells (defined as CD4/PD-1+ T and CD8/PD-1+ T cells),

CD4/CD8 non-exhausted T cells, and TAM population were calculated using the distance function implemented in scimap (v2.3.5) (22). Briefly, the function computes the average Euclidean distance from the nuclear centroid of each query cell to all nuclear centroids belonging to the target cell population.

Cell viability assay

MMRp (GSC4, GSC5 and GSC6) and MMRd (*Msh2 KO)* cell lines (GSC1, GSC2 and GSC3) (5000 cells/well) were seeded in a 96-well plate. Cell viability was assessed 24, 48, and 72h post-seeding using the Cell-Titer 96 One Solution Aqueous Proliferation Kit (Promega, G3582). The absorbance was measured using a Biotek Cytation 5 Imaging reader (Agilent, DE73671223).

T cell suppression assay

To obtain T cells, fresh spleens were harvested from *Ntv-a; Msh2*$^{+/+}$ and *Ntv-a; Msh2*$^{+/-}$ mice, under sterile conditions, and single-cell suspensions were prepared by passing through a 70 µm nylon cell strainer in cold PBS. T cells were isolated using a EasySep mouse T cell isolation kit (STEMCELL Technologies, 19851) according to the manufacturer's instructions. Purified T cells were resuspended in RPMI-1640 medium supplemented with 10% fetal bovine serum (FBS), 2 mM L-glutamine, 1% penicillin-streptomycin, 1X MEM Non-Essential Amino Acids Solution, and 50 µM β-mercaptoethanol. Ninety-six-well round-bottom plates were precoated with anti-CD3 (1 µg/mL; BioLegend, 100201) and anti-CD28 (5 µg/mL; BioLegend, 102101) antibodies overnight at 4°C. Before seeding cells, 96-well plates were washed twice with PBS to remove unbound antibodies and T cells were plated at a density of 1×10^5 cells/well and incubated for 72 hours at 37°C in a humidified 5% CO_2 incubator. Tumor supernatant was collected from cultured tumor cells, centrifuged to remove debris, and filtered through a 0.22 µm filter. Activated T cells were treated with tumor supernatant at a final concentration of 50% v/v for 48 hours. After 48 hours of treatment, the supernatant was collected and centrifuged to remove any cells or debris. The levels of cytokines IFN-γ (Invitrogen, 501125445), TNFa (Invitrogen, 51125453), and Granzyme B (Invitrogen, BMS6062) were measured by ELISA as per the manufacturer's protocol. For proliferation analysis, T cells were labelled with CellTrace™ CFSE (Invitrogen, C34554) as per manufacturer's instructions before they were plated at 1×10^5 cells per well in a round-bottom 96-well tissue culture plate precoated with CD3 and CD28 antibodies. Tumor supernatant was added at a final concentration of 50% v/v and cells were cultured for 4 days at 37°C in a humidified 5% CO_2 incubator. Cells were stained with viability dye (BioLegend, 50604715) and proliferation (CFSE dilution) was analyzed by flow cytometry.

RNA extraction

At the humane experimental endpoint, mice were sacrificed with an overdose of ketamine/xylazine (100/10 mg/kg) mix and immediately perfused with ice cold Ringer's solution (Sigma-Aldrich, 96724-100TAB). The brain was extracted, and a piece of tumor was immediately snap-frozen in liquid nitrogen for storage at -80°C. RNA was isolated from the frozen tumor pieces with a RNeasy Lipid Tissue Mini Kit (Qiagen, 74804) according to the manufacturer's instructions. RNA quantity was assessed with a NanoDrop 2000 spectrometer, while quality was confirmed via electrophoresis of samples in a 1% bleach gel as previously described (24).

qPCR

One microgram of RNA was reverse-transcribed using a QuantiTect Reverse Transcription Kit (Qiagen, 205313). cDNA was amplified using SsoAdvanced Universal SYBR-Green SuperMix (Bio-Rad, 1725272). Primers are listed in Supplemental Table 3. *Hprt* was used as an internal reference. PCR was monitored using a Viia7 sequence detection system (Applied Biosystems). The fold changes in the expression of the genes of interest were calculated as the mean values calculated using the $2^{-\Delta\Delta CT}$.

DNA extraction

At the experimental endpoint, mice were euthanized with an overdose of ketamine/xylazine (100/10 mg/kg) mix and immediately perfused with ice-cold Ringer's solution (Sigma-Aldrich, 96724-100TAB). Afterwards, the brain was removed, and a piece of tumor was immediately snap-frozen in liquid nitrogen for storage at -80°C. DNA was extracted from the frozen tumor pieces using the DNeasy Blood and Tissue Kit (Qiagen, 69504) according to the manufacturer's instructions. DNA quantity and integrity were assessed using the Qubit spectrometer.

Whole exome sequencing

A total of 1.5 µg of DNA (based on Qubit quantification) was mechanically fragmented on the Covaris M220 Focused-ultrasonicator (Covaris). Quality control was performed using the Agilent Bioanalyzer 4200 (Agilent Technologies) to ensure an average fragment size of 150–200 bp. End repair, A-tailing, adaptor ligation, and enrichment of DNA fragments were then performed. A 200- to 400-bp band was selected, and exome capture was performed using the TruSeq Exome Library Preparation kit (Illumina). The DNA library was quantified using the Qubit 3.0 Fluorometer (Invitrogen) and Agilent 4200 Bioanalyzer (Agilent Technologies). Samples were subjected to paired-end sequencing using the Illumina NextSeq 500 platform with a 150-bp read length. Raw BCL files generated by the sequencer were converted to FASTQ files for each sample. Sequence adapters and low-quality bases in raw reads were trimmed using Trimmomatic 0.39. Cleaned reads were then aligned to the GRCm38 reference genome using Sentieon 202112.01. Alignments were then sorted and PCR/Optical duplicates were marked.

Tumor mutational burden and microsatellite instability

Both whole-exome and whole-genome sequencing data for each sample were aligned to the GRCm38 reference genome using BWA-mem, including de-duplication and base recalibration steps provided by the nf-core Sarek pipeline (25). The Sarek pipeline also provided variant calling via Strelka2. Mutation counts from Strelka2 were normalized by the total bases sequenced in each sample to provide TMB, a measure of mutations per megabase sequenced. Microsatellite instability scores were computed for each sample using the MSIsensor-pro software (26). The reference genome was first processed to produce a baseline file with the "MSIsensor-pro scan" command, followed by scoring of microsatellite instability in the tumor-only sample context using the "MSIsensor-pro pro" command. MSIsensor-pro reports MSI score as the percentage of unstable microsatellite sites within all detected microsatellite sites in each sample. 4T1 and CT26 were used as positive for hypermutation phenotype.

Single-cell RNAseq and data analysis

Single-cell RNAseq analysis was done following the pipeline of Ross et al. (2024) (27). Briefly, tumors were dissociated into single-cell suspensions using an enzymatic mixture composed of

papain (0.94 mg/ml; Worthington, LS003120), EDTA (0.18 mg/ml; Sigma, E6758), cysteine (0.18 mg/ml; Sigma, A8199), and DNase I (60 μg/ml; Roche, 11284932001) in 2 ml HBSS (Gibco, 14175-095). Single-cell RNA sequencing using the Chromium 3' V3 platform (10x Genomics) was performed as previously described (28). Raw fastq files were aligned to the mouse genome reference mm10, which was customized to include the *Rfp* and *RCAS* sequences. This alignment was carried out using the CellRanger v5.0.0 from 10x Genomics. The count matrices generated by the CellRanger algorithm underwent additional filtering to exclude cells that had a gene count below 400, a UMI (unique molecular identifiers) count below 1000, and mitochondrial gene expression exceeding 25%. Hereafter, data processing and analysis were performed using the Seurat R package v4.2.2 (29) and figures were generated using ggplot2 (v 3.5.0). The normalization step was performed using the NormalizeData function with the normalization method parameter set to 'LogNormalize'. Dimensionality reduction was executed on the top 2000 variable features using the FindVariableFeatures, ScaleData, and RunPCA functions. UMAPs were generated using the top 15 principal components. The R package Harmony (v0.1.0) (30) was used to correct batch effects that arose from technical variations between the replicates. *De novo* clustering was performed using the Louvain algorithm (31) at various resolutions (0.2, 0.8, 1.2, 2, 3, 5, 8) on the SNN graph space. High-level annotation of cell classes were performed iteratively and was semi-supervised. This involved assigning *de novo* discovered clusters to specific cell classes based upon the expression of known marker genes. Doublets were removed from analyses by searching for clusters of cells that expressed canonical markers for two or more different cell types, along with a higher number of genes/UMI. We generated density plots of cells using UMAP coordinates for each condition with the LSD R package (https://doi.org/10.1038/s41467-023-40398-4). For presenting the neoplastic cellular state in mGBM at the subtype level, we used mouse ortholog genes of the human gene sets defined in and calculated the AddModuleScore for each mouse ortholog signature (32). The cellular state was visualized using a two-dimensional scatter plot, as previously described in (32). A sample manifest with sample ID, driver mutations included, and number of cells can be found in Supplemental Table 4.

GENIE data analysis

The dataset was refined using the accompanying metadata to restrict the analysis to primary, IDH wild-type (WT) adult GBM cases originating from three institutions: Memorial Sloan Kettering Cancer Center (MSK), Dana-Farber Cancer Institute (DFCI), and the University of California San Francisco (UCSF) —resulting in a cohort of 3,742 patients (GENIE-GBM cohort).

To investigate signals of positive selection, we used the dNdSloc function from the dndscv R package (33). Among the top 100 most frequently altered genes in the GENIE-GBM cohort, we selected only those that showed statistically significant positive selection (qall_loc < 0.05). The dN/dS ratios for these genes were visualized and stratified by mutation type (missense and truncating mutations).

To assess whether mutation type distribution could reflect functional gene classification, we analyzed the top 100 frequently altered genes from the GENIE-GBM cohort. For each gene, we calculated the proportions of specific mutation types: (1) non-synonymous mutations among all coding mutations, (2) truncating mutations (truncating + splice-site) among non-synonymous mutations, and (3) pathogenic missense mutations among non-synonymous mutations. SIFT predictions (34) were used for functional annotation in this step, and principal component analysis (PCA) was performed using the resulting mutation-type metrics.

After confirming discrepancies between SIFT and AlphaMissense annotations (35), we based all downstream analyses on AlphaMissense predictions. TMB was then compared across mutation categories for each MMR gene. TMB was defined as the number of non-synonymous mutations (filtered by variant allele frequency > 0.05 and tumor total depth > 20), divided by the sequencing coverage for each sample. Hypermutation status was defined as follows: Ultra-hypermutant: TMB > 100, Hypermutant: 10 < TMB ≤ 100, and Non-hypermutant (normal): TMB ≤ 10.

To evaluate the association between MMR gene mutations and the hypermutant (HM) phenotype, we dichotomized tumors as HM vs. non-HM based upon a TMB cutoff of 10 and performed Fisher's exact test. MSI burden was compared using the Mann–Whitney U test. The incidence of the HM phenotype in mutant vs. WT cases was reported as fold change.

For multivariate analysis, we first confirmed that no multicollinearity existed among the MMR genes. LASSO regression was then performed to identify genes most strongly associated with the HM phenotype. PMS2 was excluded at this step, and the remaining five MMR genes were used in the final multivariate logistic regression analysis.

For statistical Analysis of Tumor Mutational Burden (TMB) by Genotype for Figure 1D.

This analysis aims to compare TMB distributions among four MMR gene mutant genotypes (missense-benign, missense-pathogenic, splice-site, and truncating) and wild type (WT).

1. Group Comparison

Initial normality testing with the Shapiro-Wilk test for each of the five genotype groups (per gene) suggested that the data did not consistently meet the normality assumption required for a parametric test (e.g. One-way ANOVA). ('shapiro_test_result' sheet in excel file) - Supporting Data values for Figure 1D.

Therefore, we used the non-parametric Kruskal-Wallis test to compare to compare TMB distributions across five distinct genotypes (mis-benign, mis-pathogenic, splice, truncating, and WT).

As the Kruskal-Wallis test yielded statistically significant p-values for all MMR genes, we conducted a post-hoc analysis to determine which mutant genotype(s) significantly differed from the WT. ('kruskal_wallis_test_result' sheet in excel file)- Supporting Data values for Figure 1D

2. Post-Hoc Analysis

Pairwise Wilcoxon rank-sum tests were conducted to compare each mutant genotype against the WT. P-values were subsequently adjusted using the Bonferroni method for multiple comparisons. The specific comparisons were omitted where only a single case of a mutant was present (e.g., splice mutation in *MSH6* and *PMS2*). Consequently, the number of comparisons used for the Bonferroni adjustment was N=4 for MSH2 and *MLH1* (four comparisons) and N=3 for MSH6 and PMS2 (three comparisons). ('pairwise_wilcox_padj_by_bonferroni' sheet in excel file) - Supporting Data values for Figure 1D.

TCGA-GBM analysis

TCGA-GBM data were downloaded using TCGAbiolinks (36) and BiocOncoTK (37). Using provided metadata and mutation profiles, IDH WT GBM samples with primary presentation were selected for downstream analysis (n=284). TMB was defined as the number of non-synonymous mutations divided by the sequencing coverage for each sample.

Spectral flow cytometry

At the humane experimental endpoint, tumor-bearing mice were anesthetized with an overdose of ketamine/xylazine (100/10 mg/kg) and perfused with Ringer's solution and tumors were processed

according to a published protocol (28). Single-cell suspensions were passed through 70μm cell strainers, centrifuged, and resuspended in 30% Percoll (GE Healthcare, 17-0891-01) solution containing 10% FBS (Hyclone, SH30396.03). Cells were separated by centrifugation at 800*g* for 15 minutes at 4°C. The supernatant was carefully removed to discard debris and lipids. The cells were then washed in cold PBS and resuspended in RBC lysis buffer (BioLegend, 420301) for 1 min at 37°C. Cells were transferred to an Eppendorf tube and washed once with FACS buffer (DPBS with 0.5% BSA) and blocked with 100 μl of blocking solution (2% FBS, 5% normal rat serum, 5% normal mouse serum, 5% normal rabbit serum, 10 μg/ml anti-FcR (BioLegend, 101319) and 0.2% NaN_3 in DPBS) on ice for 15 minutes. Cells were then stained with antibodies on ice for 30 minutes and washed with PBS. The cells were subsequently incubated in 100 viability dye (Zombie UV; BioLegend, 423108; 1:800) at room temperature for 10 min. The cells were washed and fixed with fixation buffer (eBioscience, 00-5223-56) for 30 mins. Cells were then permeabilized with a permeabilization buffer (eBioscience, 00-8333-56) before staining with the intracellular markers. The cells were washed and stored at 4°C until analysis. For the analysis of immune cell populations within blood, 50 μL of blood was collected one day before starting treatment and on treatment day 7 when mice were treated with TMZ. For KL-50 experiments, 50 μL of blood was collected one day before starting treatment, and on treatment day 12. Blood was stained with antibodies for 15 minutes at room temperature in darkness; red blood cells were lysed using RBC lysis buffer (BioLegend, 420301) for 10 minutes at room temperature. Afterwards, 25 μL of CountBright™ Plus Absolute Counting beads (Invitrogen, C36995) were added and samples were stored at 4 °C until analysis. Antibodies used in this study are listed in Supplemental Table 1. All data were collected on a Cytek Aurora spectral flow cytometer. Data were analyzed using FlowJo 10 software (Tree Star Inc.).

TUNEL assay

Terminal deoxynucleotidyl transferase-mediated dUTP Nick End Labelling (TUNEL) assays were used for quantification of apoptotic cells following in VEH- and KL-50-treated *WT* and *Msh2 HET* tumors. Apoptotic cells characterized by DNA fragmentation were detected using an Invitrogen Click-iT Plus TUNEL Assay for *in situ* Apoptosis Detection kit (Fisher, C10617) following manufacturer's instructions. The nuclei were counter-stained with 40,6-diamidino-2-phenylindole (DAPI; Sigma, D9542). Five slides from each culturing condition were examined, and four different fields of view were acquired for each slide. Fluorescence and Differential Interference Contrast (DIC) images were taken on a Leica SP8 confocal microscope. Images were analyzed and quantified with FIJI, with TUNEL+ cells appearing to be bright green.

Supplemental table 1. Antibodies used in the study.

Antibody	Application	Specificity	Manufacturer	Catalog Number
IBA1	IHC	Mouse	Wako	019-19741
GFAP	IHC	Mouse	Cell signaling	CST3670
OLIG2	IHC	Mouse	Sigma	AB9610
pH3	IHC	Mouse	Millipore	06-570
CD31	IHC	Mouse	Dianova	DIA-310
CD68	IHC	Mouse/Human/Rat	Abcam	AB303565
CD206	IHC	Mouse/Human/Rat	Cell signaling	CST24595
Elastase	IHC	Mouse/Human	Bioss	Bs-6982R
P2RY12	IHC	Mouse	AnaSec	AS35043A
MSH2	IHC, IF, Western Blot	Mouse	Cell signaling	CST2017
MSH6	IHC, IF, Western Blot	Mouse	Abcam	AB92471
Alexa Fluor 594	IF	Anti-rabbit	Jackson ImmuneResearch	711-587-003
Alexa Fluor 555	IF	Anti-rabbit	Life Technologies	A31572
MGMT	Western blot	Mouse	Santa Cruz Biotechnology	Sc-166528
b-Actin	Western blot	Mouse	Cell signaling	CST3700
GAPDH	Western blot	Mouse	Santa Cruz Biotechnology	Sc-66163
CD45-FITC	Flow Cytometry	Mouse	BioLegend	103107
CD45-V450	Flow Cytometry	Mouse	BD Biosciences	560501
B220-BV605	Flow Cytometry	Mouse	BioLegend	103243
CD101-APC	Flow Cytometry	Mouse	Invitrogen	17101180
CD103-BUV395	Flow Cytometry	Mouse	BD Biosciences	748253
CD11b-PerCP-Cy5.5	Flow Cytometry	Mouse	BD Biosciences	550993
CD11c-APC	Flow Cytometry	Mouse	BD Biosciences	550281
CD24-BUV496	Flow Cytometry	Mouse	BD Biosciences	612953
CD3-PE-dazzle	Flow Cytometry	Mouse	BioLegend	100348
CD4-APC-Cy7	Flow Cytometry	Mouse	BioLegend	100526
CD49d-PE-dazzle	Flow Cytometry	Mouse	BioLegend	103625
CD8-BV510	Flow Cytometry	Mouse	BioLegend	100752
CD8-Pe-Cy5	Flow Cytometry	Mouse	BD Biosciences	553034
CX3CR1-BV650	Flow Cytometry	Mouse	BioLegend	149033
CXCR2-PE	Flow Cytometry	Mouse	BioLegend	149609

F4/80-BV711	Flow Cytometry	Mouse	BioLegend	123147
Foxp3-FITC	Flow Cytometry	Mouse	Invitrogen	11-5773-82
Grzm B-PE	Flow Cytometry	Mouse	Invitrogen	12-8899-41
IA/IE-Alexa700	Flow Cytometry	Mouse	BioLegend	107622
Ly6C-PE-Cy7	Flow Cytometry	Mouse	BD Biosciences	560593
Ly6G-V450	Flow Cytometry	Mouse	BD Biosciences	560603
NK1.1-BV711	Flow Cytometry	Mouse	BD Biosciences	740663
PD-L1-BV605	Flow Cytometry	Mouse	BD Biosciences	745135
PD1-BV785	Flow Cytometry	Mouse	BioLegend	135225
Tim-3-PE-Cy7	Flow Cytometry	Mouse	Invitrogen	25-5870-82

Supplemental table 2. Antibodies used in the Orion study.

Antibody	ArgoFluor Dye	Species	Clone / Type	Catalog Number	Supplier
CD163	Argo520	Rabbit	EPR19518	63-1001-501	RareCyte
CD31	Argo555L	Rabbit	D8V9E	63-1053-501	RareCyte
CD3ε	Argo548	Rabbit	D4V8L	63-1003-501	RareCyte
IBA1	Argo660L	Rabbit	EPR16589	62-1241-801	RareCyte
CD11c	Argo624	Rabbit	D3V1E	62-1077-601	RareCyte
OLIG2	Argo662	Rabbit	EPR2673	ab220796	Abcam
F4/80	Argo676	Rabbit	D2S9R	63-1015-601	RareCyte
CD8α	Argo698	Rabbit	EPR21769	63-1039-601	RareCyte
Foxp3	Argo706	Rabbit	D6O8R	63-1009-701	RareCyte
PD-1	Argo724	Rabbit	EPR20665	63-1025-701	RareCyte
Ki-67	Argo760	Rabbit	D3B5	62-1122-701	RareCyte
CD4	Argo812	Rabbit	EPR6855	62-1095-801	RareCyte
GFAP	Argo845	Rabbit	2.2B10	61-1241-801	RareCyte

Supplemental table 3. qPCR primers used in the study. The Bio-Rad qPCR primers used in the study are listed with their catalog numbers.

Primer	Bio-Rad Catalog Number
Hprt	qMmuCED0045738
Mgmt	qMmuCID0009593

Supplemental table 4. Sample manifest for scRNA sequencing.

Sample ID	Genotype	Mutations	Number of cells sequenced
5310	Msh2 WT	PDGFB+shp53	6250
5709	Msh2 WT	PDGFB+shp53	14797
5726	Msh2 WT	PDGFB+shp53	2630
5783	Msh2 WT	PDGFB+shp53	14598
5713	Msh2 HET	PDGFB+shp53	13073
5725	Msh2 HET	PDGFB+shp53	10202
6027	Msh2 HET	PDGFB+shp53	9679
6283	Msh2 HET	PDGFB+shp53	8241
5711	Msh2 KO	PDGFB+shp53	13313
5742	Msh2 KO	PDGFB+shp53	4836
5781	Msh2 KO	PDGFB+shp53	19537

References

1. Reitmair AH, Schmits R, Ewel A, Bapat B, Redston M, Mitri A, et al. MSH2 deficient mice are viable and susceptible to lymphoid tumours. *Nat Genet.* 1995;11(1):64–70.
2. Edelmann W, Yang K, Umar A, Heyer J, Lau K, Fan K, et al. Mutation in the mismatch repair gene Msh6 causes cancer susceptibility. *Cell.* 1997;91(4):467–77.
3. Marino S, Vooijs M, van Der Gulden H, Jonkers J, and Berns A. Induction of medulloblastomas in p53-null mutant mice by somatic inactivation of Rb in the external granular layer cells of the cerebellum. *Genes Dev.* 2000;14(8):994–1004.
4. Kucherlapati MH, Lee K, Nguyen AA, Clark AB, Hou H, Jr., Rosulek A, et al. An Msh2 conditional knockout mouse for studying intestinal cancer and testing anticancer agents. *Gastroenterology.* 2010;138(3):993–1002 e1.
5. Lee PP, Fitzpatrick DR, Beard C, Jessup HK, Lehar S, Makar KW, et al. A critical role for Dnmt1 and DNA methylation in T cell development, function, and survival. *Immunity.* 2001;15(5):763–74.
6. Sawada S, Scarborough JD, Killeen N, and Littman DR. A lineage-specific transcriptional silencer regulates CD4 gene expression during T lymphocyte development. *Cell.* 1994;77(6):917–29.
7. Maekawa Y, Minato Y, Ishifune C, Kurihara T, Kitamura A, Kojima H, et al. Notch2 integrates signaling by the transcription factors RBP-J and CREB1 to promote T cell cytotoxicity. *Nat Immunol.* 2008;9(10):1140–7.
8. Yona S, Kim KW, Wolf Y, Mildner A, Varol D, Breker M, et al. Fate mapping reveals origins and dynamics of monocytes and tissue macrophages under homeostasis. *Immunity.* 2013;38(1):79–91.
9. Franklin KBJ, and Paxinos G. *The mouse brain in stereotaxic coordinates.* San Diego: Academic Press; 1997.
10. Hambardzumyan D, Amankulor NM, Helmy KY, Becher OJ, and Holland EC. Modeling Adult Gliomas Using RCAS/t-va Technology. *Transl Oncol.* 2009;2(2):89–95.
11. Herting CJ, Chen Z, Pitter KL, Szulzewsky F, Kaffes I, Kaluzova M, et al. Genetic driver mutations define the expression signature and microenvironmental composition of high-grade gliomas. *Glia.* 2017;65(12):1914–26.
12. Ozawa T, Riester M, Cheng YK, Huse JT, Squatrito M, Helmy K, et al. Most human non-GCIMP glioblastoma subtypes evolve from a common proneural-like precursor glioma. *Cancer Cell.* 2014;26(2):288–300.
13. Ross JL, Chen Z, Herting CJ, Grabovska Y, Szulzewsky F, Puigdelloses M, et al. Platelet-derived growth factor beta is a potent inflammatory driver in paediatric high-grade glioma. *Brain.* 2021;144(1):53–69.
14. Reilly KM, Loisel DA, Bronson RT, McLaughlin ME, and Jacks T. Nf1;Trp53 mutant mice develop glioblastoma with evidence of strain-specific effects. *Nat Genet.* 2000;26(1):109–13.
15. Pan Y, Smithson LJ, Ma Y, Hambardzumyan D, and Gutmann DH. Ccl5 establishes an autocrine high-grade glioma growth regulatory circuit critical for mesenchymal glioblastoma survival. *Oncotarget.* 2017;8(20):32977–89.
16. Mandal R, Samstein RM, Lee KW, Havel JJ, Wang H, Krishna C, et al. Genetic diversity of tumors with mismatch repair deficiency influences anti-PD-1 immunotherapy response. *Science.* 2019;364(6439):485–91.

17. Louis DN, Perry A, Wesseling P, Brat DJ, Cree IA, Figarella-Branger D, et al. The 2021 WHO Classification of Tumors of the Central Nervous System: a summary. *Neuro Oncol.* 2021;23(8):1231–51.
18. Lin JR, Chen YA, Campton D, Cooper J, Coy S, Yapp C, et al. High-plex immunofluorescence imaging and traditional histology of the same tissue section for discovering image-based biomarkers. *Nat Cancer.* 2023;4(7):1036–52.
19. Schmidt U, Weigert M, Broaddus C, and Myers G. Cell Detection with Star-convex Polygons. *arXiv:180603535.* 2018;MICCAI
20. van der Walt S, Schonberger JL, Nunez-Iglesias J, Boulogne F, Warner JD, Yager N, et al. scikit-image: image processing in Python. *PeerJ.* 2014;2:e453.
21. Sofroniew N, Lambert T, Bokota G, Nunez-Iglesias J, Sobolewski P, Sweet A, et al. napari: a multi-dimensional image viewer for Python (v0.6.6rc2). Zenodo. 2025;https://doi.org/10.5281/zenodo.17312776.
22. Nirmal AJ, and Sorger PK. SCIMAP: A Python Toolkit for Integrated Spatial Analysis of Multiplexed Imaging Data. *J Open Source Softw.* 2024;9(97).
23. Virtanen P, Gommers R, Oliphant TE, Haberland M, Reddy T, Cournapeau D, et al. SciPy 1.0: fundamental algorithms for scientific computing in Python. *Nature Methods.* 2020;17(3):261–72.
24. Aranda PS, LaJoie DM, and Jorcyk CL. Bleach gel: a simple agarose gel for analyzing RNA quality. *Electrophoresis.* 2012;33(2):366–9.
25. Garcia M, Juhos S, Larsson M, Olason PI, Martin M, Eisfeldt J, et al. Sarek: A portable workflow for whole-genome sequencing analysis of germline and somatic variants. *F1000Res.* 2020;9:63.
26. Jia P, Yang X, Guo L, Liu B, Lin J, Liang H, et al. MSIsensor-pro: Fast, Accurate, and Matched-normal-sample-free Detection of Microsatellite Instability. *Genomics Proteomics Bioinformatics.* 2020;18(1):65–71.
27. Ross JL, Puigdelloses-Vallcorba M, Pinero G, Soni N, Thomason W, DeSisto J, et al. Microglia and monocyte-derived macrophages drive progression of pediatric high-grade gliomas and are transcriptionally shaped by histone mutations. *Immunity.* 2024;57(11):2669–87 e6.
28. Chen Z, Soni N, Pinero G, Giotti B, Eddins DJ, Lindblad KE, et al. Monocyte depletion enhances neutrophil influx and proneural to mesenchymal transition in glioblastoma. *Nat Commun.* 2023;14(1):1839.
29. Hao Y, Hao S, Andersen-Nissen E, Mauck WM, 3rd, Zheng S, Butler A, et al. Integrated analysis of multimodal single-cell data. *Cell.* 2021;184(13):3573–87 e29.
30. Korsunsky I, Millard N, Fan J, Slowikowski K, Zhang F, Wei K, et al. Fast, sensitive and accurate integration of single-cell data with Harmony. *Nat Methods.* 2019;16(12):1289–96.
31. Subelj L, and Bajec M. Unfolding communities in large complex networks: combining defensive and offensive label propagation for core extraction. *Phys Rev E Stat Nonlin Soft Matter Phys.* 2011;83(3 Pt 2):036103.
32. Neftel C, Laffy J, Filbin MG, Hara T, Shore ME, Rahme GJ, et al. An Integrative Model of Cellular States, Plasticity, and Genetics for Glioblastoma. *Cell.* 2019;178(4):835–49 e21.
33. Martincorena I, Raine KM, Gerstung M, Dawson KJ, Haase K, Van Loo P, et al. Universal Patterns of Selection in Cancer and Somatic Tissues. *Cell.* 2017;171(5):1029–41 e21.

34. Sim NL, Kumar P, Hu J, Henikoff S, Schneider G, and Ng PC. SIFT web server: predicting effects of amino acid substitutions on proteins. *Nucleic Acids Res.* 2012;40(Web Server issue):W452–7.
35. Cheng J, Novati G, Pan J, Bycroft C, Zemgulyte A, Applebaum T, et al. Accurate proteome-wide missense variant effect prediction with AlphaMissense. *Science.* 2023;381(6664):eadg7492.
36. Colaprico A, Silva TC, Olsen C, Garofano L, Cava C, Garolini D, et al. TCGAbiolinks: an R/Bioconductor package for integrative analysis of TCGA data. *Nucleic Acids Res.* 2016;44(8):e71.
37. Carey V. BiocOncoTK: Bioconductor components for general cancer genomics. 2024.
