# Supplementary material for: Mismatch repair deficiency drives malignant progression and alters the tumor immune microenvironment in glioblastoma models
Source: J Clin Invest. 2025 Dec 23;136(6):e195189. doi: 10.1172/JCI195189 (PMC12987617; doi:10.1172/JCI195189)
Supplement: Unedited blot and gel images [file jci-136-195189-s088.pdf]

Full unedit blot for Supplemental figure 4A

Full unedit blot for Supplemental figure 4D

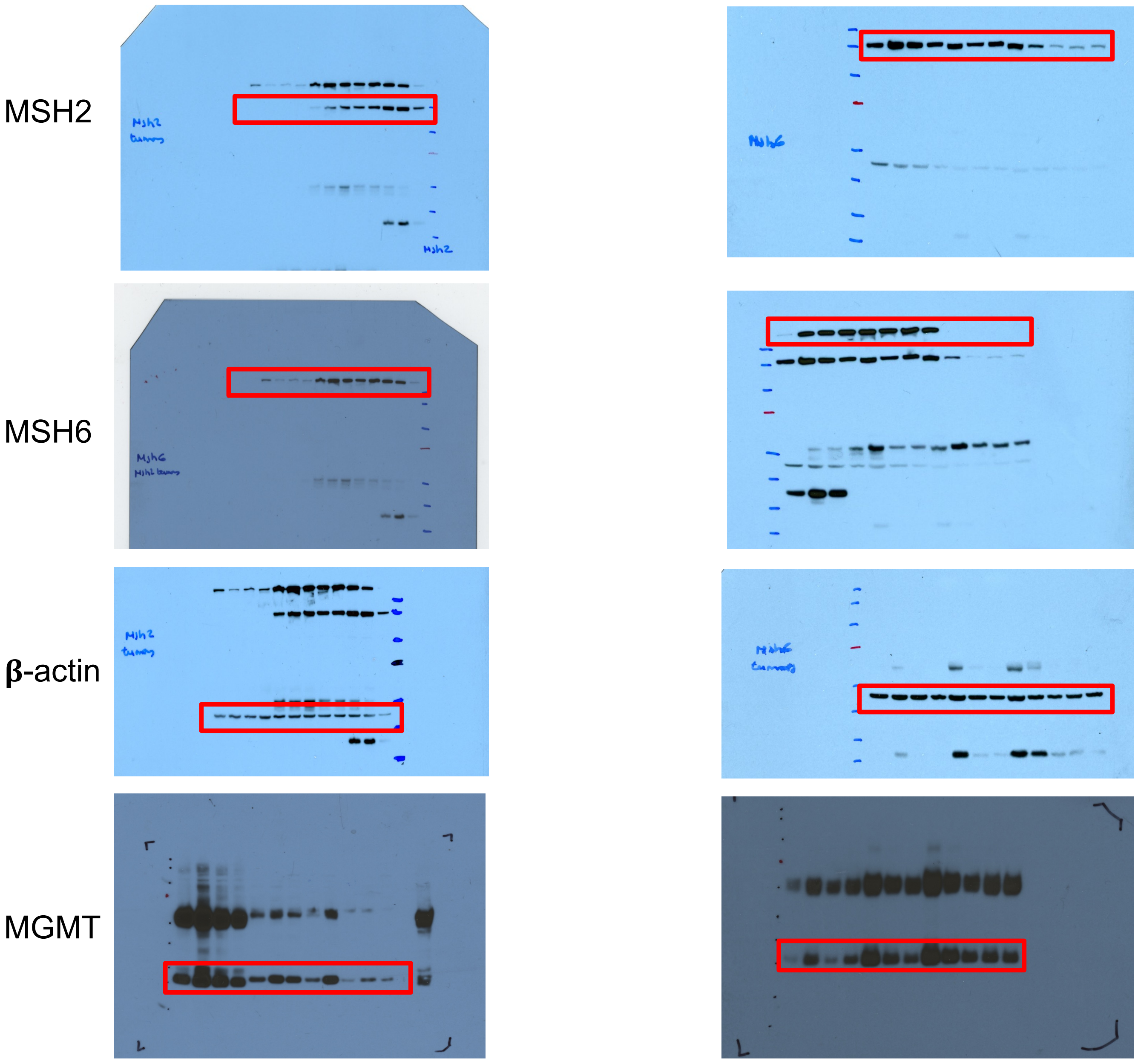

Full unedit blot for Supplemental figure 15B

MGMT

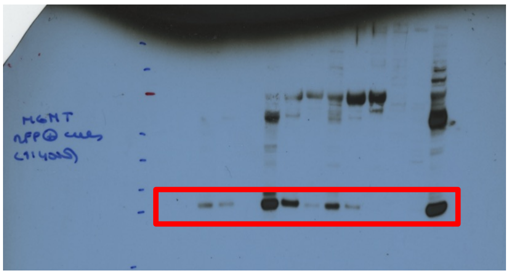

MGMT

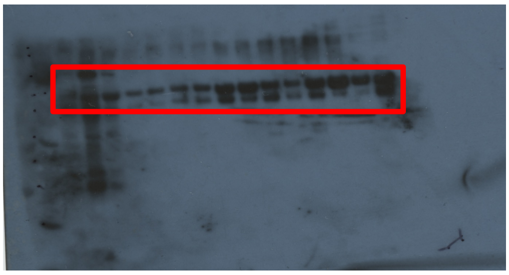

β-actin

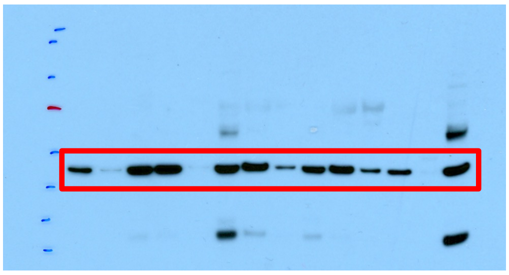

GAPDH

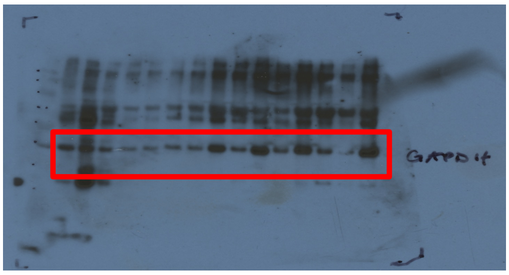

Full unedit blot for Supplemental figure 15C

Full unedit blot for Supplemental figure 15D

MSH2

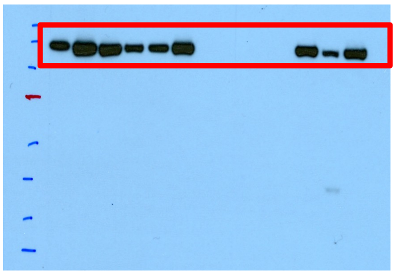

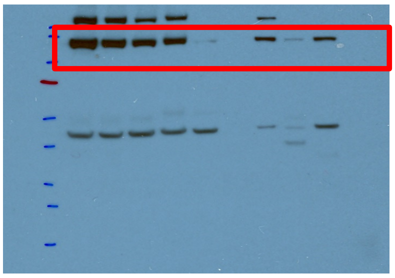

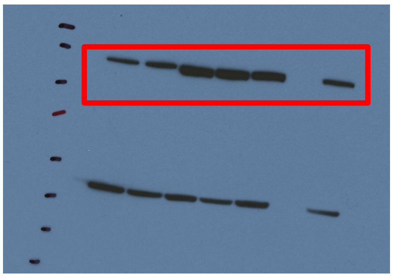

MSH6

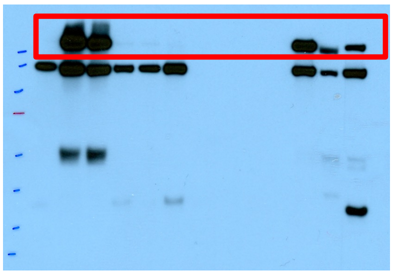

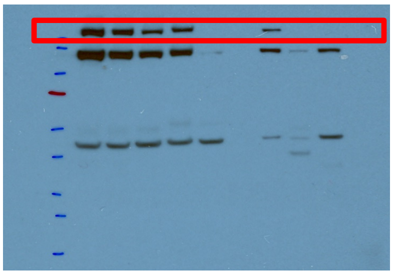

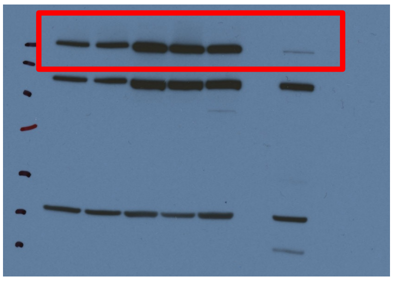

MGMT

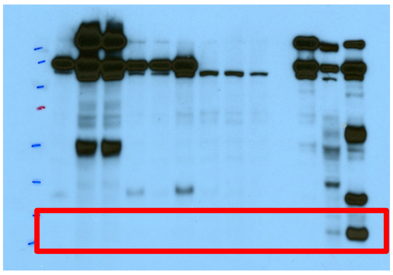

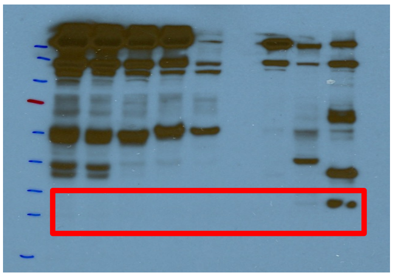

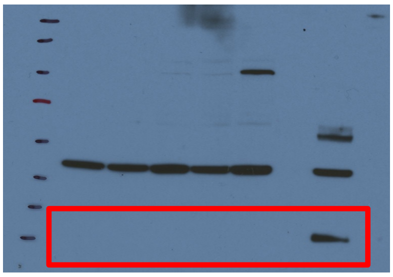

β-actin

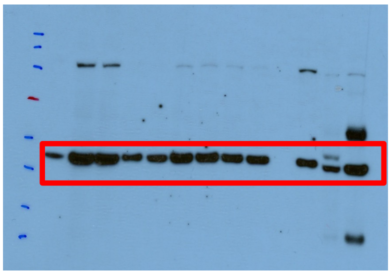

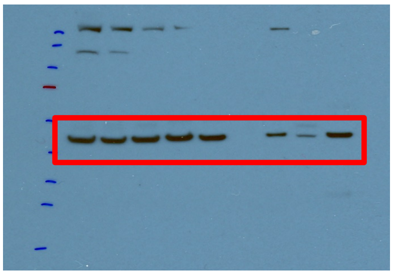

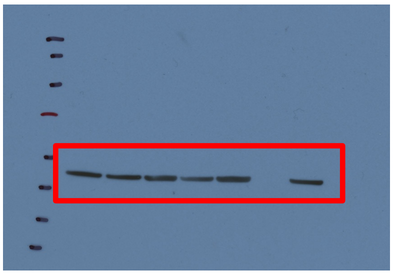

MSH2 cell lines

MSH6 cell lines
